# Techno‐Economically Viable, Rapid, and Scalable Sonochemical Synthesis of AgCl Nanocubes for CO_2_ Electroreduction

**DOI:** 10.1002/smll.74602

**Published:** 2026-07-16

**Authors:** Younghyun Chae, Hyunyoung Kim, Dongjin Kim, Woong Choi, Hyunwook Kim, Donggu Han, Dong Ki Lee, Wangyun Won, Ung Lee, Da Hye Won

**Affiliations:** ^1^ Clean Energy Research Center Korea Institute of Science and Technology Seoul Republic of Korea; ^2^ Division of Energy and Environmental Technology KIST School, University of Science and Technology (UST) Seoul Republic of Korea; ^3^ Department of Chemical and Biological Engineering Korea University Seoul Republic of Korea; ^4^ Department of Materials Science and Engineering Korea University Seoul Republic of Korea; ^5^ Department of Energy Engineering Gyeongsang National University Jinju Republic of Korea; ^6^ Department of Energy System Engineering Gyeongsang National University Jinju Republic of Korea; ^7^ Graduate School of Energy and Environment (Green School) Korea University Seoul Republic of Korea; ^8^ Department of Chemical and Biomolecular Engineering, Yonsei‐KIST Convergence Research Institute Yonsei University Seoul Republic of Korea

**Keywords:** electrochemical CO_2_ reduction (CO_2_RR), membrane electrode assembly (MEA), silver chloride (AgCl), sonochemistry, techno‐economic analysis (TEA)

## Abstract

The economic viability of the electrochemical CO_2_ reduction reaction relies not only on efficient catalytic performance but also on the scalability and cost‐efficiency of catalyst production. Herein, a rapid and scalable sonochemical synthesis of AgCl nanocubes (AgCl NCs), featuring efficient manufacturability, high CO selectivity, and long‐term stability is reported. In a membrane electrode assembly, AgCl NC maintained exceptionally high CO Faradaic efficiencies (>95%) during 200 h of continuous operation. Techno‐economic analysis (TEA) revealed that the outstanding performance of AgCl NC, coupled with its cost‐ and time‐efficient synthesis, significantly lowered the levelized catalyst cost by 40% and the CO production cost by nearly 50% relative to conventional Ag nanoparticle systems. By linking catalyst production directly to system‐level TEA outcomes, this study highlights manufacturability as a key yet often overlooked criterion in catalyst design and positions AgCl NC as a promising platform for industrial‐scale application.

## Graphical abstract

AgCl nanocubes are synthesized within minutes through a rapid and scalable sonochemical method. Combined with outstanding catalytic performance, the lower production cost demonstrates the overall cost‐effectiveness of AgCl nanocubes for CO_2_‐to‐CO electroreduction. These results highlight economic viability as a key consideration in catalyst development, providing a framework for practical CO_2_ conversion.

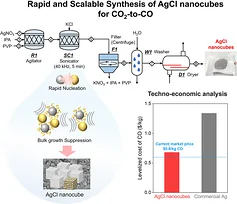

## 1 Introduction

The electrochemical CO_2_ reduction reaction (CO_2_RR) has gained significant attention as a carbon capture and utilization technology, supported by recent progress in electrolyzers, membranes, ionomers, and catalysts [1, 2, 3, 4, 5, 6]. Among various CO_2_ reduction products, CO is particularly promising owing to a simple reaction pathway, allowing high selectivity and productivity, and the resulting CO and H_2_ serve as versatile platform chemicals for diverse downstream applications [7, 8, 9, 10]. Building on these advancements, techno‐economic analyses (TEAs) have been increasingly employed to bridge laboratory‐scale research with practical implementation by evaluating product value, system costs, and system durability [11, 12, 13, 14, 15]. However, most TEA models rely on simplified assumptions that estimate the costs of catalysts, membranes, and electrodes as fixed percentages of the base equipment cost [16, 17, 18]. Moreover, these components are typically evaluated solely based on their catalytic performance, whereas the impact of their synthetic route or fabrication process on the overall cost, scalability, and industrial feasibility is often not comprehensively investigated, which compromises the accuracy of commercial viability assessments [19, 20, 21, 22]. To enable the transition from laboratory‐scale development to commercial CO_2_RR application, synthetic strategies and TEA frameworks should expand beyond performance‐oriented metrics to include manufacturing considerations.

Ag‐based materials are the most extensively studied CO production catalysts that exhibit excellent performances and serve as representative systems in CO_2_RR research [23, 24]. The development of Ag‐based catalysts has primarily been focused on controlling the key properties related to catalytic performance, such as particle size, morphology, and composition (Figure S1 and Table S1), leading to remarkable progress with recent reports achieving ampere‐level CO partial current densities (*j*_CO_) [25, 26, 27, 28]. However, most synthetic routes suffer from low throughput and limited yields, typically restricted to bench‐scale production which severely limits industrial scalability and cost‐effectiveness [25, 29, 30, 31, 32]. Although these synthesis‐related parameters affect scalability and cost, they remain largely unaccounted for in existing TEA models, highlighting the need for catalyst designs focused on optimizing manufacturability and system integration toward practical application.

In this study, we developed a rapid and scalable synthetic method for obtaining uniform AgCl nanocubes (AgCl NCs). The catalyst exhibited an excellent CO_2_RR performance and offered system‐level cost advantages, effectively bridging synthetic chemistry with economic considerations in catalyst development. Sonochemical treatment was applied for the synthesis of the catalyst, which was completed within 5 min. The developed approach enabled the gram‐scale production of AgCl NC with well‐defined cubic morphology through the combined effects of a spontaneous base reaction, sonic energy, and capping agents. AgCl NC showed superior CO production selectivity compared to commercial Ag benchmarks (Ag nanoparticles, Ag NPs), achieving *j*_CO_ values exceeding 750 mA cm^−2^ in a membrane electrode assembly (MEA) cell. By designing a scalable synthesis and leveraging the AgCl NC performance, we achieved ≈40% cost reduction compared to that of conventional Ag NP systems. This approach encompasses an underexplored aspect of CO_2_RR cost evaluation, enabling a more realistic evaluation of catalyst viability for industrial application.

## 2 Results and Discussion

**FIGURE 1:**
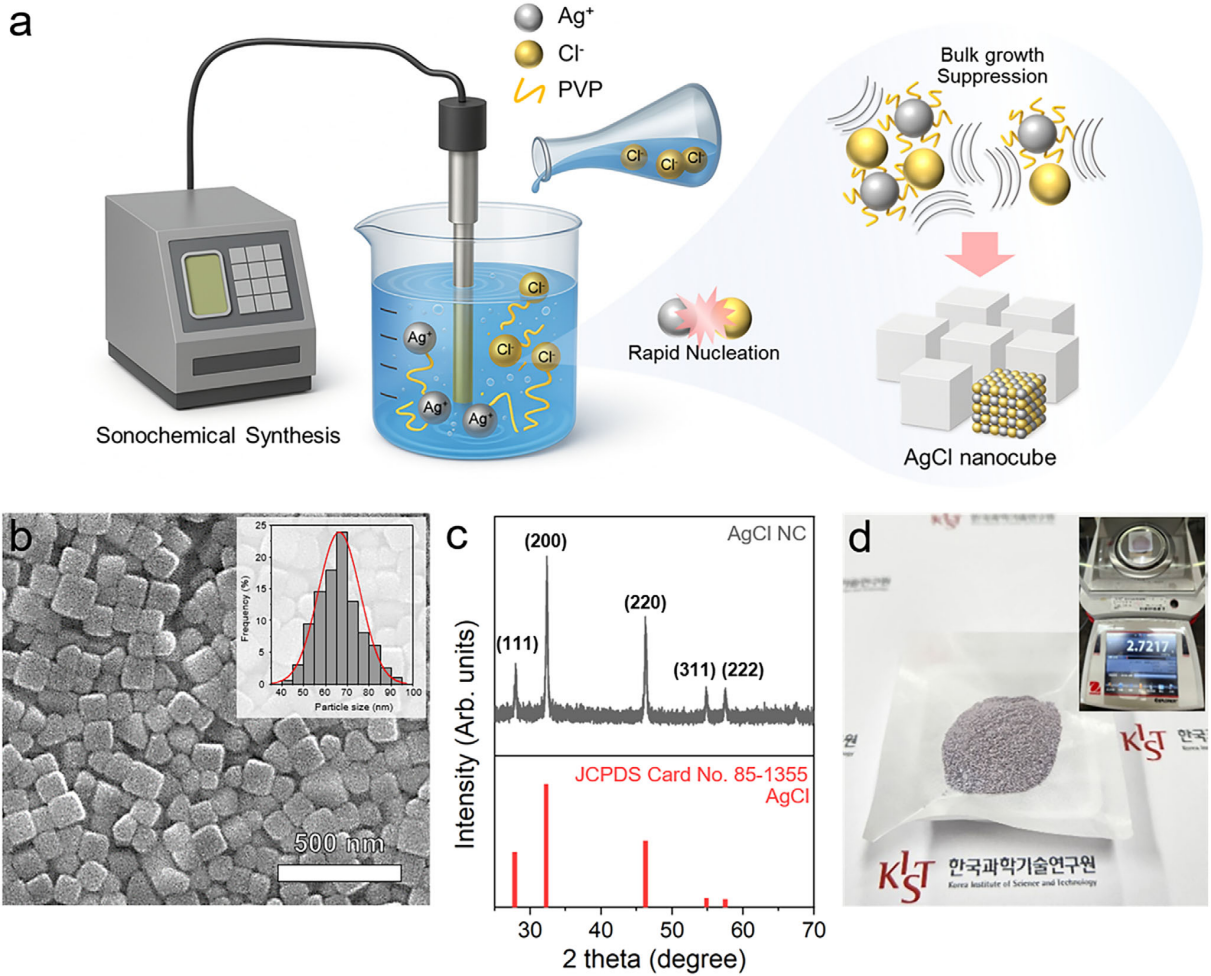
(a) Schematic of the synthesis of AgCl NC. Certain graphical elements in the schematic were enhanced using generative AI to create a three‐dimensional effect. (b) SEM image of AgCl NC; the inset shows the particle size distribution. (c) XRD pattern of AgCl NC compared with the reference. (d) Photograph of the as‐synthesized AgCl NC powder from a large‐scale batch; the inset shows the weighed AgCl NC powder on a balance.

AgCl‐derived Ag nanostructures, often referred to as Ag‐coral, demonstrate high CO selectivities in the CO_2_RR [29, 33, 34, 35]. Fabrication of such Ag‐coral structures typically entails the preparation of metallic Ag electrodes, followed by electrochemical oxidation in KCl electrolyte for 12 h to produce AgCl, and subsequent in situ electroreduction under CO_2_RR conditions [29]. However, this method is time‐consuming and inherently limited in scalability, as the catalysts are grown directly on the substrate, which restricts the batch size and complicates mass production. To overcome these limitations, we developed a sonochemistry‐based approach for the rapid and scalable preparation of AgCl nanostructures in powder form. In addition to shortening the synthesis time by eliminating the lengthy oxidation step, the method also allows for flexible electrode fabrication. The spontaneous formation of AgCl occurs when Ag^+^ and Cl^−^ ions are mixed in solution, offering a simple synthetic route that avoids electrochemical procedures. However, the extremely low solubility of AgCl leads to rapid precipitation upon mixing, making it difficult to control the particle size and morphology [36, 37, 38]. This often results in uncontrolled nucleation and aggregation into irregular, micro‐sized particles. To address this issue, we introduced polyvinylpyrrolidone (PVP) as a capping agent under sonochemical conditions, which suppressed excessive particle growth and enabled the formation of uniform AgCl NC (Figure 1a). Remarkably, the entire synthesis was completed within 5 min. When AgNO_3_ and KCl precursor solutions containing excess PVP were mixed under sonication, rapid nucleation occurred, but in this case, the sonic energy limited particle growth, and surface‐adsorbed PVP promoted the formation of uniform AgCl NC. The prepared AgCl NC exhibited a well‐defined cubic morphology with an average size of 66.2 ± 9.5 nm; the phase was confirmed to be pure AgCl through X‐ray diffraction (XRD) analyses (Figure 1b,c). The well‐defined cubic morphology of AgCl NC was attributed to the preferential adsorption of PVP, which stabilizes the (100) facets. Optimization of the PVP concentration enhanced this facet stabilization. In contrast, AgCl particles synthesized without PVP exhibited irregular morphology and submicron sizes even under sonication (denoted as AgCl bulk, Figure S3). The synthesis optimization details are provided in Supporting Information, as Experimental Section and Note S1.

Importantly, this method proved to be readily scalable. We attempted to scale up the synthesis in our laboratory by increasing the batch size to 320, 480, 640, 800, and 1000 mL. A key factor during scale‐up is adjusting the sonic energy intensity according to the increasing batch size. Using this approach, we successfully synthesized up to 2.72 g of AgCl NC in a single batch without compromising the well‐defined NC structure (Figure 1d). Because sonic energy can be readily amplified by applying larger sonic tips or by operating multiple tips simultaneously, we expect that this synthesis method is amenable to further scale‐up beyond our current laboratory setup, provided the necessary infrastructure is available (Figures S6 and S7).

**FIGURE 2:**
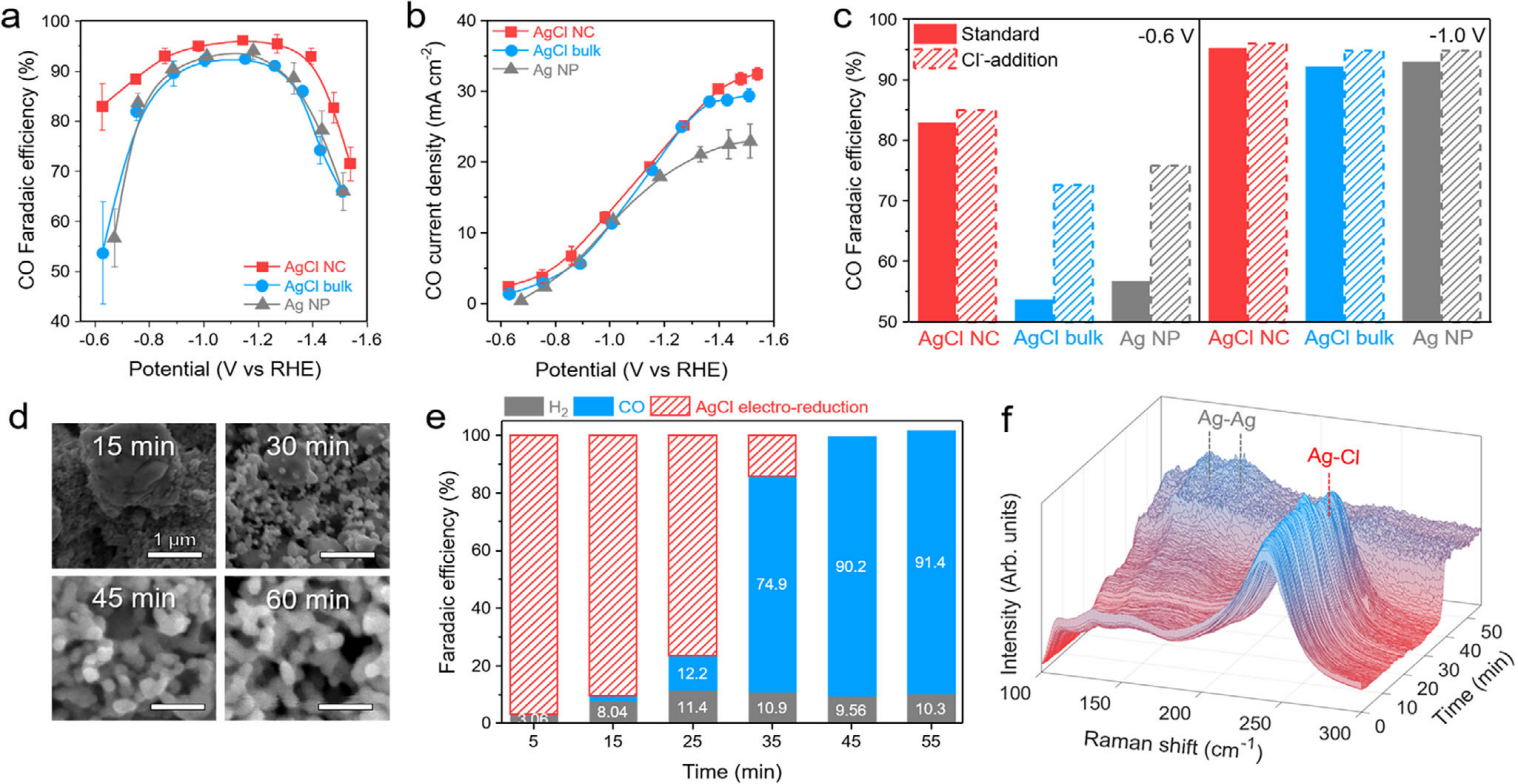
Electrochemical properties of the AgCl‐based catalysts. (a) FE_CO_ and (b) *j*_CO_ measured in an H‐type cell. (c) Comparison of FE_CO_ attained in 0.1 M KHCO_3_ (standard conditions) and in 0.09 M KHCO_3_ + 0.01 M KCl (Cl^−^‐added conditions) at –0.6 V and –1.0 V_RHE_. (d) SEM images of AgCl NC electrodes after electroreduction at 5 mA cm^−2^ for 15, 30, 45, and 60 min. (e) CO_2_RR and HER FEs of AgCl NC during electroreduction at 5 mA cm^−2^. (f) Time‐resolved 3D Raman spectra of AgCl NC obtained during CO_2_RR at a reductive current density of 5 mA cm^−2^. The Raman bands at 245 cm^−1^ and those at 135 and 165 cm^−1^ are assigned to Ag–Cl and Ag–Ag vibrations, respectively. Error bars represent the standard deviation calculated from at least 3 data points.

The CO_2_RR performance of AgCl NC was investigated by spray‐coating the catalyst ink onto carbon paper to prepare the working electrode, which was then applied in a three‐electrode H‐type cell containing CO_2_‐purged 0.1 M KHCO_3_ as the electrolyte (Figure 2a,b; Figure S8). To evaluate the effects of the uniform morphology and distinct surface chemical properties of AgCl NC on the catalytic performance, AgCl bulk and commercial Ag NP with different physical and chemical properties, were additionally prepared as control electrodes (Figure S9). Prior to CO_2_RR measurements, the AgCl‐based catalysts were electrochemically activated at a reductive current density of 5 mA cm^−2^ for 60 min, leading to the formation of the targeted Ag‐coral structure (Notes S2 and S3). Interestingly, AgCl NC underwent gradual surface transformation to Ag‐coral, becoming clearly visible after 45 min, whereas AgCl bulk rapidly collapsed into aggregated particles within 15 min (Figure 2d; Figure S10). During the activation process, we monitored the gas products and observed that the total Faradaic efficiency (FE) for CO and H_2_ remained substantially below 100% for the first 45 min, indicating that a portion of the charge was consumed by the reduction of AgCl NC (Figure 2e); no other gaseous or liquid products were detected during this period (Figure S11). In contrast, the FE of AgCl bulk remained significantly below 100% only during the first 15 min, and that of commercial Ag NP immediately reached approximately 100% under the same conditions (Figure S12). After activation, they exhibited stable and reproducible CO_2_RR performance. XRD analysis confirmed that both activated AgCl NC and AgCl bulk were fully reduced to metallic silver (Figure S13). The differences in activation behavior appeared to be related to the electrochemically active surface area. The activated AgCl NC exhibited the largest surface area among the prepared catalysts, as indicated by its highest specific capacitance (7.7 mF cm^−2^), followed by Ag NP (4.21 mF cm^−2^) and AgCl bulk (1.28 mF cm^−2^) (Figure S14).

The CO_2_RR was conducted by applying a constant potential in the range of −0.6 to −1.6 V_RHE_ (V vs. reversible hydrogen electrode, RHE). AgCl NC exhibited outstanding CO selectivity, with CO FE (FE_CO_) exceeding 90% across a broad potential window and peaking at 96.2% at −1.14 V_RHE_. Notably, AgCl NC delivered FE_CO_ values of >80% even at −0.63 V_RHE_, demonstrating exceptional performance at low‐overpotentials (Figure 2a; Figure S8). In contrast, although AgCl bulk and Ag NP attained FE_CO_ above 90% at their respective peaks, they displayed substantially lower CO selectivity at −0.6 V_RHE_ (50% and 56.7%, respectively). Furthermore, they exhibited high FE_CO_ values within a narrower potential range than that observed for AgCl NC. In terms of CO productivity, AgCl NC outperformed both controls, delivering the highest *j*_CO_ across the entire potential range (Figure 2b). This superior performance of AgCl NC can be attributed not only to its larger surface area but also to its unique surface characteristics that intrinsically provide more active sites.

In addition to morphology, the surface chemical composition of AgCl NC emerged as a critical factor affecting the CO_2_RR performance. X‐ray photoelectron spectroscopy (XPS) revealed that the Cl signal disappeared from AgCl bulk after electrolysis, whereas AgCl NC retained a shifted Cl 2p peak, indicating the presence of a distinct surface Cl species. Notably, the surface‐retained Cl appears to play a distinctive catalytic role by facilitating CO_2_ adsorption and modulating the local electronic environment surrounding the catalyst surface [39, 40]. To assess the catalytic relevance of Cl, CO_2_RR was evaluated under standard (Cl^−^‐free 0.1 M KHCO_3_) and Cl^−^‐containing (0.09 M KHCO_3_ + 0.01 M KCl) electrolytes within the same potential range as that applied above (Figure 2c; Figure S18). At –0.6 V_RHE_, corresponding to the onset region of the CO_2_RR, both AgCl bulk and Ag NP showed increased FE_CO,_ from 53.7% to 72.7% and from 56.7% to 75.9%, respectively. This behavior indicates a positive shift in the onset potential, presumably due to Cl^−^‐induced enhancement of CO_2_ activation. Notably, the improved FE_CO_ was maintained even at –1.0 V_RHE_, where the CO_2_RR selectivity reaches its peak. However, AgCl NC exhibited only slight changes across the entire potential range, consistent with the presence of pre‐existing surface‐retained Cl. This trend is further corroborated by the Tafel analysis, which showed decreased apparent Tafel slopes for AgCl bulk and Ag NP upon KCl additions, whereas the apparent Tafel slope of AgCl NC remained essentially unchanged (Figure S19).

To clarify the structural origin of the distinctive CO_2_RR behavior of AgCl NC, we investigated its phase evolution and surface chemical environment during activation and electrolysis. In situ Raman spectroscopy conducted during activation revealed that the Ag–Cl band at 245 cm^−1^ gradually diminished, while new bands assigned to Ag–Ag vibrations emerged at 135 and 165 cm^−1^, indicating the progressive reduction of AgCl to metallic Ag (Figure 2f; Figure S20). This phase transformation to metallic Ag was confirmed by time‐dependent ex situ XRD analysis (Figure S21). Complementary XPS analysis revealed a negative shift in the Cl 2p binding energy and a persistent Cl signal after electrolysis, indicating that the residual Cl species remained in a chemical environment distinct from that of pristine AgCl (Figure S22). Comparison with Cl‐containing reference compounds suggests that the residual Cl species are associated with the PVP‐modified Ag interface, while the precise coordination motif remains unresolved because the molecular references do not fully reproduce the actual PVP–Ag interface (Figure S23). Furthermore, the Ag 3d spectra exhibited a noticeable binding energy shift after activation. Compared with metallic Ag (Ag NP), pristine AgCl NC exhibited an Ag 3d binding energy approximately 0.6 eV lower, whereas the activated and post‐CO_2_RR AgCl NC remained approximately 0.4 eV lower despite a positive shift of approximately 0.2 eV upon activation. This result indicates that the AgCl NC‐derived surface remained electronically distinct from metallic Ag, possibly due to interactions with the surface‐retained Cl species. Although the Ag 3d shift alone is insufficient to determine the oxidation state or electron density of Ag, its combination with the Cl 2p XPS results supports the possibility of an electronic interaction between the residual Cl species and the PVP‐modified Ag interface. Taken together, these results support the proposed contribution of surface‐retained Cl species to the favorable interfacial environment and enhanced CO_2_RR performance of AgCl NC.

**FIGURE 3:**
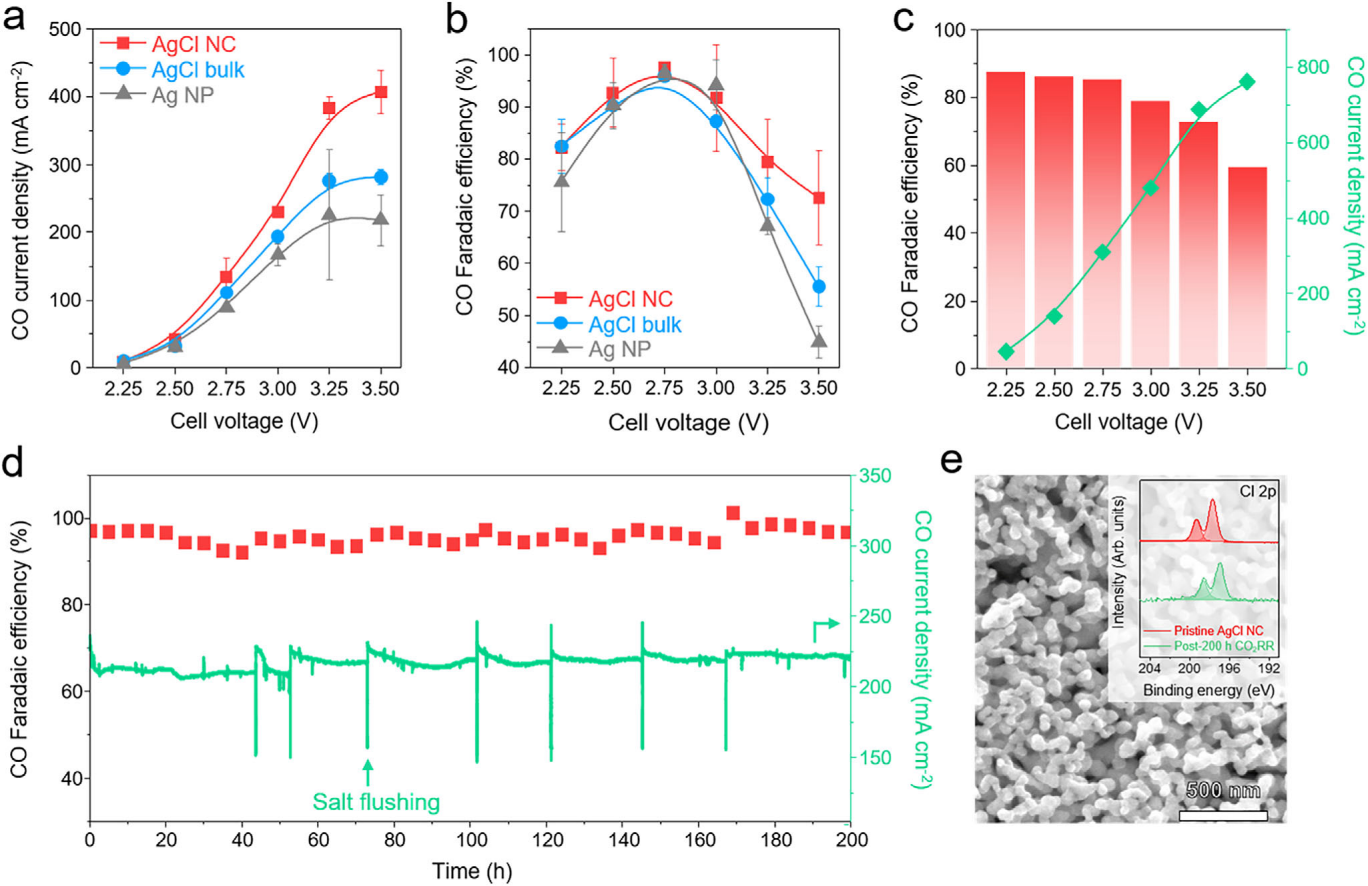
CO_2_RR performance and stability of the catalysts evaluated in an MEA cell. (a) *j*_CO_ and (b) FE_CO_ of the catalysts measured in an MEA cell. (c) CO_2_RR performance of AgCl NC in 0.5 M KHCO_3_ and at 60°C in the MEA cell. (d) Total current density and FE_CO_ variation during long‐term electrolysis by AgCl NC at 3.0 V in the MEA cell. (e) SEM image of AgCl NC after 200 h of electrolysis, with an inset showing the Cl 2p XPS spectra of the AgCl NC electrode before and after the reaction. Error bars represent the standard deviation calculated from at least 3 data points.

AgCl NC was subsequently evaluated in an MEA cell, a configuration suitable for achieving high CO productivity under gas‐fed CO_2_ supply. Catalytic performance was evaluated under a cell voltage range of 2.25–3.5 V (Figure 3a,b; Figure S24). Unlike in the H‐type cell, AgCl NC underwent rapid transformation to Ag‐coral in the MEA cell within 3 min of activation at a reductive current density of 100 mA cm^−2^, suggesting its suitability for practical applications (Figure S10b). AgCl NC achieved an FE_CO_ of 98.4% at 2.75 V, with a maximum *j*_CO_ of 407 mA cm^−2^ at 3.5 V, outperforming AgCl bulk and Ag NP with 281 and 226 mA cm–^2^, respectively. This performance trend indicated that the beneficial effects of the Ag‐coral structure and surface‐retained Cl remained valid in a practical MEA system. Notably, the *j*_CO_ of AgCl bulk and Ag NP reached a maximum at 3.25 V, followed by saturation or a slight decline at 3.5 V, whereas that of AgCl NC increased continuously up to 3.5 V. This suggests that its active sites were not fully utilized under the initial operating conditions.

Based on this observation, we hypothesized that the CO_2_RR capability of the catalyst was not fully exploited and that more intense reaction conditions may lead to improvements. Accordingly, additional tests were conducted at an elevated temperature (60°C) and a higher electrolyte concentration of 0.5 M KHCO_3_ to enhance the reaction rate, ionic conductivity, and stability (Figure 3c; Figures S25 and S26). Under these conditions, AgCl NC exhibited higher *j*_CO_ values across all applied voltages, reaching 761 mA cm^−2^ at 3.5 V and demonstrating outstanding CO productivity comparable to benchmark Ag‐based catalysts (Figure S26 and Table S2). These results indicate the practical viability and robustness of AgCl NC under industrially relevant operating conditions. The long‐term stability of AgCl NC was evaluated in an MEA cell at 3.0 V for 200 h, during which the catalyst maintained stable operation with FE_CO_ consistently above 95% (Figure 3d). Occasional decreases in FE_CO_ observed every 20 to 30 h, attributed to salt precipitation, were fully recovered by flushing the backside of the electrode with deionized water through the CO_2_ flow path. The formation and subsequent removal of potassium‐derived salt precipitates were confirmed by scanning electron microscopy (SEM) and energy‐dispersive X‐ray spectroscopy (EDS) analyses of the cathode GDL before and after flushing (Figure S27). Post‐reaction SEM and XPS analyses confirmed that the catalyst morphology was largely preserved and that Cl species remained on the surface (Figure 3e). The Cl/Ag atomic ratio decreased from 0.95 in the pristine AgCl NC to 0.06 after activation and remained measurable at 0.04 after 200 h of electrolysis. These results demonstrate the durability and practical applicability of AgCl NC under MEA conditions and provide the basis for subsequent catalyst‐to‐system TEA.

**FIGURE 4:**
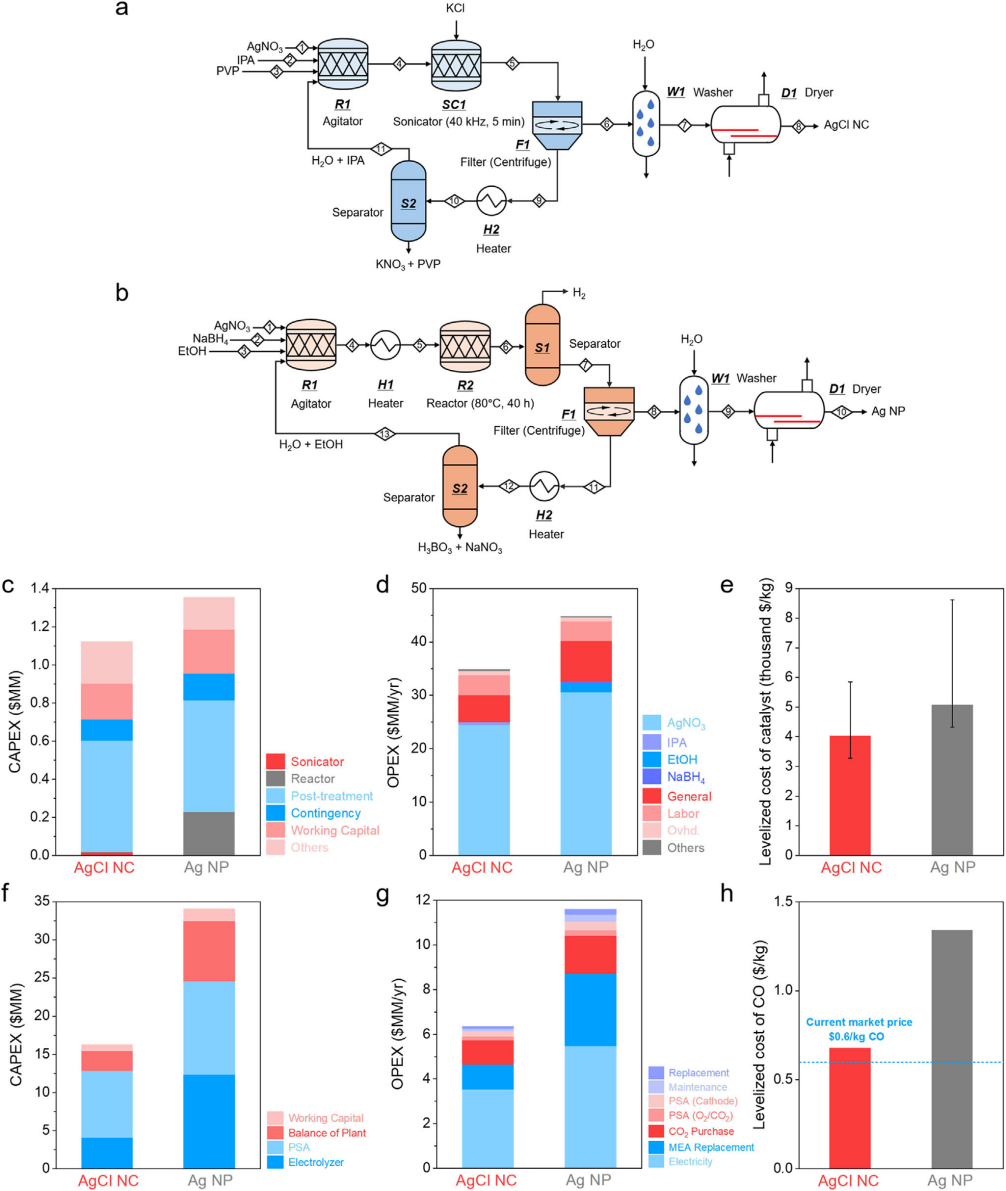
Process flow diagram for (a) AgCl NC and (b) Ag NP production. (c) Capital and (d) operating expenditures for AgCl NC and Ag NP production. (e) Levelized costs of Ag NP and AgCl NC production with error bars indicating the maximum and minimum outcomes based on GSA. (f) Capital and (g) operating expenditures of CO production using AgCl NC and Ag NP. (h) Levelized costs of CO production using AgCl NC and Ag NP, with a base case CO cost of $0.6 kg^−1^.

Catalyst performance as well as the efficiency and scalability of sonochemical synthesis processes critically influence the overall cost and viability of CO_2_RR systems. To address these factors, we developed a high‐yielding, rapid synthetic process for AgCl NC that is suitable for large‐scale production. The AgCl NC production process was established through experimental optimization and served as the base case for the TEA, while a conventional thermal Ag NP synthesis (80°C for 40 h, representative of previously reported routes demonstrated to be compatible with large‐scale production) was compared as a benchmark (Figure 4a,b; Note S4) [41]. Both processes were a scaled to the same 10 t yr^−1^ production capacity and compared. The Ag NP route requires approximately 14.9 t of AgNO_3_ yr^−1^ and involves prolonged thermal treatment followed by post‐treatment. In contrast, the AgCl NC process consumes less AgNO_3_ (≈11.9 t yr^−1^) owing to stoichiometric incorporation of chloride and achieves rapid synthesis (i.e., within 5 min under 20 kHz sonication), effectively replacing the time and energy intensive thermal reaction. The process shares the same downstream operations as Ag NP synthesis except for hydrogen removal. To reduce solvent consumption and operating costs, ethanol and water used are recovered through downstream separation units and recycled back to the reactor.

The total capital expenditure (CAPEX) and operating expenditure (OPEX) were estimated at $1.36 × 10^6^ yr^−1^ and $44.8 × 10^6^ yr^−1^ for Ag NP, and $1.12 × 10^6^ yr^−1^ and $35.0 × 10^6^ yr^−1^ for AgCl NC, respectively (Figure 4c, d; Tables S3 and S4). Most of the CAPEX for both systems originated from post‐treatment equipment. However, the Ag NP process incurred higher capital costs due to the thermal reactor; elimination of this unit in the sonochemical route led to an approximately 90% reduction in the reaction unit cost. Material costs, dominated by AgNO_3_, comprised most of the OPEX. The lower Ag precursor requirement and shorter synthesis time of AgCl NC reduced reagent and energy consumption. These advantages were reflected in the levelized production cost of $4034 kg^−1^ for AgCl NC compared with $5071 kg^−1^ for Ag NP (Figure 4e). Based on a selling price of $4800 kg^−1^, the cash‐flow analysis indicated a breakeven within 4 years and a net present value of $14.7 × 10^6^ over 15 years (Figure S29), underscoring the economic feasibility of the AgCl NC production process [42]. Additional analyses of catalyst production costs, including sensitivity analysis and life cycle analysis (Figures S30–S35 and Note S5).

Building on the advantages in catalyst production cost and performance, we extended the TEA to a 3.5 t day^−1^ CO production process (Figures S36 and S37, Note S6 and Table S5) [43]. Incorporating AgCl NC reduced the required electrolyzer area owing to higher *j*_CO_, thereby decreasing the system CAPEX (Figure 4f). As electricity accounted for the largest share of OPEX, the higher CO productivity and lower replacement demands of AgCl NC reduced the operating cost by nearly 45% (Figure 4g). As a result, the AgCl NC system requires only 47.7% of the total CAPEX and 54.8% of the OPEX relative to the Ag NP system, cutting the levelized CO production cost from $1.34 to $0.68 kg^−1^ (Figure 4h).

## 3 Conclusions

This study presents a catalyst design strategy integrating scalable synthesis, catalytic performance, and economic viability. We developed a rapid and scalable sonochemical route for AgCl NC, enabling gram‐scale production within 5 min. The catalyst achieved outstanding CO_2_RR performance, exhibiting *j*_CO_ >750 mA cm^−2^ and stable operation over 200 h in the MEA configuration. These features arise from the formation of an Ag‐coral structure and PVP‐stabilized surface Cl species that promote CO formation. The rational synthesis strategy effectively reduced synthesis time, process cost, and raw material utilization, translating these efficiencies into substantial cost benefits, achieving ≈20% lower catalyst production cost and ≈50% lower levelized CO production cost than conventional commercial catalyst (Ag NP). This demonstrates that optimizing synthetic protocols can yield synergistic improvements in both performance and economic feasibility. By quantifying the economic impact of rational synthesis development, this work moves beyond performance‐focused TEA approaches and proposes a broader framework for CO_2_RR catalyst design that emphasizes manufacturability and system integration. AgCl NC exemplifies how catalyst design can align with industrial practicability, providing a blueprint for next‐generation CO_2_RR catalysts capable of practical implementation.

## Conflicts of Interest

The authors declare no conflicts of interest.

## Supporting information




**Supporting File 1**: smll74602‐sup‐0001‐SuppMat.pdf.

**Supporting File 2**: smll74602‐sup‐0002‐VideoS1.mp4.

## Data Availability

The data that support the findings of this study are available from the corresponding author upon reasonable request.
