# Supplementary material for: Techno‐Economically Viable, Rapid, and Scalable Sonochemical Synthesis of AgCl Nanocubes for CO2 Electroreduction
Source: Small. 2026 Jul 16;22(51):e74602. doi: 10.1002/smll.74602 (PMC13568835; doi:10.1002/smll.74602)
Supplement: Supplementary file 1 [file SMLL-22-e74602-s001.pdf]

Supporting Information for

Techno-economically Viable, Rapid and Scalable Sonochemical Synthesis of AgCl Nanocubes for CO_2 Electroreduction

Younghyun Chae,[1,2,‡] Hyunyoung Kim,[1,3,‡] Dongjin Kim,[1,4] Woong Choi,[5,6] Hyunwook Kim,[1] Donggu Han,[1] Dong Ki Lee,[1,2,7,8] Wangyun Won,[3] Ung Lee,[1,2,7,*] and Da Hye Won[1,2,*]

[1]Clean Energy Research Center, Korea Institute of Science and Technology, Seoul 02792, Republic of Korea

[2]Division of Energy and Environmental Technology, KIST School, Korea National University of Science and Technology (UST), Seoul 02792, Republic of Korea

[3]Department of Chemical and Biological Engineering, Korea University, Seoul 02841, Republic of Korea

[4]Department of Materials Science and Engineering, Korea University, Seoul 02841, Republic of Korea

[5]Department of Energy Engineering, Gyeongsang National University, Jinju 52725, Republic of Korea

[6]Department of Energy System Engineering, Gyeongsang National University, Jinju 52725, Republic of Korea

[7]Graduate School of Energy and Environment (Green School), Korea University, Seoul 02841, Republic of Korea

[8]Department of Chemical and Biomolecular Engineering, Yonsei-KIST Convergence Research Institute, Yonsei University, Seoul 03722, Republic of Korea

[‡]These authors contributed equally to this work.

*Corresponding author

Da Hye Won − E-mail: dahye0803@kist.re.kr

Ung Lee – E-mail: ulee@kist.re.kr

Table of contents

Experimental Section

Catalyst Preparation and Characterization

Electrochemical Measurements and Analysis

TEA modeling and Calculations

Supporting Figures, Notes and Tables

Figure S1-37

Note S1-6

Table S1-5

References

Experimental Section

Catalyst Preparation and Characterization

Preparation of Ag-based catalysts

(1) *Synthesis of AgCl NC.* For the synthesis of AgCl NC, 0.05 M $AgNO_3$ (99.0%, Sigma Aldrich), 0.5 M PVP (MW = 55,000, Sigma-Aldrich, prepared based on the molecular weight of the monomer), and 0.1 M KCl (99.5%, Duksan) solutions were prepared using a 1:1 (v/v) mixture of deionized water and isopropyl alcohol as the solvent. Initially, 80 mL $AgNO_3$ solution was mixed with 40 mL PVP solution, and the resulting mixture was sonicated using an ultrasonicator (VCX750 Vibra-Cell, Sonics & Materials, Inc.). The ultrasonication was performed at a fixed frequency of 20 kHz and a constant amplitude setting of 80%. During continuous ultrasonication, 40 mL of the KCl solution was added to the mixture. After 5 min, ultrasonication was stopped to terminate the reaction. The reaction mixture was washed several times with acetone (99.5%, Duksan) and ethanol, and the resulting product was redispersed in 10 mL of ethanol for subsequent use. For large-scale synthesis, the same procedure was followed using proportionally scaled amounts of each component, with the total reaction volume increased up to 1,000 mL. To provide sufficient sonication for each reaction volume and maintain uniform morphology, the probe-tip size was selected according to the reaction volume. A 19 mm probe tip was used for volumes of 50-500 mL, whereas a 25 mm probe tip was used for volumes above 500 mL. Using this volume-dependent probe selection while maintaining the amplitude setting at 80% enabled reproducible gram-scale synthesis of AgCl NC.

(2) *AgCl bulk and Ag NP.* For the synthesis of AgCl bulk, 120 mL of 0.025 M $AgNO_3$ and 40 mL of 0.1 M KCl were mixed, without the addition of the PVP solution. Commercial Ag NP (< 100 nm, Alfa Aesar) was purchased and used without further modification.

(3) *Photoreduced AgCl NC.* The photoreduction of AgCl was performed using a solar simulator (Sun 2000, Abet Technologies). The prepared AgCl electrode was exposed to 1 sun for 10 h. The exposure was performed at a distance corresponding to standard sunlight (100 mW cm^{-2}), as measured by a photometer (ILT1400, International Light Technologies).

Physical characterization

The physical characteristics of the catalysts and electrodes were examined using SEM (Inspect F-50, FEI) at 10 kV, TEM (F20 G2, TECNAI) at 200 kV, XRD (D8 ADVANCE LynxEye, Bruker), XPS (Nexsa, Thermo Fisher Scientific), and Raman spectroscopy (inVia Raman

Microscope, Renishaw). TEM images were processed using Gatan Microscopy Suite. The XRD scan rate was 0.05° s^{-1} and the data were processed using Jade. XRD peak analysis was conducted with reference to Ag (JCPDS Card No. 87-0720) and AgCl (JCPDS Card No. 85-1355). The 2θ values for Ag (111), (200), (220), and (311) were assigned at 38.20°, 44.40°, 64.60°, and 77.60°, respectively. Similarly, the 2θ values for AgCl (111), (200), (220), (311), and (222) were indexed at 27.83°, 32.24°, 46.24°, 54.83°, and 57.49°, respectively. The carbon signal arising from the substrate was observed at 54.5°. N-chlorosuccinimide (98%, Sigma Aldrich) and N-methyl-N-phenylcarbamoyl chloride (98%, Sigma Aldrich) were used for XPS spectral comparisons. In situ Raman spectroscopic analysis was performed in a custom-built in situ cell using a 532 nm laser source, and calibration was conducted using a Si wafer to reference the Si peak at 520.3 cm^{-1}. A 1 × 1 cm^2 square electrode, prepared using the same catalyst ink and loading as those used for the H-type cell, was employed for the Raman measurements. Reference spectra of the Ag–Ag and Ag–Cl bonds were obtained using Ag NP and AgCl NC electrodes, respectively. The same electrode size was used for all in situ Raman measurements. CO_2 was supplied at a flow rate of 2 mL min^{-1} to simulate reaction conditions while preventing bubble formation on the electrode surface. During in situ measurements, the laser was focused approximately 2 μm beneath the electrode surface to minimize signal attenuation caused by surface wetting and to better capture any internal structural changes occurring during electroreduction.

Electrochemical Measurements

H-type cell setup and operation

(1) *CO_2RR evaluation.* A custom-built H-type cell was configured in a three-electrode setup and included a catholyte chamber and an anolyte chamber, each equipped with separate gas inlets and outlets. The chambers were separated by a Selemion AMV-N anion exchange membrane (AEM, 130 μm, Asahi Glass) that had been activated in deionized water for more than 24 h. In this configuration, Ag-based catalysts were used for the working electrode, platinum foil (thickness 0.2 mm, WizMac) as the counter electrode, and Ag/AgCl (3 M KCl, RE-5B, BASi Research Products) as the reference electrode. The working electrode was prepared by dispersing 20 mg of Ag or AgCl catalyst and 2 mg of grained PTFE resin (Nanoshel) in 2 mL of ethanol, followed by 30 min of sonication to obtain a homogeneous catalyst ink. Using a spray gun (Gunpiece GP-2, Fuso Seiki), the ink was airbrushed onto a gas diffusion electrode (GDE, Sigracet 39BB GDL; SGL carbon). Catalyst loading was determined by weighing the GDE before and after spray coating and was fixed at 1.0 mg cm^{-2} for a circular electrode with an active area of 0.5 cm^2. For the CO_2RR, a 0.1 M $KHCO_3$ solution was used as the electrolyte, prepared by dissolving $KHCO_3$ salt (99.95%, Sigma-Aldrich) in deionized water and stirring for over 24 h. Prior to the experiments, the electrolyte was continuously bubbled with CO_2 for at least 2 h. Both the catholyte and anolyte chambers were filled with 28 mL of the prepared electrolyte. Before initiating the CO_2RR, the working electrode was electrochemically reduced by applying a reductive current density of 5 mA cm^{-2} for 60 min. CO_2 was continuously supplied at a flow rate of 20 mL min^{-1} during the activation and reaction. The CO_2 flow rate was controlled using a mass flow controller (El-flow, Bronkhorst). Electrochemical experiments were conducted applying chronoamperometry at fixed potentials using a potentiostat (VSP SP-150e, BioLogic) equipped with a 20 A booster (VMP3B-20, BioLogic). The applied potential was converted to the RHE scale with 85% *iR* compensation according to the equation:

$$E_{\mathrm{RHE}}\ (\mathrm{V}) = E_{\mathrm{Ag/AgCl}} + E^{0}{}_{\mathrm{Ag/AgCl}} + \ 0.059 \times \mathrm{pH} \ + \ 0.85 \times iR_{s}$$

where i and R_S denote current and resistance, respectively, as determined using electrochemical impedance spectroscopy (EIS). The pH of the CO_2-bubbled 0.1 M $KHCO_3$ was 6.82 ± 0.02, as measured by a pH meter (SevenCompact, Mettler Toledo). The resistance of each electrode was measured before and after electrolysis using potentiostatic EIS at −1.1 V vs. Ag/AgCl; the CO_2RR is negligible at this potential.

(2) *ECSA.* The ECSAs of the catalysts were determined based on cyclic voltammetry (CV) measurements at various scan rates (10, 20, 30, 40, and 50 mV s^{-1}) in a 0.05 M K_2SO_4 solution. The potential was cycled between −0.02 and 0.10 V vs. Ag/AgCl, within the non-Faradaic region. The current density differences at 0.04 V vs. Ag/AgCl during the forward and reverse scans were plotted against the scan rates. The slope of the resulting linear trend line, representing the specific capacitance, was used to estimate the ECSA of the catalysts.

<u>MEA cell setup and operation</u>

(1) *CO_2RR evaluation.* A commercial MEA cell (5 cm^2 CO_2 electrolyzer, Dioxide materials) was used for the MEA configuration. The cathode consisted of a GDE prepared using the Ag-based catalysts, a PiperION AEM (20 μm, dry, Versogen) membrane was used, and an IrO_2 (99.99%, Alfa-Aesar) anode was selected as the oxygen evolution reaction catalyst. The components were assembled in a zero-gap, direct-contact configuration. The Ag-based cathode was prepared following the same procedure as that employed for the H-type cell, using a square electrode with an active area of 5 cm^2. The IrO_2 anode was fabricated by sonicating and dispersing 30 mg of IrO_2 in 1 mL of isopropyl alcohol, followed by the addition of 30 mg of 5 wt.% of Nafion ionomer solution (Sigma-Aldrich). The IrO_2 ink was airbrushed onto platinized titanium mesh (thickness 0.004 inch, FuelCellStore) to achieve a loading of 1.0 mg cm^{-2}. Prior to assembly, the membrane was pretreated by soaking in 1 M KOH solution for 24 h and then rinsed thoroughly with deionized water. For CO_2RR evaluation, CO_2 gas was humidified to 100% relative humidity by passing through water maintained at 36 °C in a humidifier and then fed to the cathode at a flow rate of 100 mL min^{-1}. Simultaneously, 50 mL of 0.1 M $KHCO_3$ (or 0.5 M $KHCO_3$) solution was supplied to the anode using a peristaltic pump (EMP-600A, EMS-Tech) at 20 mL min^{-1}. The AgCl-based electrodes were briefly activated in the MEA cell by applying a reductive current density of 100 mA cm^{-2} for 3 min. In the experiments involving a two-electrode system MEA cell, various cell voltages were applied using the same potentiostat as that used for the H-type cell, and the corresponding currents were recorded.

(2) *Long-term operation* Long-term stability tests were conducted using the AgCl NC catalyst under the same MEA cell configuration and operating conditions described above for CO_2RR evaluation. The cell was continuously operated at a constant potential of 3.0 V for 200 h. A total of 200 mL of 0.1 M $KHCO_3$ anolyte was replaced once per day with a freshly prepared electrolyte to maintain electrolyte integrity. To prevent salt accumulation and flooding at the cathode GDL, a periodic washing procedure was applied. The washing interval (every 20-30 h) was determined based on the CO selectivity in the GC data, which indicated partial blockage of the cathode surface by accumulated salts. At each washing step, 10 mL of

deionized water (DIW) was introduced together with the CO_2 feed through the cathode inlet line. The DIW rinse was performed while maintaining CO_2 flow to ensure that the liquid passed evenly across the GDL and removed precipitated salts from the electrode backside. The washing water was collected and removed at the water trap connected to GC inlet line. During the washing cycle, the cell operation was not interrupted. The current density and gas product composition were monitored continuously throughout the 200 h operation to evaluate the durability and performance stability of the AgCl NC catalyst.

<u>Product analysis</u>

H_2 and CO generated by the CO_2RR were analyzed using gas chromatography (GC, 8890B GC system, Agilent Technologies) combined with a thermal conductivity detector (TCD) and a flame ionization detector (FID) with a methanizer. GC was conducted using a ShinCarbon ST 100/200 column (1 m, 1 mm/D 1/16 OD silco, Restek) and Ar (99.999%) as the carrier gas. A 1 mL outlet gas sample was collected from the electrochemical cell and analyzed 5 min after applying a specific potential and subsequently at intervals of either 10 or 15 min depending on the GC method employed. Based on the GC analysis data and the measured gas flow rate, the FEs of the products were calculated as the ratio of the partial current for each product to the total current, using the following equation:

$$\mathrm{FE}_{\mathrm{product}}\ (\%) = \frac{i_{\mathrm{product}}}{i_{\mathrm{total}}} \times 100 = \frac{\mathrm{mole}_{\mathrm{product}} \times \mathrm{F} \times \mathrm{N}}{i_{\mathrm{total}}} \times 100$$

where $mole_{product}$ is the actual number of moles per second of the product quantified by GC, F is the Faraday constant (96,485 C mol^{-1}), and N is the number of electrons consumed to produce each product from CO_2. All reductive current densities are expressed without minus signs. Liquid-phase products in the electrolyte were analyzed using ^{1}H nuclear magnetic resonance (NMR, Bruker 400 MHz Ultrashield^{TM}). Liquid-phase products in the electrolyte were analyzed using 1H nuclear magnetic resonance (NMR, Bruker 400 MHz UltrashieldTM). For the NMR sample preparation, 300 μL of the post-reaction electrolyte was mixed with 100 μL of D_2O containing 0.05 wt. % 3-(trimethylsilyl)propionic-2,2,3,3-d_4 acid. To eliminate the intense signal from water, a water-suppressed proton pulse sequence was employed. The quantification of liquid products was based on the integration of characteristic peaks relative to the internal standard. The sensitivity of the NMR analysis was evaluated by estimating the limit of detection (LOD) for formate and methanol. Following the commonly adopted standard convention in analytical chemistry, the LOD was defined using a signal-to-noise (S/N) ratio of 3. The characteristic peaks of 1.66 mM formate and 0.55 mM methanol standard solutions were clearly resolved, with signal amplitudes of approximately 30 and 60, respectively, against

a baseline noise amplitude of approximately 3 (Figure S11). The LODs were calculated using the following equation:

$$\mathrm{LOD} = C_{standard} \times \frac{3}{S/N}$$

where $C_{standard}$ is the concentration of the standard solution and S/N is the signal-to-noise ratio. Applying this criterion, the estimated LODs were 0.50 mM for formate and 0.08 mM for methanol. No liquid products were observed under any of the CO_2RR conditions.

TEA Modelling and Calculations

The CAPEX and OPEX for the two silver catalyst production processes were evaluated using cost estimates based on the market prices of the raw materials. For the AgCl NC synthesis process, the cost assessment specifically included the market price of sonicator probes. By integrating the corresponding data, a comprehensive economic analysis was conducted to compare the financial viability of both production processes. The net present value (NPV) based on yearly cash flow was evaluated to estimate the profitability of the catalyst production processes over a plant life of 15 years. Annual cash flow was calculated by incorporating net earnings (NE) and depreciation charges while accounting for operational costs and taxation. The NE was determined by subtracting the OPEX from the total sales revenue, which was calculated as the product of the selling price and the annual production capacity. Depreciation was considered based on the Modified Accelerated Cost Recovery System (MACRS), and tax was applied only when the profit before depreciation was positive. The discounted cash flow for each year included depreciation, while at the end of the lifespan, working capital was recovered. The cumulative NPV was computed by discounting annual cash flows using the specified interest rate, reflecting the time value of money. Detailed cost parameters and calculations are provided in Tables S3–5 of the Supporting Information.

Supporting Figures, Notes

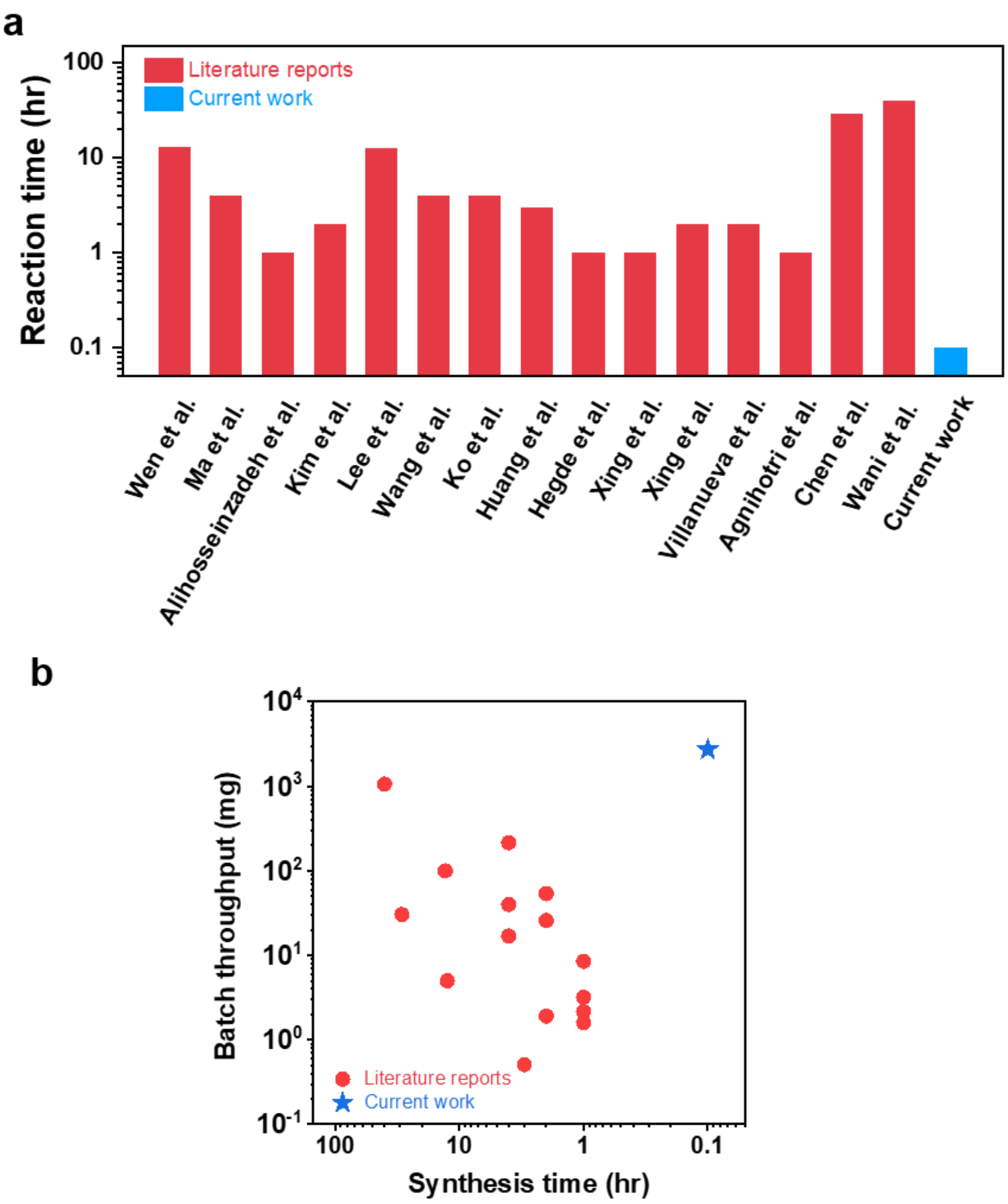

Figure S1. Comparison of synthesis time and yield for Ag nanoparticles synthesis methods reported in the literature. (a) Synthesis time for various reported methods. (b) Reported synthesis time plotted against corresponding yield. The synthesis methods and corresponding data are provided in Table S1.

Note S1. Optimization of AgCl NC synthesis and characterization

To establish reproducible conditions for the rapid sonochemical synthesis of AgCl NC, several synthesis parameters were qualitatively screened, including the $AgNO_3$ concentration, solvent composition, $AgNO_3$/KCl ratio, PVP amount, sonication duration, and reaction temperature. Preliminary trials showed that $AgNO_3$ had limited solubility in the tested solvent systems. At 0.1 M, complete dissolution was not achieved, whereas 0.05 M solutions remained clear and stable in all solvent compositions examined. Accordingly, the precursor concentration was fixed at 0.05 M, and other synthesis parameters were adjusted based on this condition.

When $AgNO_3$ and KCl were directly mixed without additives, precipitation occurred too rapidly, and no stable dispersion was formed. Magnetic stirring during KCl addition resulted in non-uniform particles (Figure S2). In contrast, replacing it with sonication significantly improved the homogeneity of the suspension, and the addition of PVP effectively prevented aggregation (Figure S3). Morphology was strongly influenced by the PVP amount, solvent composition, and Ag^+/Cl^- ratio. Increasing the PVP concentration enhanced particle uniformity and cubic shape definition, whereas pure water as solvent, or excess KCl led to irregular morphologies (Figures S3 and S4). The reaction was also affected by sonication time and temperature. A duration of 5 min yielded reproducible products, and maintaining the reaction in a water bath kept the temperature near 30 °C. Without cooling, the solution temperature rose to about 60 °C, leading to partial aggregation and morphology loss (Figures S5). Under the optimized condition (Ag^+/Cl^- = 1:1, PVP/Ag^+ = 5 equivalents, IPA/DIW = 1:1, 5 min sonication at RT with a water bath), a uniform cubic AgCl NC with an average size of about 60–70 nm was reproducibly obtained. The simplicity and consistency of this optimized process allow for easy implementation at a larger scale. The proposed sonication system is designed for linear scale-up. This can be achieved by parallelizing multiple sonication units according to the required feed volume.

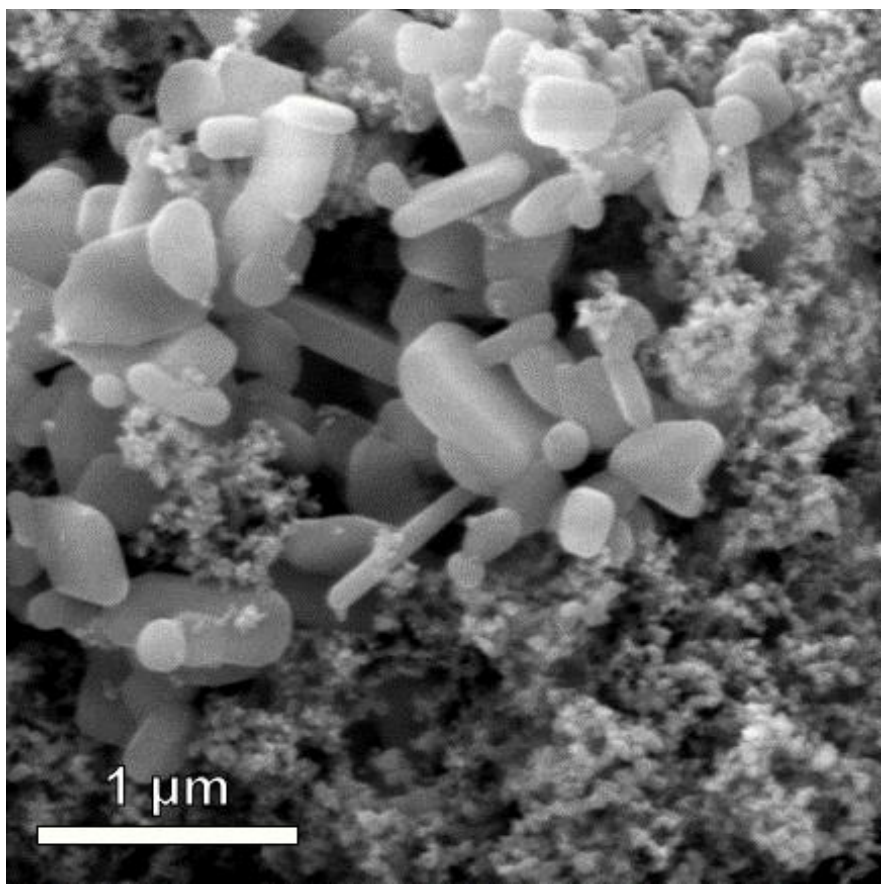

Figure S2. Effect of sonication on AgCl particle formation. SEM images of AgCl particles synthesized under identical conditions to the AgCl NC recipe, except for sonication. AgCl prepared with PVP under magnetic stirring instead of sonication, resulting in irregular and non-uniform particles. These results confirm that sonication is essential for achieving uniform AgCl NC.

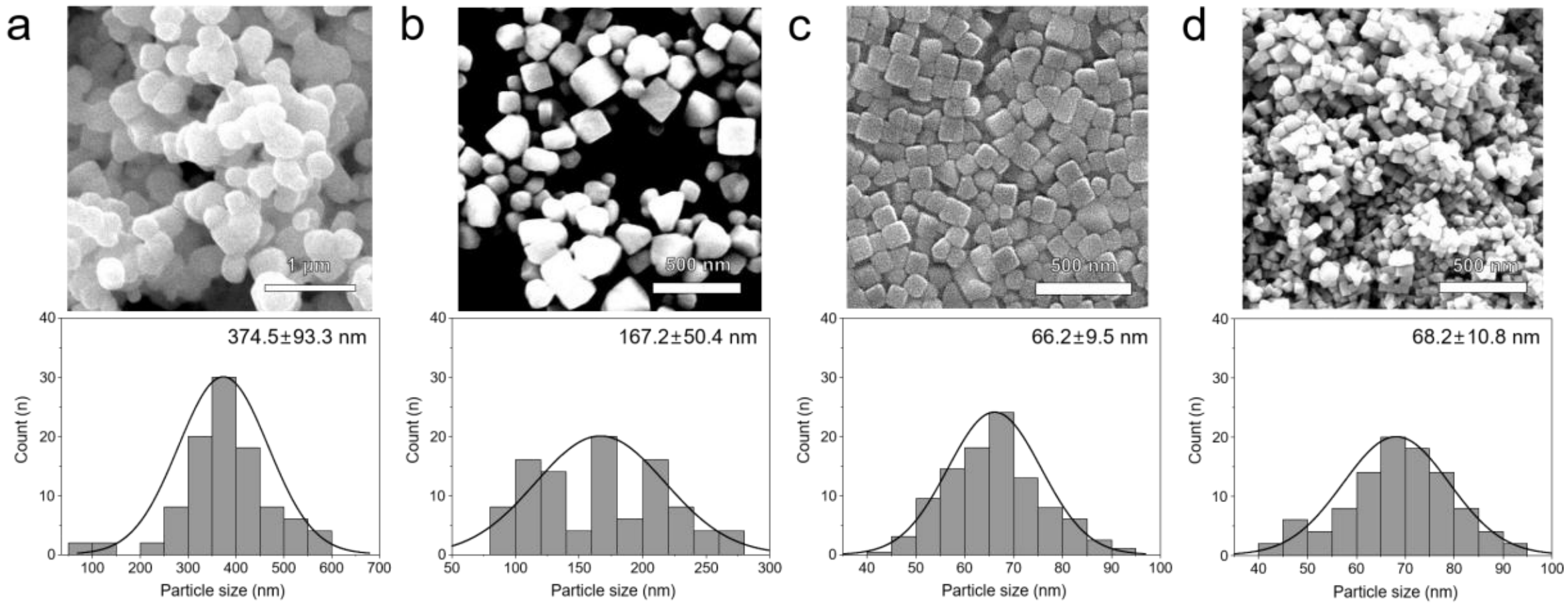

e

PVP (Equiv.)	0	1	5	10
Yield (%)	17.3±8.3	57.1±9.9	79.9±5.0	68.8±5.1

Figure S3. Effect of PVP amount on AgCl particle morphology, particle size, and synthesis yield of AgCl particles. SEM images and corresponding particle-size distribution histograms of AgCl particles synthesized with different PVP equivalents relative to $AgNO_3$: (a) 0 equivalent, (b) 1 equivalent, (c) 5 equivalents, and (d) 10 equivalents. Particle-size distributions were obtained by measuring at least 100 particles for each sample. (e) Table summarizing the synthesis yields of AgCl particles obtained with different PVP equivalents. The synthesis yield was calculated relative to the theoretical AgCl yield assuming complete conversion of $AgNO_3$ to AgCl. The same amount of $AgNO_3$ precursor (680 mg, corresponding to a theoretical AgCl yield of approximately 560 mg) was used in all experiments. The yields were obtained from at least three independent syntheses and are presented as the mean ± standard deviation. (Note: Samples (a) and (c) correspond to the AgCl bulk and AgCl NC samples, respectively)

In the absence of PVP, relatively large AgCl particles with poorly defined morphology were obtained. The addition of 1 equivalent of PVP decreased the particle size, although the resulting particles remained non-uniform. In contrast, the use of 5 equivalents of PVP produced AgCl particles with a well-defined cubic morphology. Increasing the PVP amount to 10 equivalents did not result in any further improvement in particle size or morphology. Therefore, 5 equivalents of PVP was selected as the optimal condition for AgCl NC synthesis.

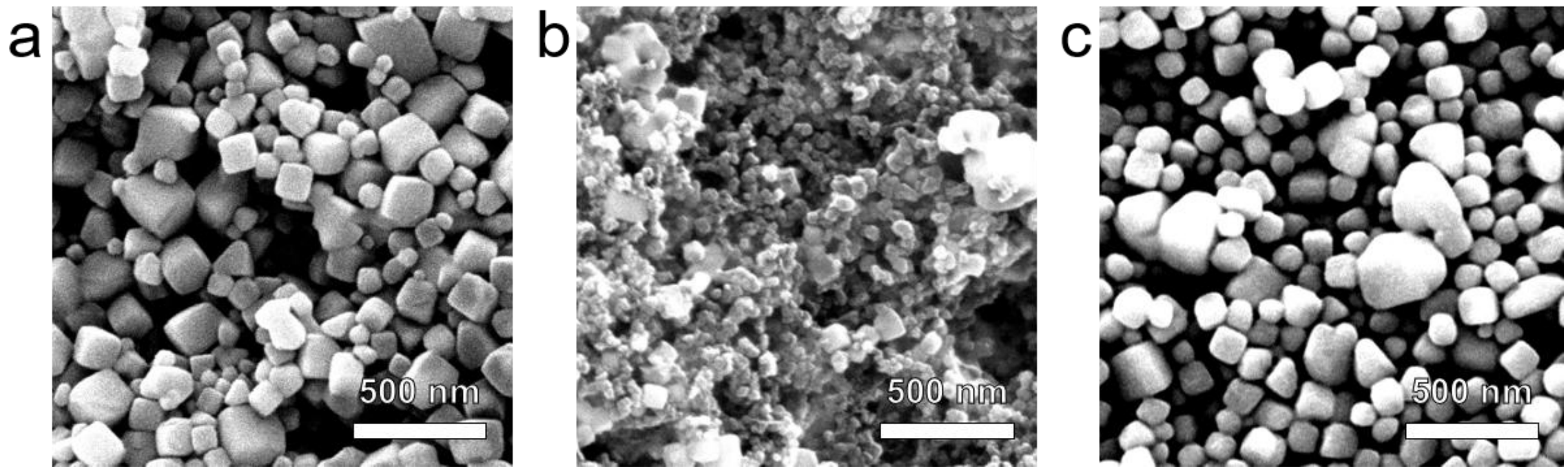

Figure S4. Effect of solvent composition, KCl ratio, and reaction temperature on AgCl particle morphology. The SEM images of AgCl particles synthesized under modified conditions compared with the optimized AgCl NC recipe: (a) DI water as the sole solvent, (b) KCl amount increased to 1.2 equivalents relative to $AgNO_3$, and (c) reaction without a water bath, where the solution temperature rose to approximately 60 °C during sonication. Each condition led to irregular or less-defined morphologies, demonstrating that maintaining an IPA/DIW = 1:1 solvent ratio, KCl = 1 eq, and temperature near 30 °C using water bath is critical for producing uniform AgCl NC.

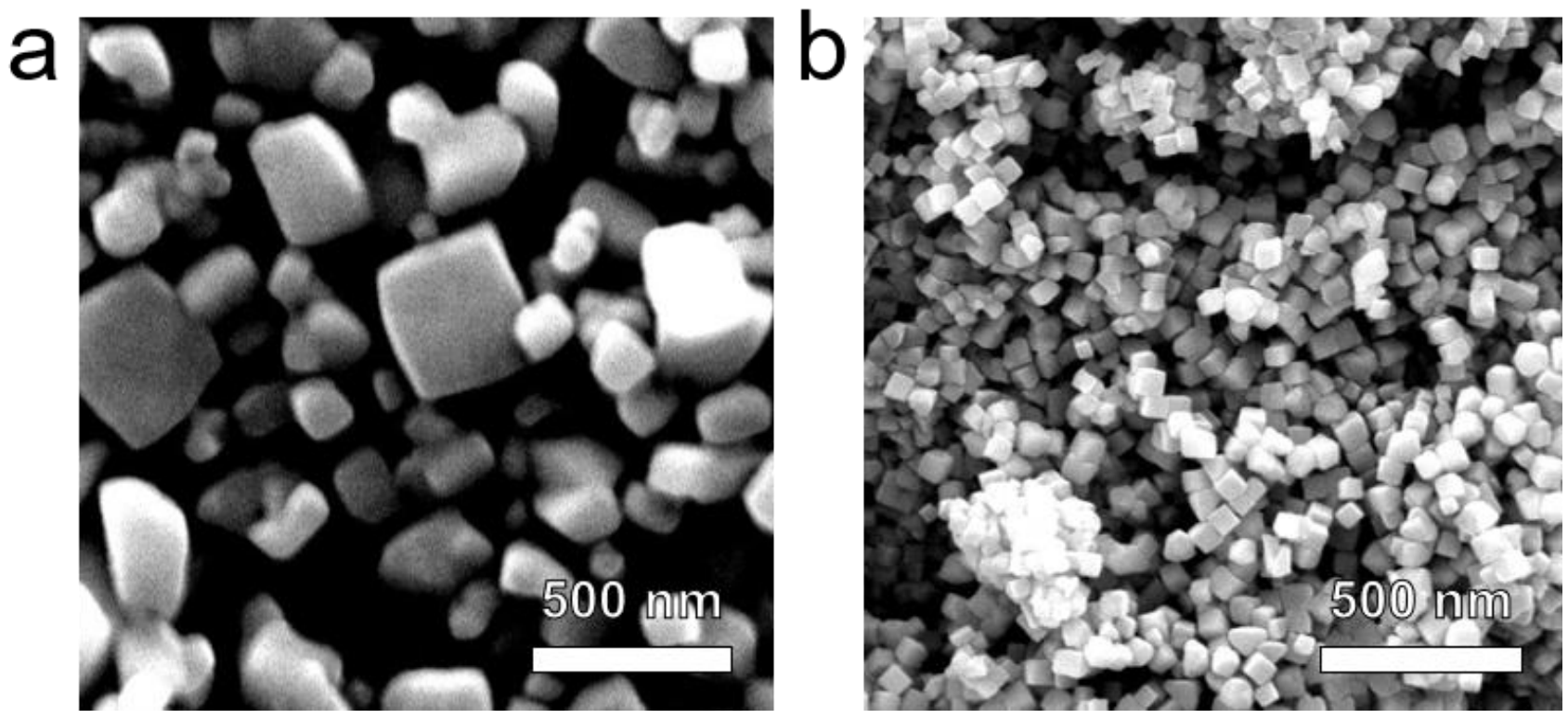

Figure S5. Effect of sonication time on AgCl particle morphology. SEM images of AgCl particles synthesized under identical conditions to the AgCl NC recipe, except for the sonication duration: (a) 3 minutes and (b) 10 minutes. The sample prepared with a 5-minute sonication (AgCl NC, shown in Figure 2) exhibited well-defined cubic morphology. At 3 minutes, the morphology was less defined with partially formed cubes, whereas extending the sonication to 10 minutes did not produce a noticeable difference, confirming 5 minutes as the optimized sonication time.

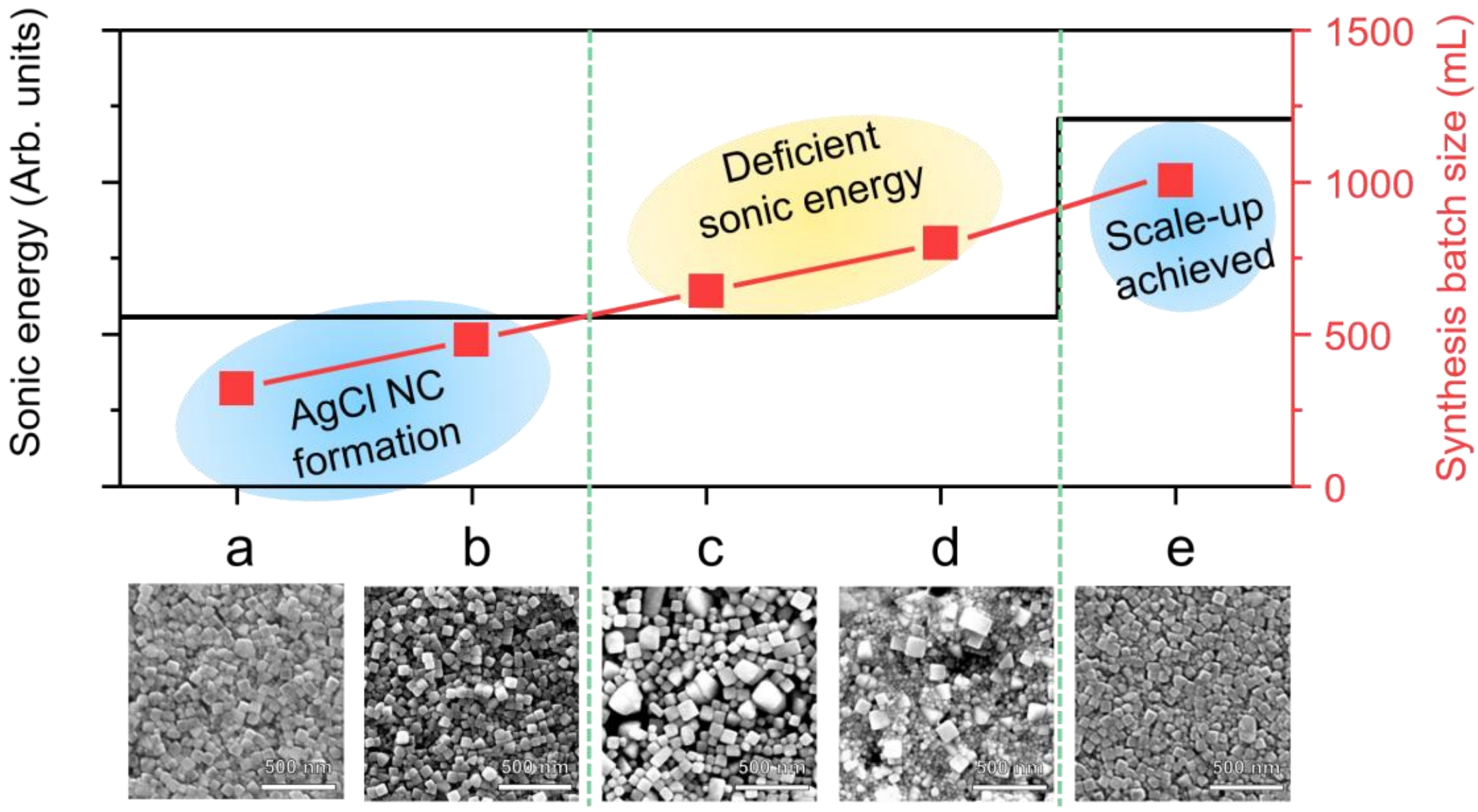

Figure S6. Correlation between synthesis batch size and sonic energy input for AgCl NC. SEM images (a–e) show AgCl NC synthesized at different batch sizes: (a) 320 mL, (b) 480 mL, (c) 640 mL, and (d) 800 mL using a 19 mm probe, and (e) 1000 mL using a 25 mm probe. The schematic graph illustrates the morphological transition observed when scaling up the synthesis with a fixed probe size. AgCl NC was formed up to 480 mL, but further increases in batch size led to irregular morphologies, suggesting a sonic energy limitation (green dashed line). When the probe size was increased to 25 mm at 1000 mL, uniform AgCl NC was synthesized, indicating that sufficient sonic energy input enables scalable synthesis.

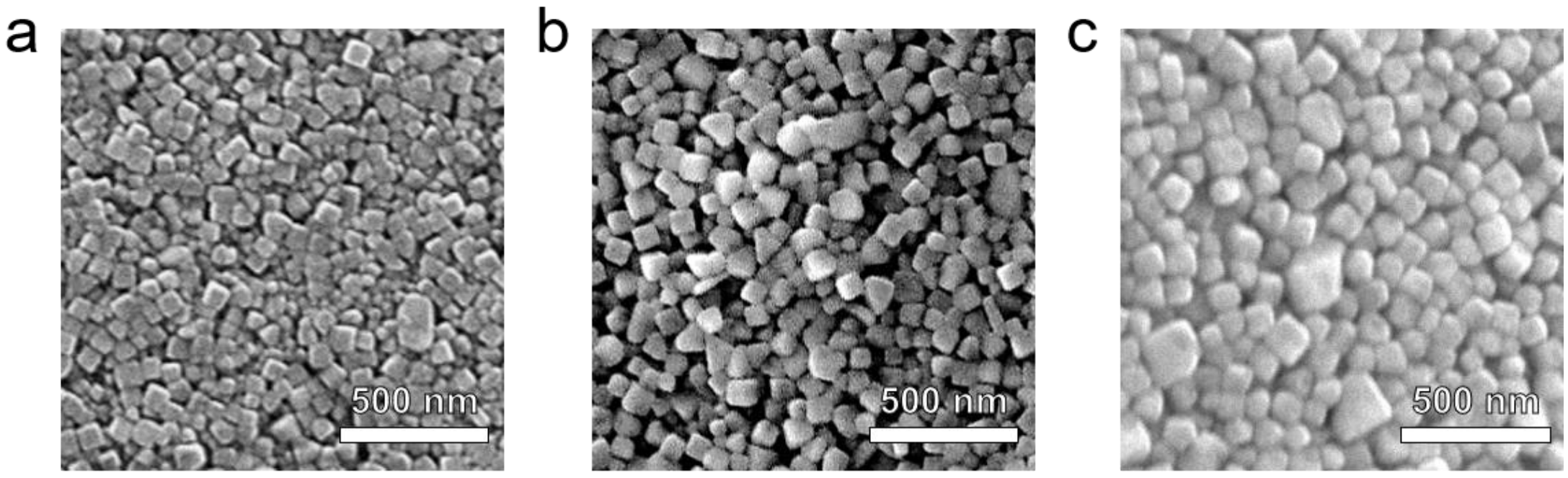

d

Sample	a	b	c
Catalyst amount (mg)	2722	2508	2472
Yield (%)	81.0	73.6	74.6
Average		76.4 ± 3.3%	

Figure S7. Reproducibility of gram-scale AgCl NC synthesis at the 1000 mL scale. (a-c) SEM images show AgCl NC obtained from three independent synthesis batches performed under identical conditions using a 25 mm probe. (d) Summary of the catalyst amount and isolated yield for each batch, along with the overall average yield. All batches exhibited comparable particle sizes and consistent yields, demonstrating good batch-to-batch reproducibility. The average isolated yield was 76.4±3.3%, confirming the reproducibility and scalability of the synthetic method.

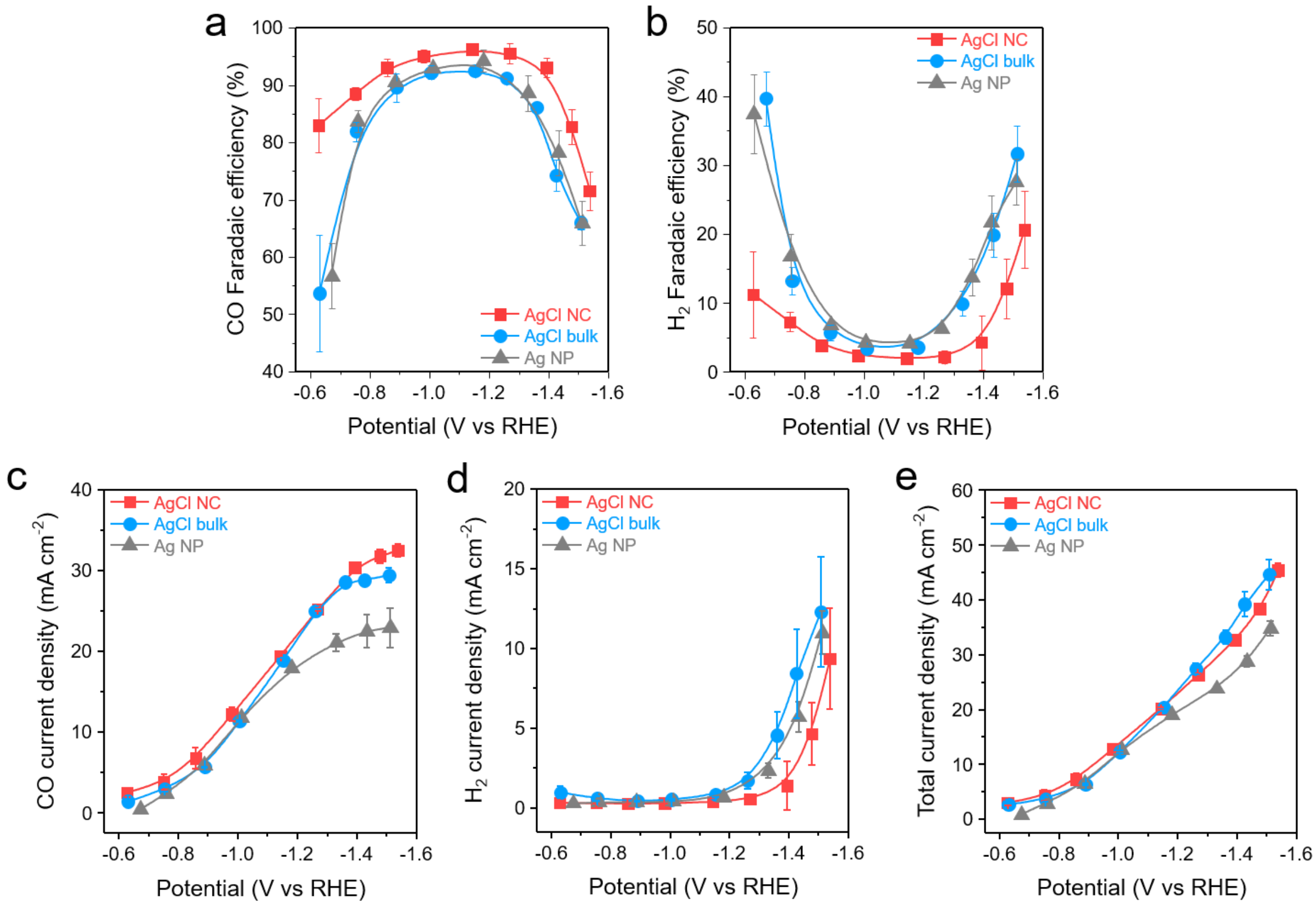

Figure S8. CO_2RR performance of AgCl-based catalysts and Ag NP in an H-type cell. Faradaic efficiencies for (a) CO and (b) H_2. Partial current densities for (c) CO and (d) H_2, and (e) total current densities. All data were collected in CO_2-saturated 0.1 M $KHCO_3$ at potentials ranging from –0.6 to –1.6 V vs. RHE. Error bars represent the standard deviation calculated from at least 3 data points.

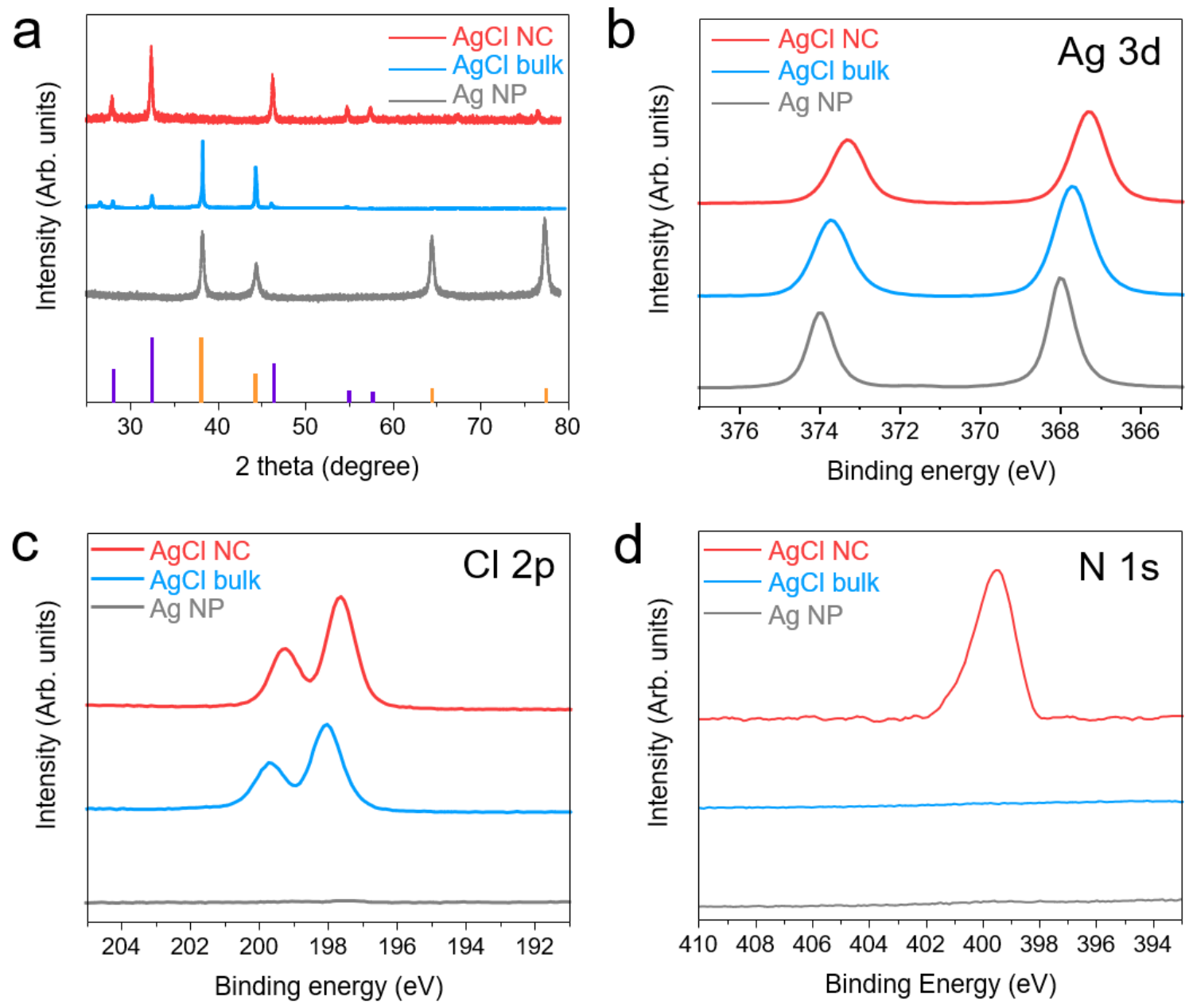

Figure S9. Physical characterizations of AgCl-based particles and Ag NP. (a) XRD patterns. AgCl NC shows characteristic peaks of AgCl, whereas the AgCl bulk exhibits a mixed pattern of AgCl and metallic Ag. The Ag NP displays diffraction peaks corresponding to metallic Ag. XPS spectrum of (b) Ag 3d, (c) Cl 2p and (d) N 1s.

Note S2. Electrochemical activation and structural transformation of AgCl NC

The electrochemical reduction behavior of AgCl NC was investigated to clarify its activation process and morphological evolution during CO_2RR. Time-dependent SEM images recorded in an H-cell revealed that the well-defined cubic morphology of AgCl NC gradually transformed into Ag-coral structure over 45 minutes under a reductive current density of 5 mA cm^{-2}. This gradual morphological change indicates that the reduction of AgCl proceeds progressively through the electrochemical consumption of AgCl.

To compare reduction behaviors among catalysts, post-reaction SEM images of AgCl bulk and Ag NP electrodes obtained under the same H-cell conditions, together with AgCl NC after reduction in an MEA cell, were obtained (Figure S10). AgCl bulk exhibited weaker Ag-coral structure with less porosity, while Ag NP maintained its initial particle-like structure. The MEA-based electrode (Figure S10b) confirmed that this reduction process was completed within a much shorter time under practical operating conditions.

The evolution of CO_2RR performance correlated with this structural transition (Figure S12). AgCl NC showed a characteristic activation period, during which both CO and H_2 FE increased gradually. AgCl bulk required less charge input to complete reduction and exhibited faster activation, while Ag NP displayed stable FE throughout the reaction. XRD patterns after the reaction showed identical metallic Ag peaks for all catalysts, confirming that all were eventually converted to Ag, regardless of the initial phase or morphology (Figure S13), indicating that the intrinsic properties of metallic Ag itself do not account for the observed performance differences.

Cyclic voltammetry at varying scan rates was used to estimate the electrochemical surface area (ECSA, Figure S14). The Ag-coral structure derived from AgCl NC exhibited the largest capacitance, indicating a significantly higher accessible surface area than those derived from AgCl bulk or Ag NP.

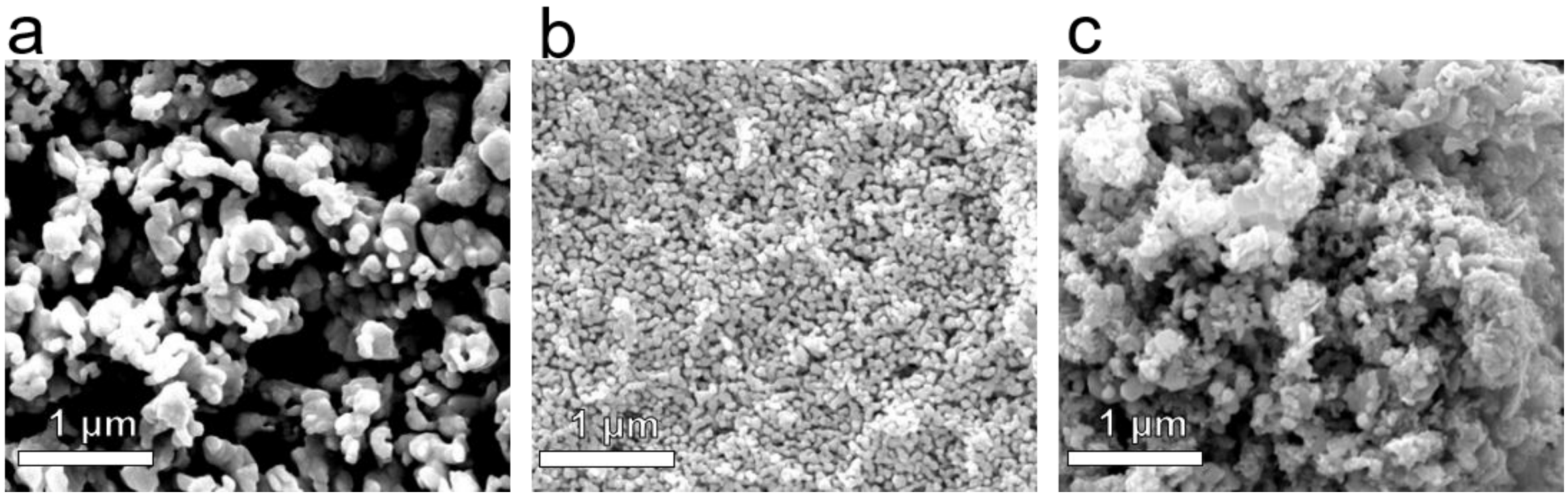

Figure S10. SEM images of electrode surfaces after electroreduction. (a) AgCl bulk electrode after electroreduction at reductive current density of 5 mA cm^{-2} for 15 min. (b) AgCl NC electrode after electroreduction in a MEA cell (3 min, 100 mA cm^{-2}). (c) Surface of Ag NP electrode after same condition for AgCl NC electroreduction (60 min, 5 mA cm^{-2}).

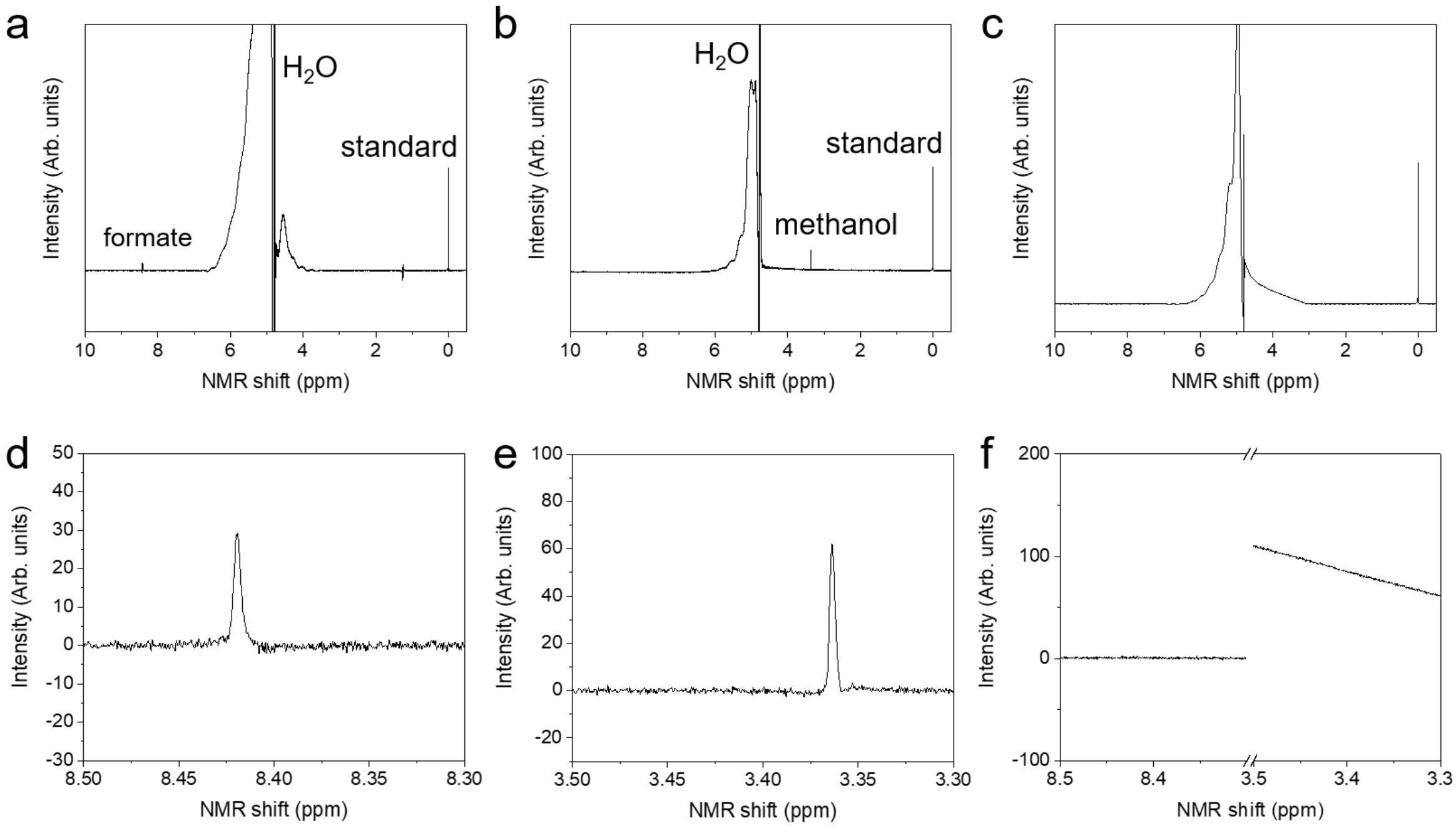

Figure S11. Detection of liquid products in the post-reaction electrolyte by ^{1}H NMR spectroscopy. ^{1}H NMR spectrum of (a) 1.66 mM formate standard solution. (b) 0.55 mM methanol standard solution. (c) Post-reaction electrolyte (after 200-h electrolysis). (d) Magnified formate peak (8.4 ppm) from (a). (e) Magnified methanol peak (3.4 ppm) from (b). (f) Corresponding regions in the post-reaction electrolyte (c), confirming the absence of detectable liquid products. All spectra were calibrated to the internal standard (0.05 wt.% 3-(trimethylsilyl)propionic-2,2,3,3-d_4 acid) at 0 ppm.

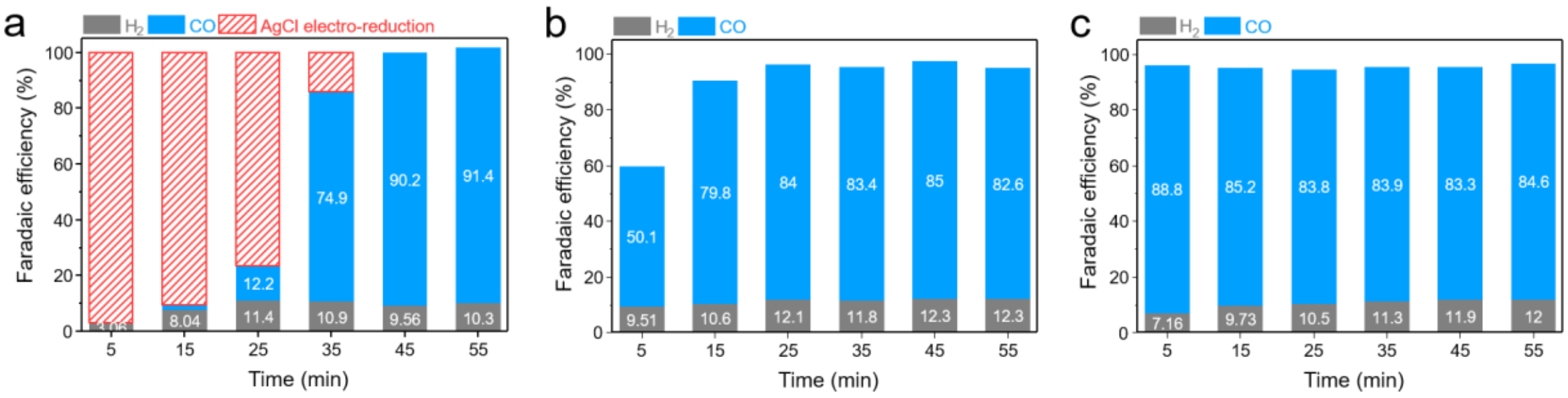

Figure S12. CO_2RR selectivity of AgCl bulk and Ag NP under AgCl NC electroreduction conditions. CO_2RR and HER FEs of (a) AgCl NC, (b) AgCl bulk, and (c) Ag NP. AgCl NC maintained a total Faradaic efficiency below 100% until approximately 45 minutes, indicating that a certain charge input was required to complete the electroreduction of AgCl. AgCl bulk reached approximately 100% within 15 minutes. Ag NP catalyst consistently exhibited nearly 100% FE for CO and H_2.

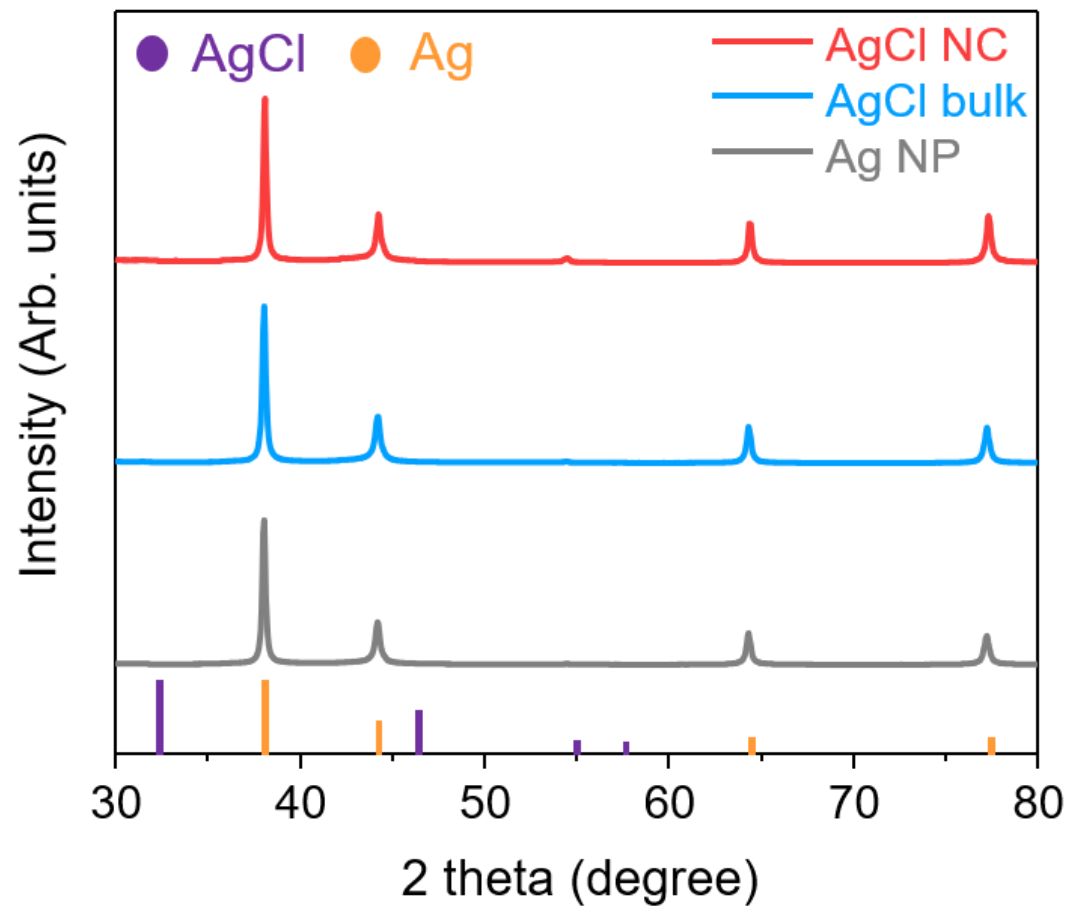

Figure S13. XRD patterns of the AgCl-based catalyst and the Ag NP electrode after the reaction. All the electrodes displayed diffraction peaks corresponding to metallic Ag.

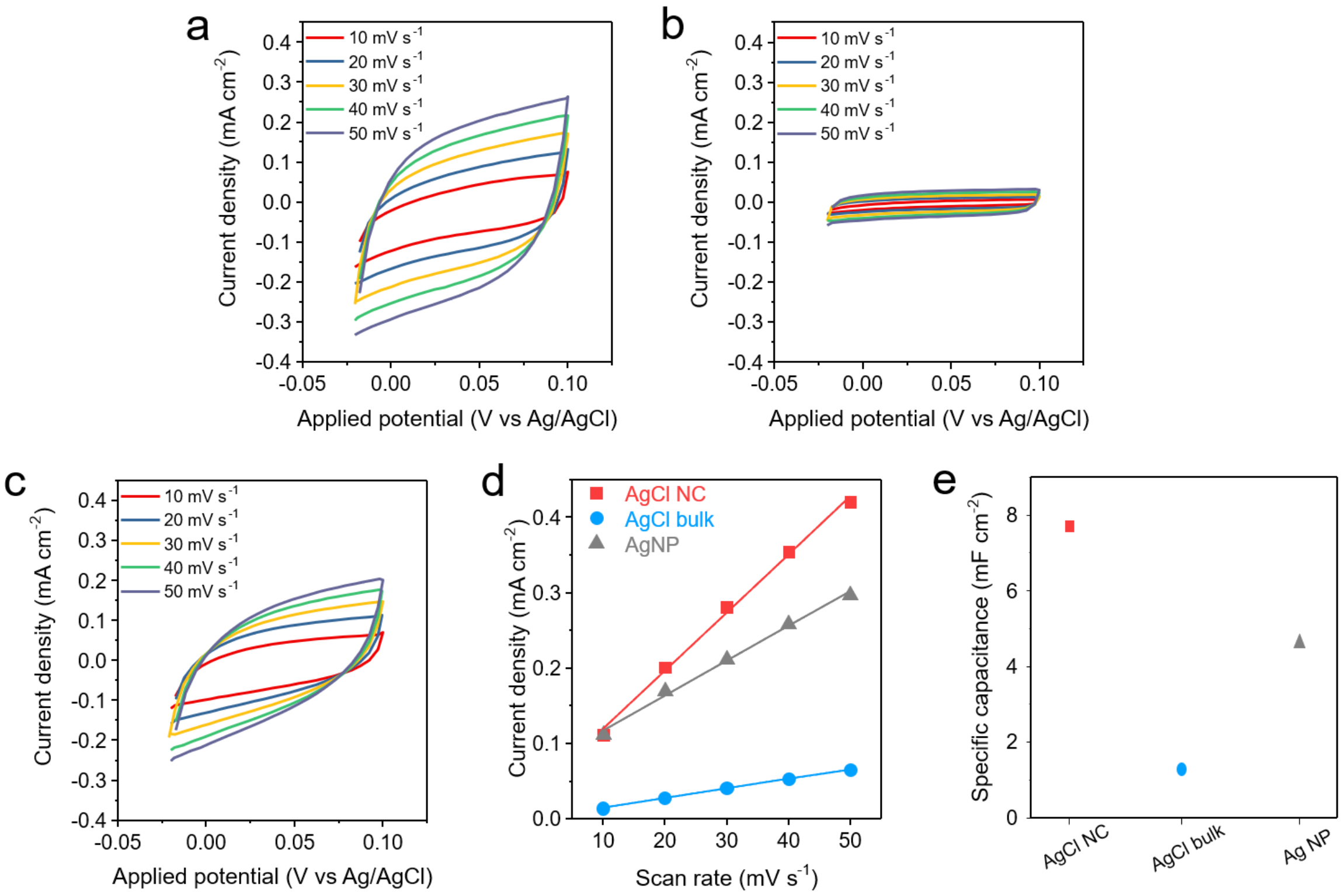

Figure S14. Electrochemical active surface area (ECSA) analysis of AgCl-based samples and Ag NP. Cyclic voltammograms recorded at various scan rates (10-50 mV s^{-1}) in 0.05 M K_2SO_4 solution for (a) AgCl NC, (b) AgCl bulk, and (c) Ag NP. (d) Plot of the difference in current density between anodic and cathodic sweeps at the middle potential versus scan rate with linear fitting. (e) Specific capacitance values estimated from the slope of the fitted lines.

Note S3. Photo-sensitivity of AgCl NC

The photo-sensitivity of AgCl NC was investigated to evaluate its structural stability under light exposure. As AgCl is inherently photosensitive, it can undergo photoreduction under illumination or electron beam exposure. Sequential TEM images captured at 2-second intervals revealed that AgCl NC gradually decomposed under the electron beam, indicating the occurrence of beam-induced reduction during imaging (Figure S15).

To assess its photo-stability under practical conditions, AgCl NC electrodes were intentionally exposed to ambient light prior to electrochemical testing. The color of the electrode changed noticeably after light exposure, suggesting partial photoreduction of surface AgCl (Figure S16a). However, SEM observation showed that the overall morphology of AgCl NC remained largely unchanged (Figure S16b), indicating limited structural degradation compared with bare AgCl materials.

The effect of this photoreduction on electrochemical performance in an H-cell was further evaluated (Figure S17). The light-exposed AgCl NC electrode exhibited nearly identical CO_2RR selectivity, current density, and ECSA compared with the pristine AgCl NC electrode. Both samples showed similar CO and H_2 Faradaic efficiencies and comparable partial current densities, while cyclic voltammetry confirmed that the capacitance remained unchanged. These results suggest that, despite the intrinsic photosensitivity of AgCl, the nanocube structure effectively mitigates photo-induced degradation and maintains its morphological and electrochemical integrity under moderate light exposure, demonstrating sufficient chemical robustness for practical and scalable application.

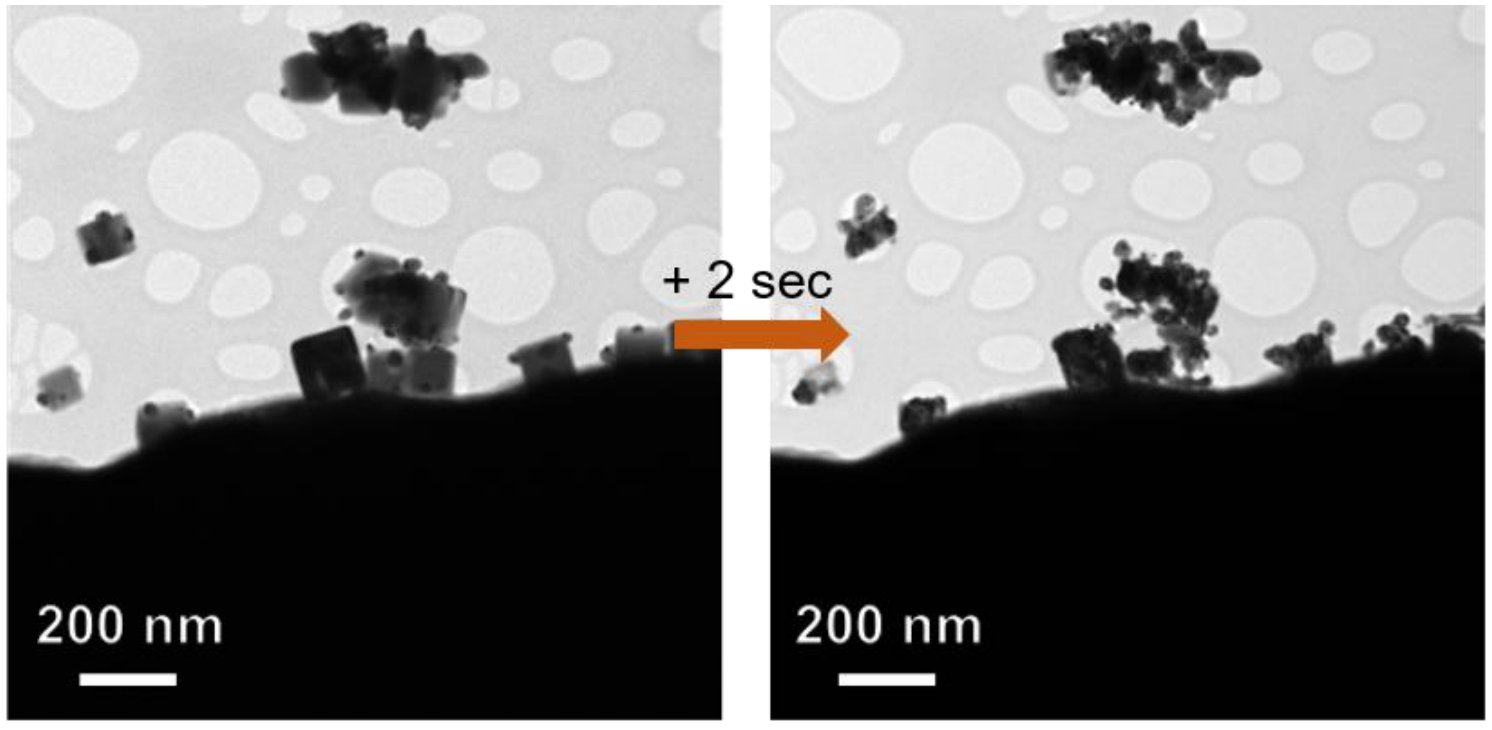

Figure S15. TEM images of AgCl NC. Sequential TEM images captured at a 2-second interval from the same region of interest, showing electron beam-induced degradation of the AgCl NC.

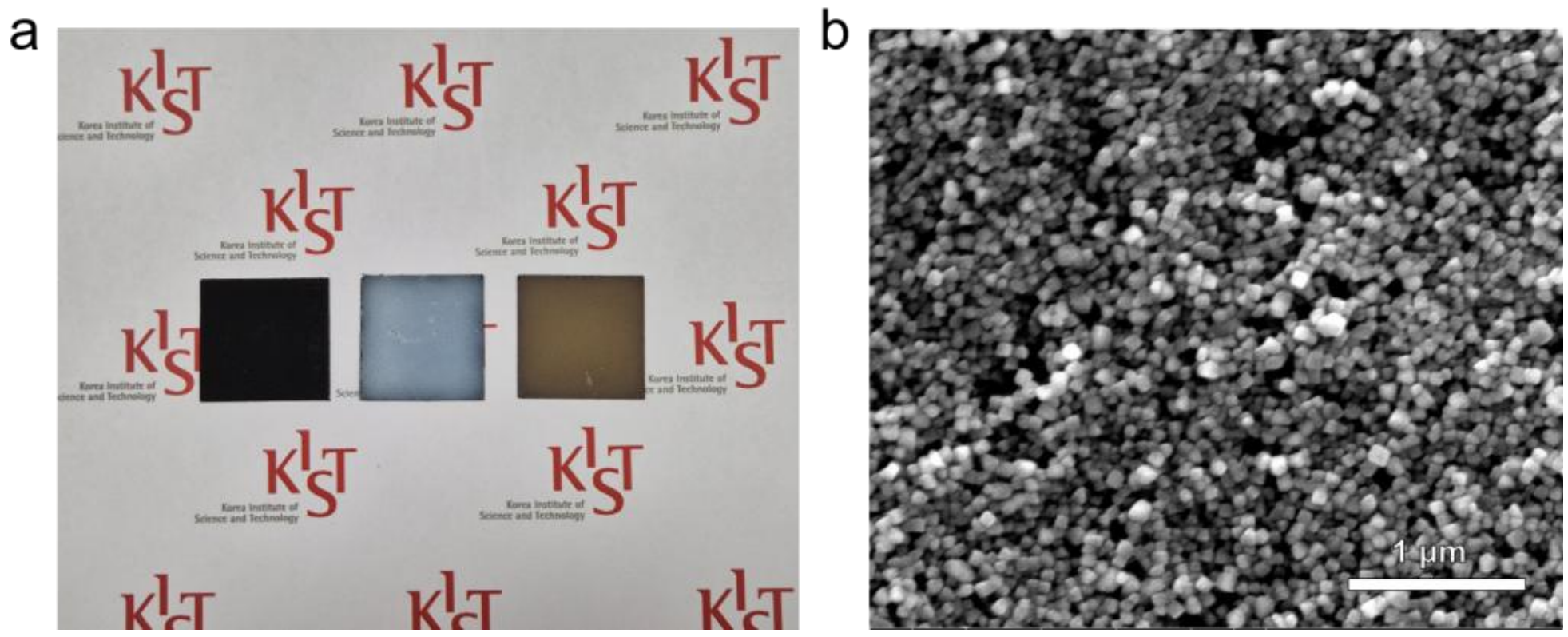

Figure S16. Comparison of AgCl NC electrodes before and after photoreduction. (a) Photographs of pristine carbon paper, AgCl NC electrode, and photoreduced AgCl NC electrode. (b) SEM image of the AgCl NC electrode after photoreduction. Although the electrode exhibited a noticeable color change upon photoreduction, the morphology of the AgCl NC particles remained largely unchanged.

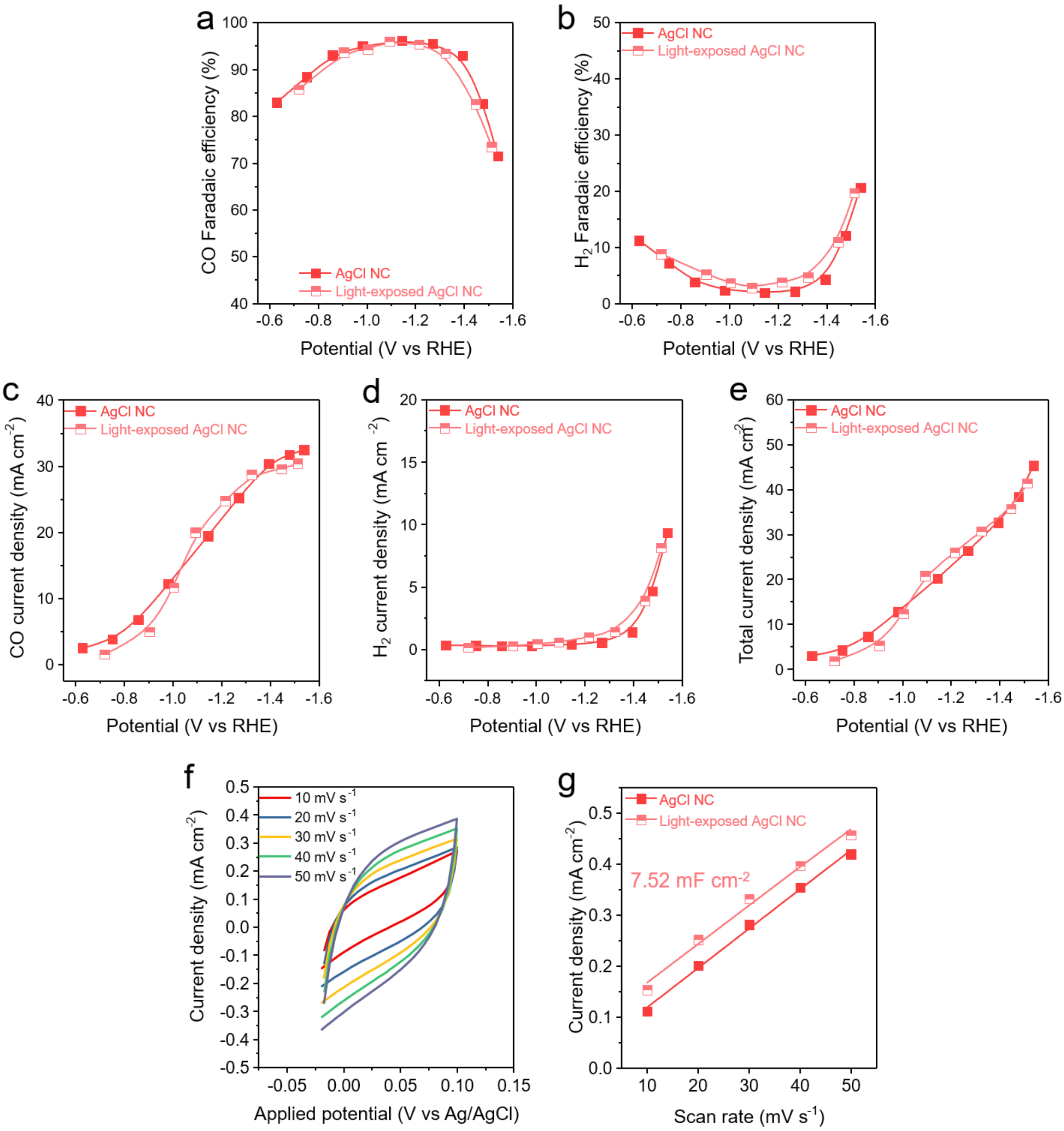

Figure S17. CO_2RR performance and ECSA analysis of light-exposed AgCl NC electrode compared with pristine AgCl NC electrode. Faradaic efficiencies for (a) CO and (b) H_2. Partial current densities for (c) CO and (d) H_2, and (e) total current density. (f) CV of the light-exposed AgCl NC electrode recorded at various scan rates (10–50 mV s^{-1}) in 0.05 M K_2SO_4. (g) Plot of the current difference between anodic and cathodic sweeps at the middle potential versus scan rate with linear fitting. All CO_2RR data were collected in CO_2-saturated 0.1 M $KHCO_3$ at potentials ranging from –0.6 to –1.6 V vs. RHE, using an H-type cell.

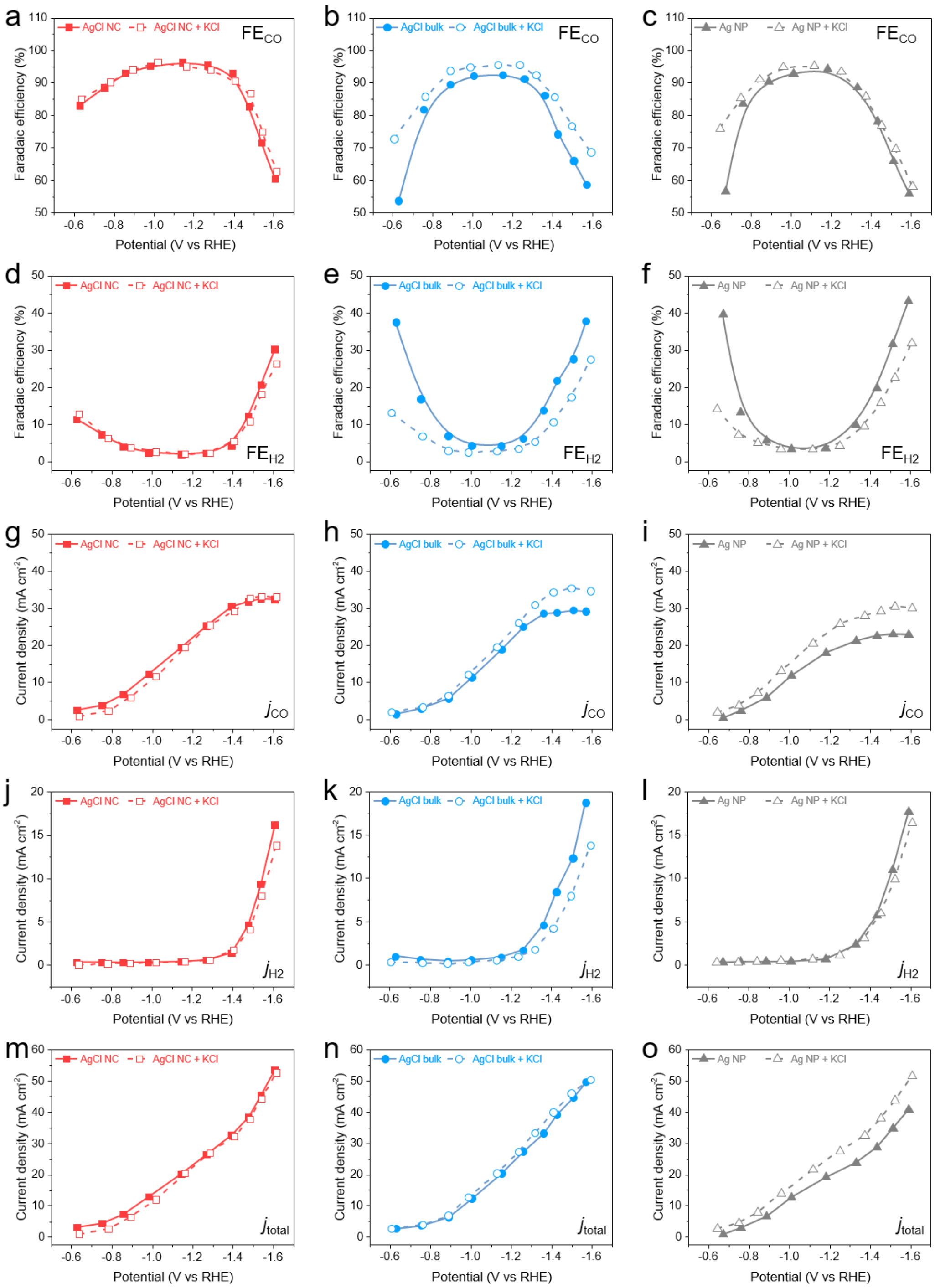

Figure S18. CO_2RR performance of (a, d, g, j, and m) AgCl NC, (b, e, h, k, and n) AgCl bulk, and (c, f, i, l, and o) Ag NP in both Cl-free (0.1 M $KHCO_3$) and Cl-containing (0.09 M $KHCO_3$ + 0.01 M KCl) electrolytes using an H-type cell. Faradaic efficiencies for (a-c) CO and (d-f) H_2, partial current densities for (g-i) CO, (j-l) H_2, and (m-o) total current densities. All data were collected in CO_2-saturated electrolytes at potentials ranging from –0.6 to –1.6 V vs. RHE.

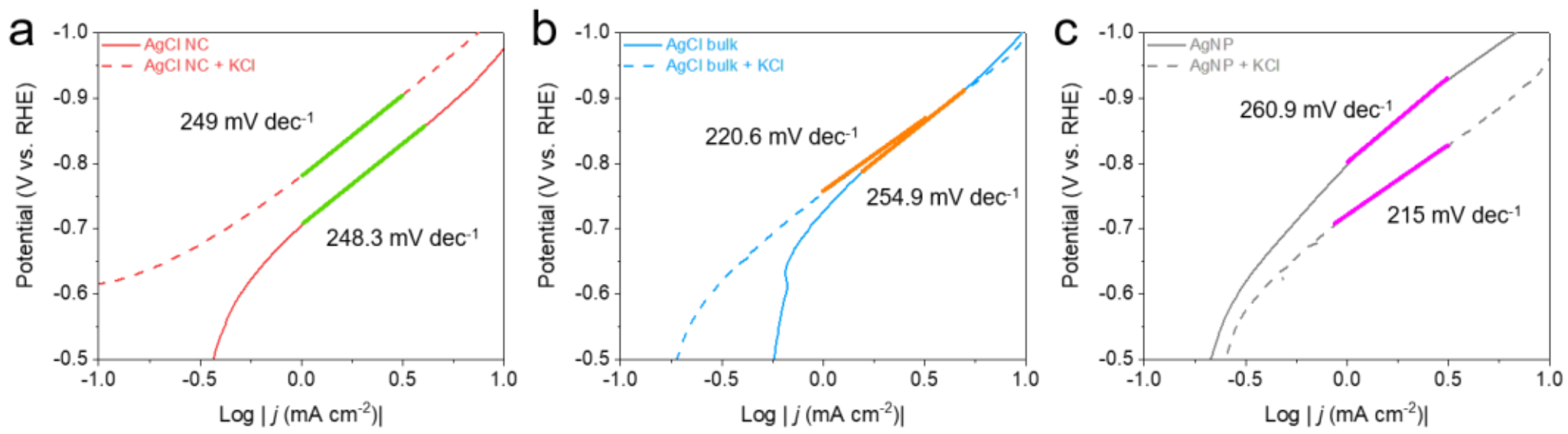

Figure S19. Tafel analysis of the catalysts. Differential Tafel slope plots derived from linear sweep voltammetry (LSV) measurements performed at a scan rate of 5 mV s^{-1} and converted to the reversible hydrogen electrode (RHE) scale. The plots are presented for the kinetically dominant region (log f ≈ 0–0.5) for (a) AgCl NC, (b) AgCl bulk, and (c) Ag NP in 0.1 M $KHCO_3$ and (0.09 M $KHCO_3$ + 0.01 M KCl) electrolytes.

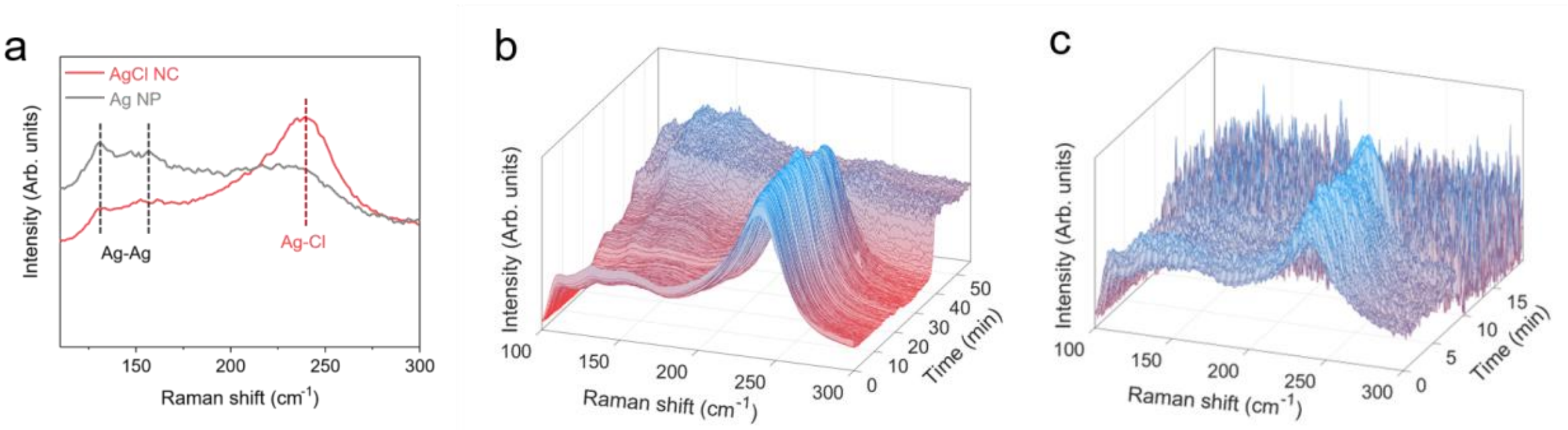

Figure S20. In-situ Raman analysis of AgCl NC and AgCl bulk during CO_2RR. (a) Reference spectra of Ag–Ag (135, 165 cm^{-1}) and Ag–Cl (245 cm^{-1}) bonds. Time-resolved 3D Raman spectra of (b) AgCl NC and (c) AgCl bulk during CO_2RR at reductive current density of 5 mA cm^{-2}.

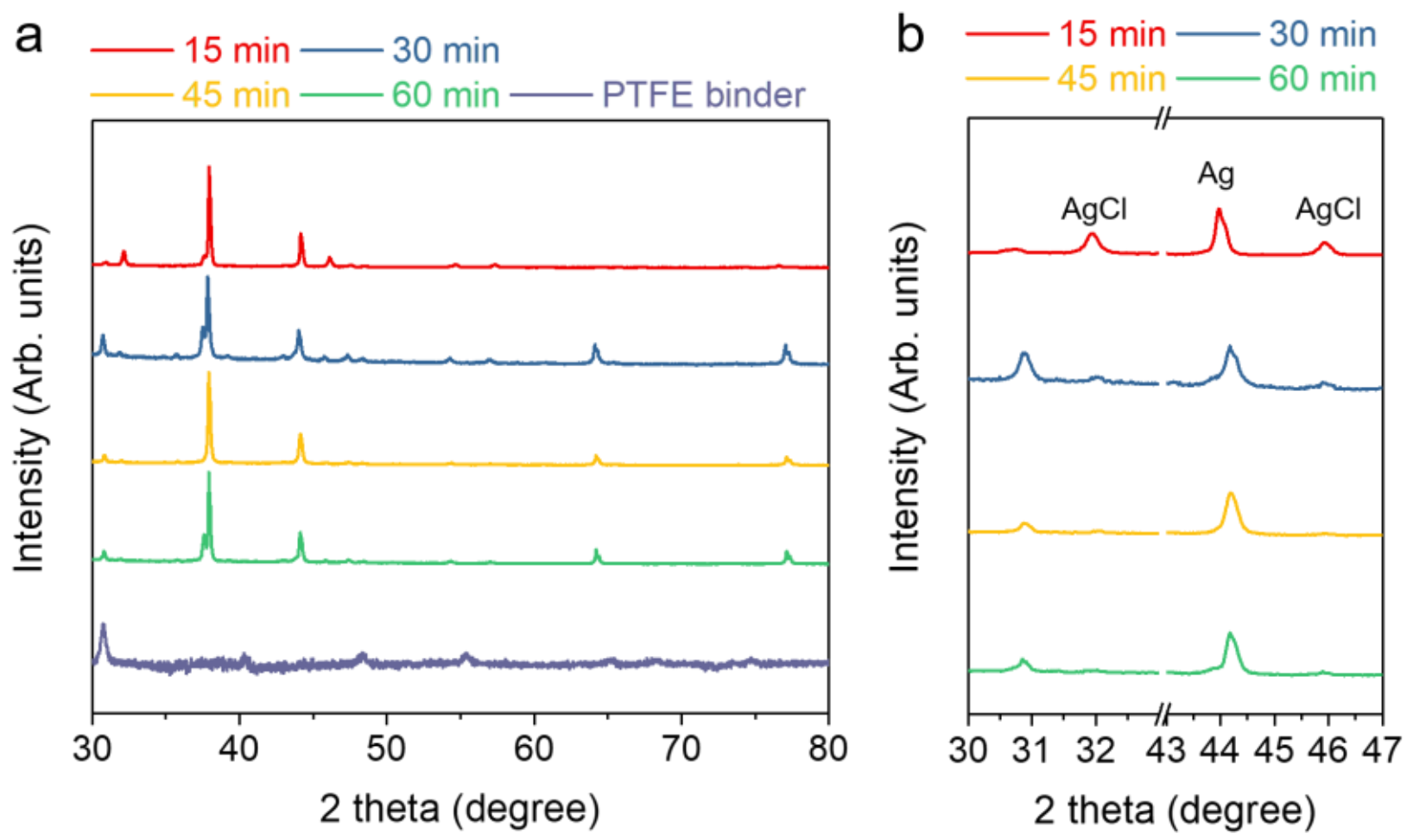

Figure S21. Time-dependent ex-situ XRD patterns of the activated electrodes. (a) Full-range XRD patterns (30°–80°) of the electrodes activated for 15, 30, 45, and 60 minutes, along with the pristine PTFE binder. (b) Magnified XRD patterns in the range of 30°-33° and 43°-47°, highlighting the gradual disappearance of the AgCl diffraction peaks at approximately 32° and 46° as the activation time increases.

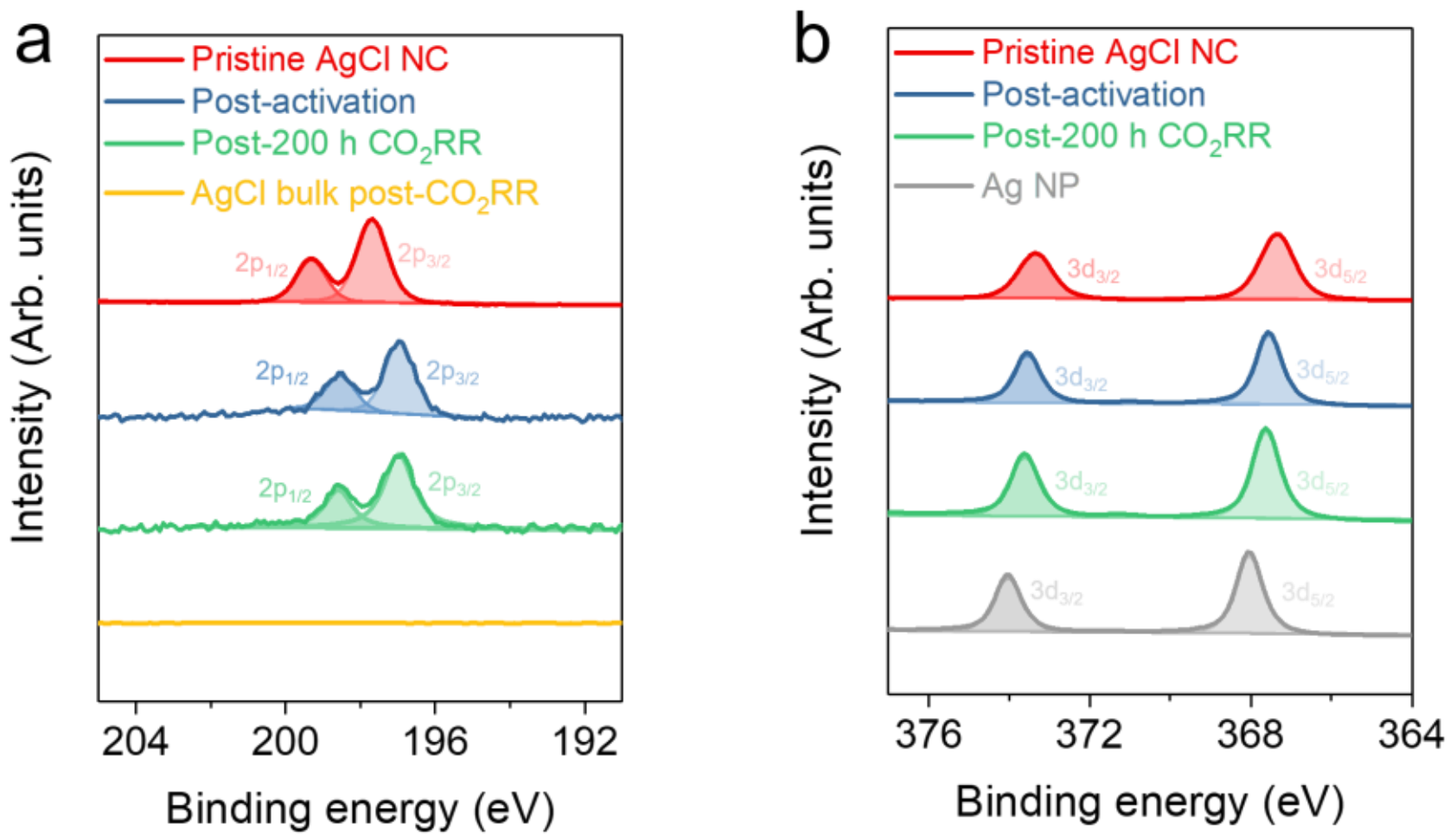

c

Sample	Pristine AgCl NC	Post-activation	Post-200 h CO_2RR
Cl/Ag ratio	0.95	0.06	0.04

Figure S22. XPS characterization of the AgCl NC and catalysts. (a) Cl 2p XPS spectra of pristine AgCl NC, AgCl NC post-activation, AgCl NC post-200 h CO_2RR, and AgCl bulk post-CO_2RR. AgCl bulk post-CO_2RR exhibited no detectable Cl 2p signal. The binding energies for Cl $2p_{3/2}$ and Cl $2p_{1/2}$ are 197.7 eV and 199.3 eV for pristine AgCl NC, 197.0 eV and 198.5 eV for post-activation, and 197.0 eV and 198.6 eV for post-200 h CO_2RR, respectively. (b) Ag 3d XPS spectra of pristine AgCl NC, AgCl NC post-activation, AgCl NC post-200 h CO_2RR, and Ag NP. The binding energies for Ag $3d_{5/2}$ and Ag $3d_{3/2}$ are 367.3 eV and 373.4 eV for pristine AgCl NC, 367.6 eV and 373.6 eV for post-activation, 367.6 eV and 373.6 eV for post-200 h CO_2RR, and 368.0 eV and 374.0 eV for Ag NP, respectively. (c) Atomic ratio of Cl/Ag for pristine AgCl NC, post-activation, and post-200 h CO_2RR samples calculated from the corresponding XPS spectra.

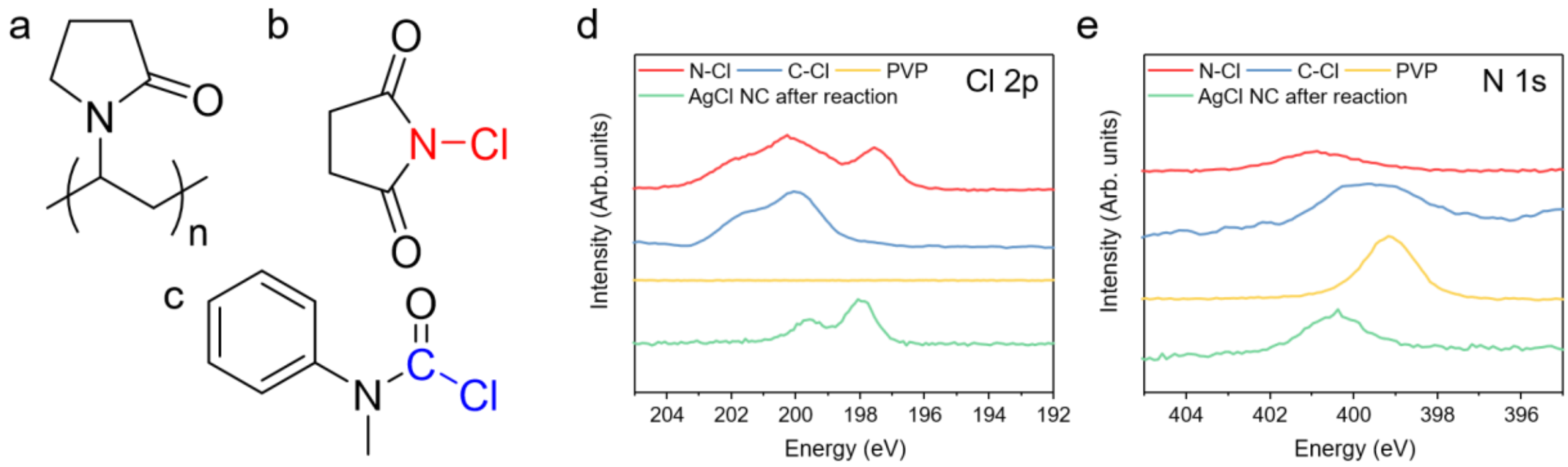

Figure S23. XPS analysis and molecular structures of Cl-containing compounds and AgCl NC. Molecular structures of (a) polyvinylpyrrolidone (PVP) (b) N-chlorosuccinimide, and (c) N-methyl-N-phenylcarbamoyl chloride. (d) Cl 2p and (e) N 1s XPS spectra of N-chlorosuccinimide (referred to as N-Cl in the legend), N-methyl-N-phenylcarbamoyl chloride (referred to as C-Cl), PVP, and AgCl NC after CO_2RR.

Note: To examine the binding of Cl species, XPS spectra were obtained for N-chlorosuccinimide, N-methyl-N-phenylcarbamoyl chloride, PVP, and AgCl NC electrode after CO_2RR. The compounds shown in (b) and (c) were selected as model molecules representing Cl bound to the nitrogen and carbon moieties of PVP, respectively. By comparing the Cl 2p and N 1s spectra, we suggest that the residual Cl species after CO_2RR are likely bound to PVP, indicating the formation of PVP–Cl interactions in AgCl NC.

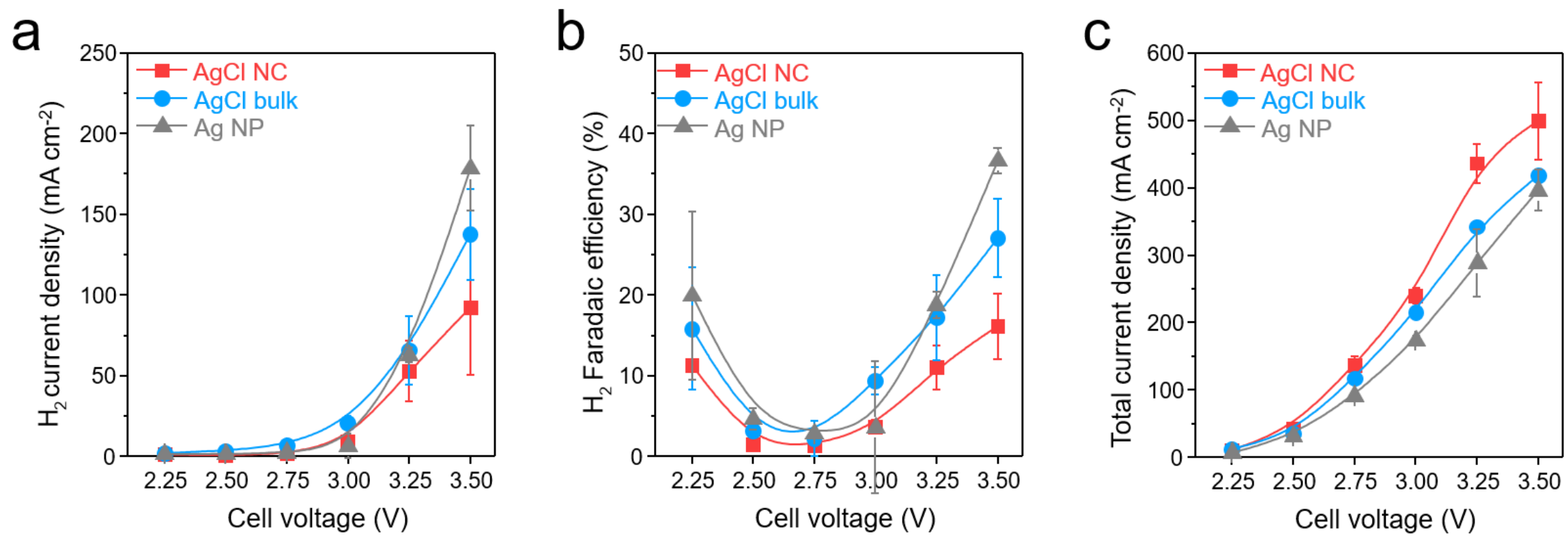

Figure S24. HER of AgCl-based catalysts and Ag NP in a MAE cell. (a) Partial current densities and (b) Faradaic efficiencies for H_2. All data were collected in CO_2-saturated 0.1 M $KHCO_3$ at voltages ranging from 2.25 to 3.5 V. Error bars represent the standard deviation calculated from at least 3 data points.

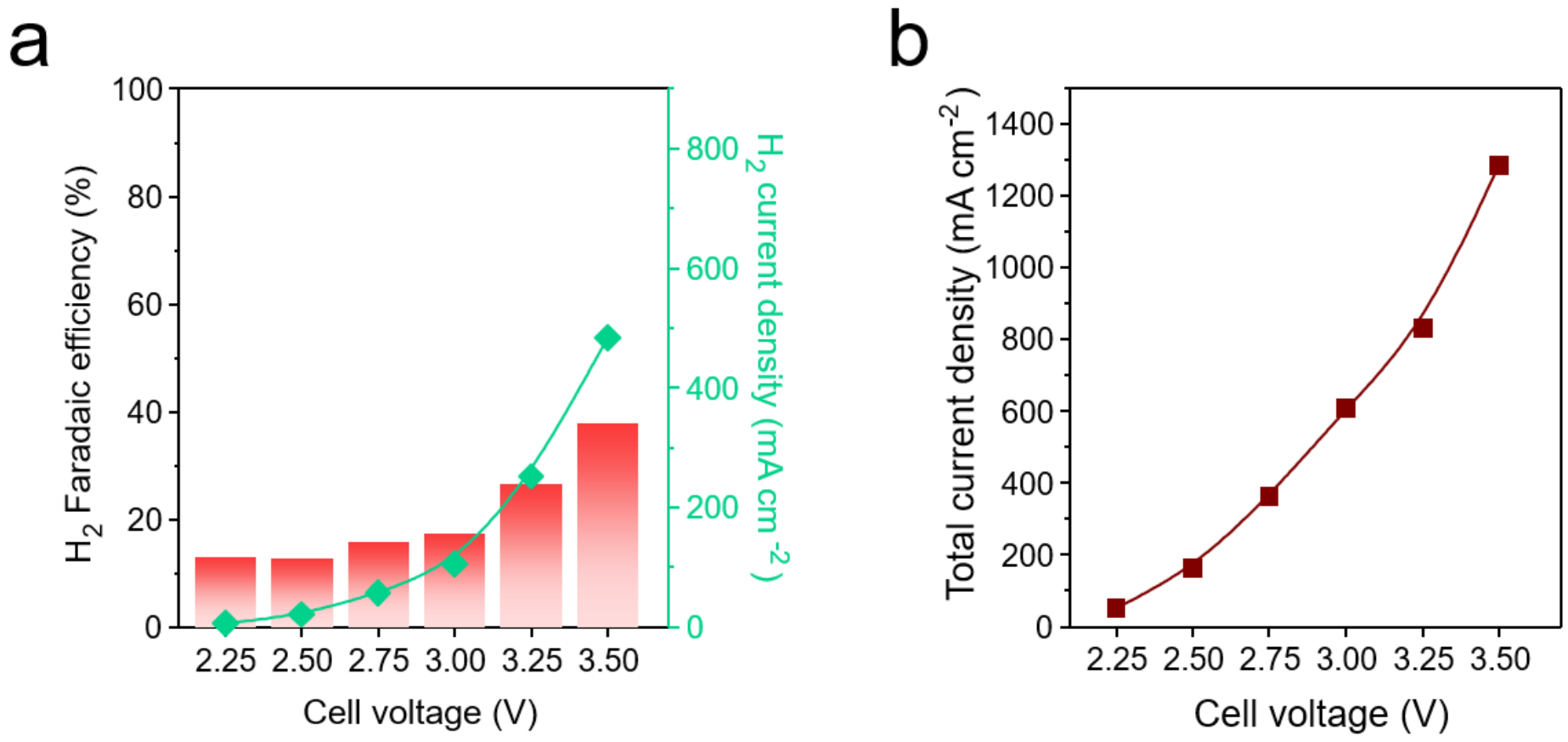

Figure S25. CO_2RR performance of AgCl NC under optimized conditions. (a) Faradaic efficiency and partial current density for H_2 and (b) total current density for overall reaction. All data were compared with those obtained in CO_2-saturated 0.5 M $KHCO_3$ at 60 °C in the MEA cell, at applied voltages ranging from 2.25 to 3.5 V.

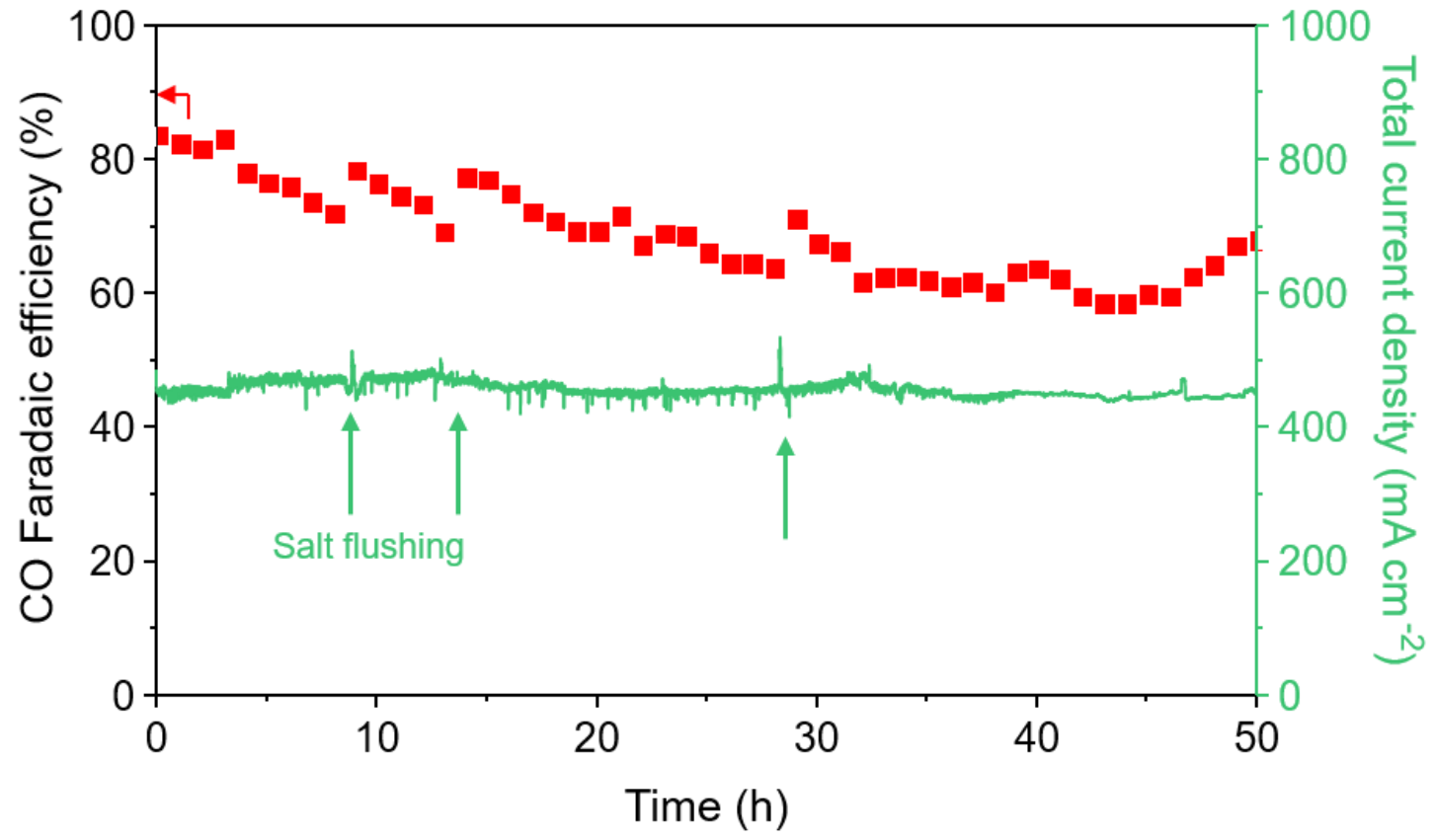

Figure S26. CO_2RR stability evaluation of the catalysts in a high-current-density MEA cell at 3.0 V and 60 °C in 0.5 M $KHCO_3$. Acid-humidification (0.2 M HCl, 100% RH at 60 °C) was employed to suppress salt precipitation, facilitating stable operation over 50 h. The sustained performance demonstrates that the catalytic activity is intrinsically stable, indicating that the initial decline in performance is not caused by catalyst degradation.

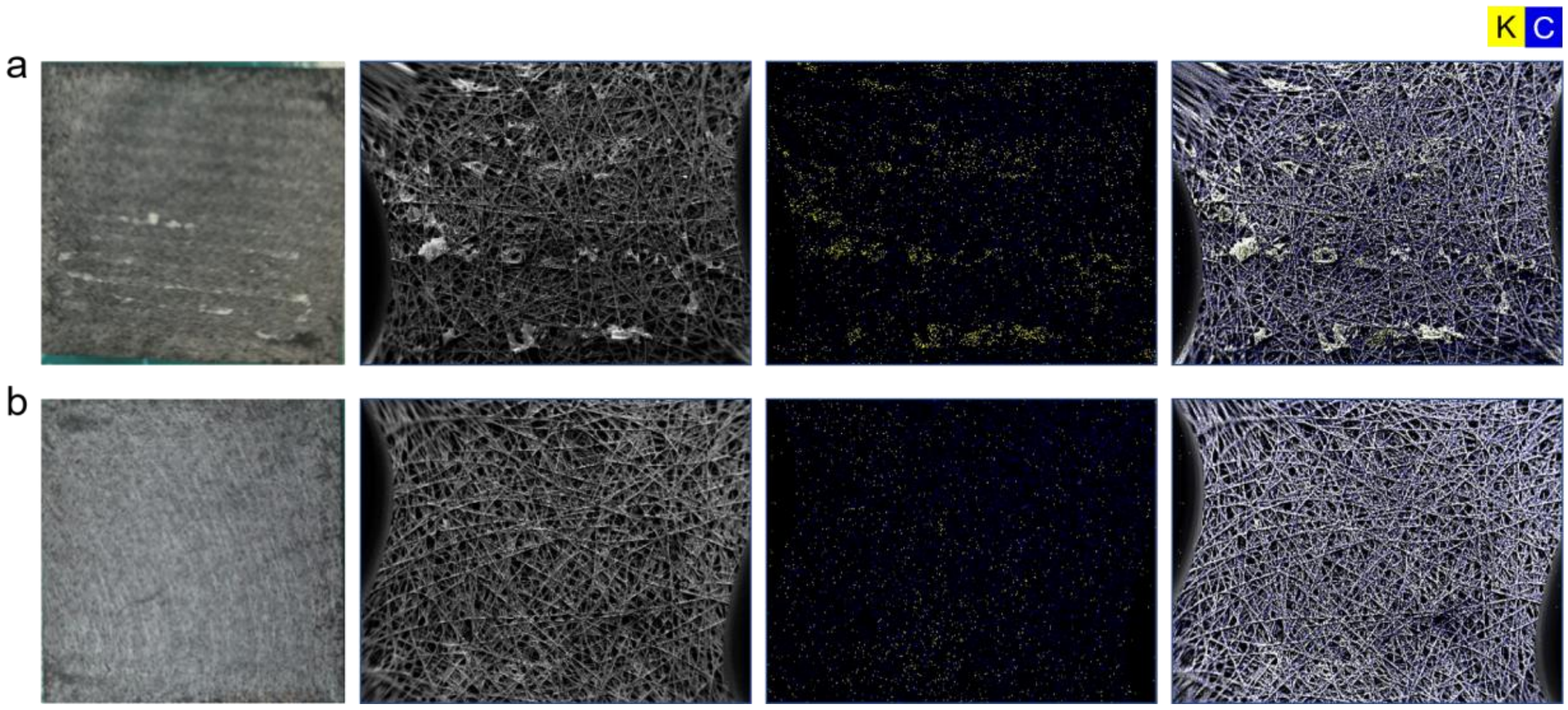

Figure S27. Characterization of the cathode gas diffusion layer (GDL) before and after DI water flushing during long-term CO_2 electrolysis. (a) Cathode GDL after 40 h operation without DI water flushing. (b) Cathode GDL after 40 h operation followed by DI water flushing. Each panel shows (from left to right) a photograph of the electrode backside, a top-view SEM image of the GDL, an EDS mapping image of potassium, and an overlaid SEM/EDS image showing the distributions of potassium and carbon.

Note S4. Catalyst production process simulations

Simulation models were developed in Aspen Plus V14 (Aspen Technology, Bedford, MA, USA) to analyze the production processes of two silver-based catalysts: Ag NP and AgCl NC. These models were designed to capture key process dynamics by integrating experimental synthesis methods, thermodynamic data, and operational parameters, enabling a systematic evaluation of their performance.

In modeling the synthesis and purification steps involved in AgCl NC and Ag NP production, it was essential to apply a thermodynamic model capable of accurately capturing the behavior of mixed electrolyte systems. The ELECNRTL (Electrolyte Non-Random Two-Liquid) model in Aspen Plus was selected due to its reliability in handling aqueous systems containing multiple dissociating salts, such as $AgNO_3$, KNO_3, $NaBH_4$, and H_3BO_3. Given the presence of strong ionic interactions and non-ideal mixing effects in the aqueous phase, particularly during washing and separation steps, the use of a model designed for electrolytic environments was critical.

The robustness of this thermodynamic framework is specifically demonstrated in Figure S28, where experimental binary data from NIST for the solvent systems (a) IPA-water and (b) ethanol-water are compared with model estimates. The T-xy diagrams show excellent agreement between the experimental VLE data and the ELECNRTL predictions across the entire composition range. This high degree of consistency, particularly near the azeotropic regions as shown in Figure S28, confirms that the model is highly accurate and suitable for simulating the complex separation and recovery processes. Consequently, this validated framework enabled more realistic predictions of salt solubility and phase equilibria, which were key to estimating water demand, separation efficiency, and waste stream composition.

The simulated process flows for each catalyst were constructed to reflect their experimentally verified synthesis routes, as shown in Figure 4. For the conventional Ag NP process, $AgNO_3$ is dissolved in a mixed ethanol-water solvent and reduced by $NaBH_4$ under continuous stirring. The reduction proceeds at 80 °C for 40 h in an agitated thermal reactor. Subsequently, hydrogen byproducts are vented, and the product slurry is subjected to centrifugation, washing, and drying to obtain Ag NP powder.

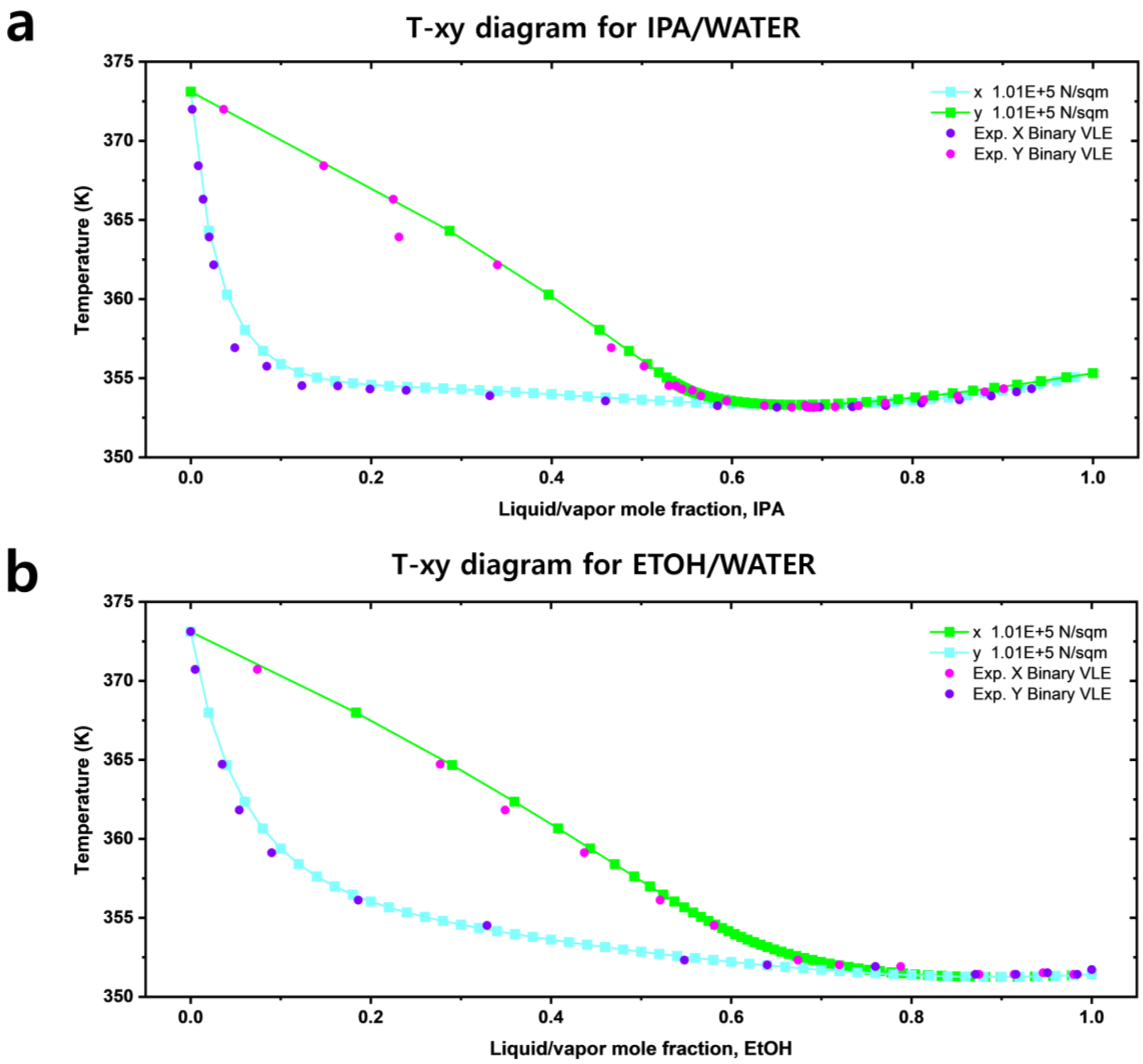

Figure S28. Binary experimental data of NIST and estimated results of ELECNRTL. (a) IPA-water,[1] (b) ethanol-water.[2] The vapor and liquid mole fractions are represented as x and y, respectively.

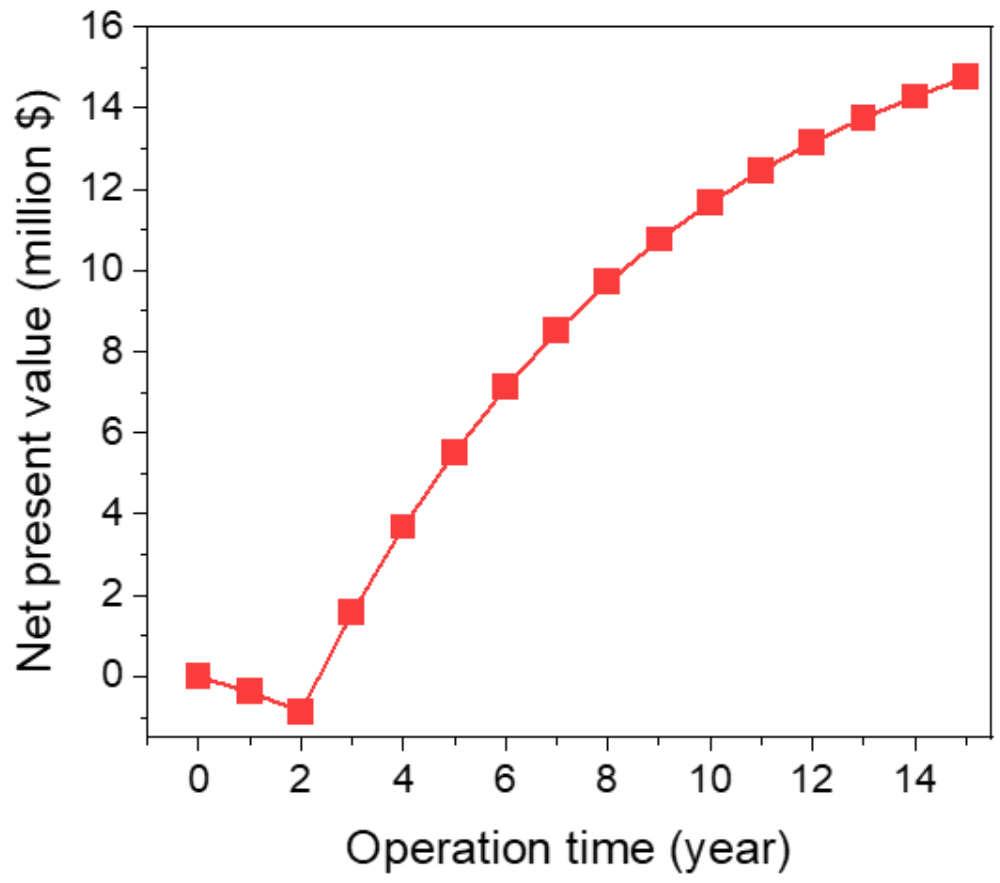

Figure S29. Net present value of AgCl NC production.

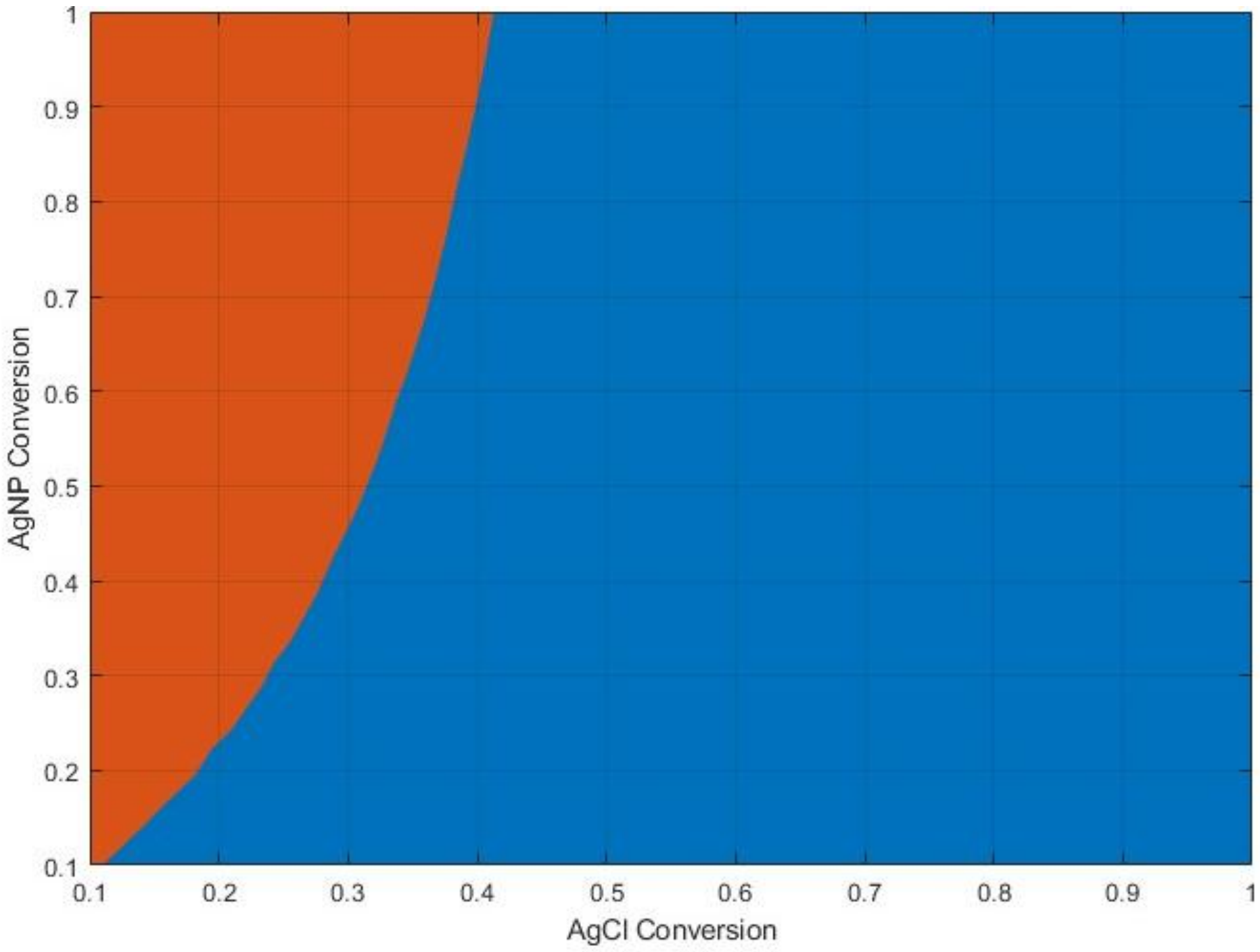

Figure S30. Catalyst cost superiority depending on conversion rates. The blue area indicates the conversion rates of when AgCl NC is more economically viable than Ag NP. The red area indicates the opposite case.

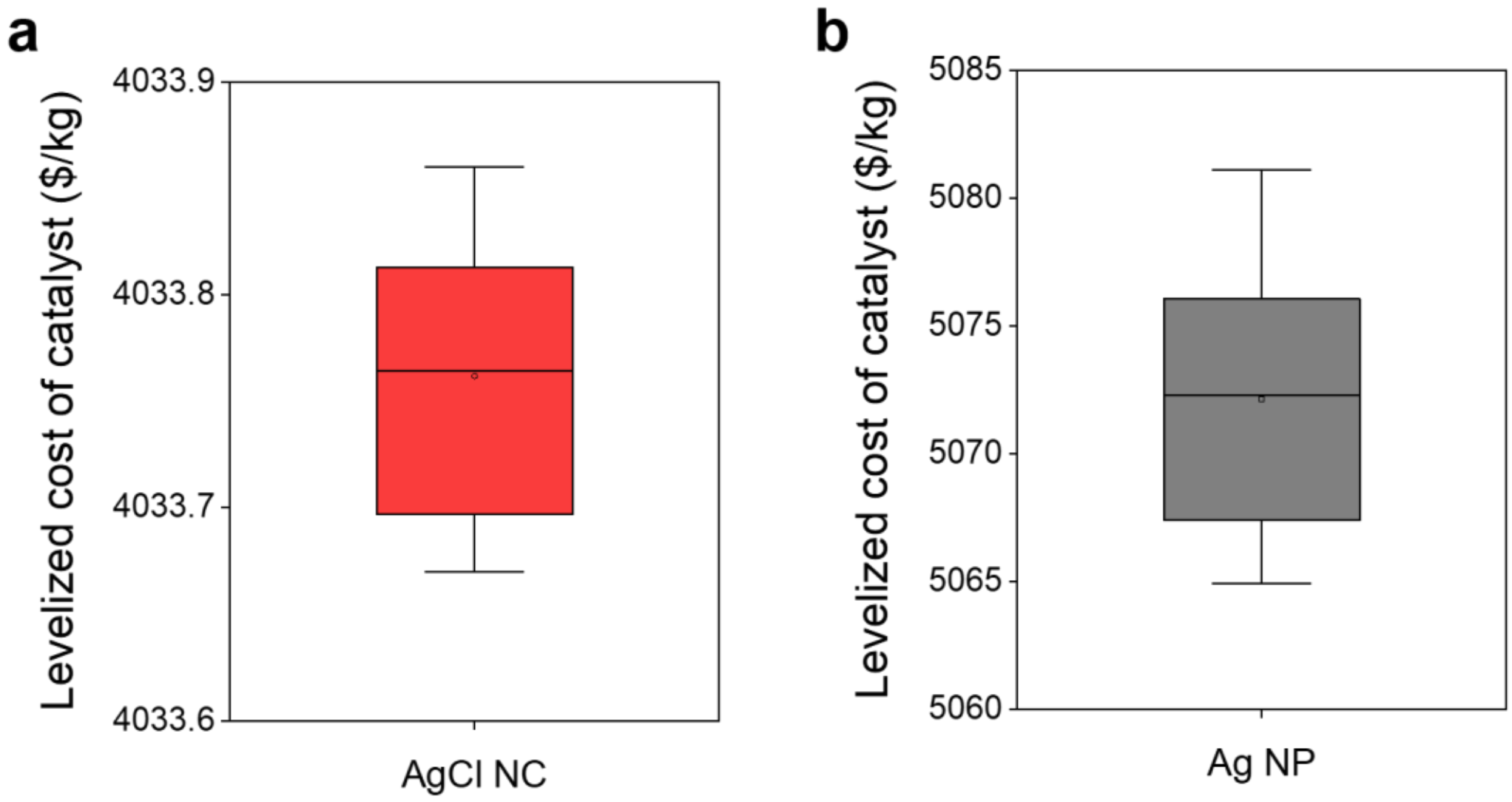

Figure S31. Effect of reactor retention time on levelized cost of catalysts. The levelized cost was recalculated for (a) AgCl NC and (b) Ag NP production processes with retention times randomly sampled between half and double their original values. The analysis confirms that retention time has minimal impact on the overall cost comparison between the two catalysts.

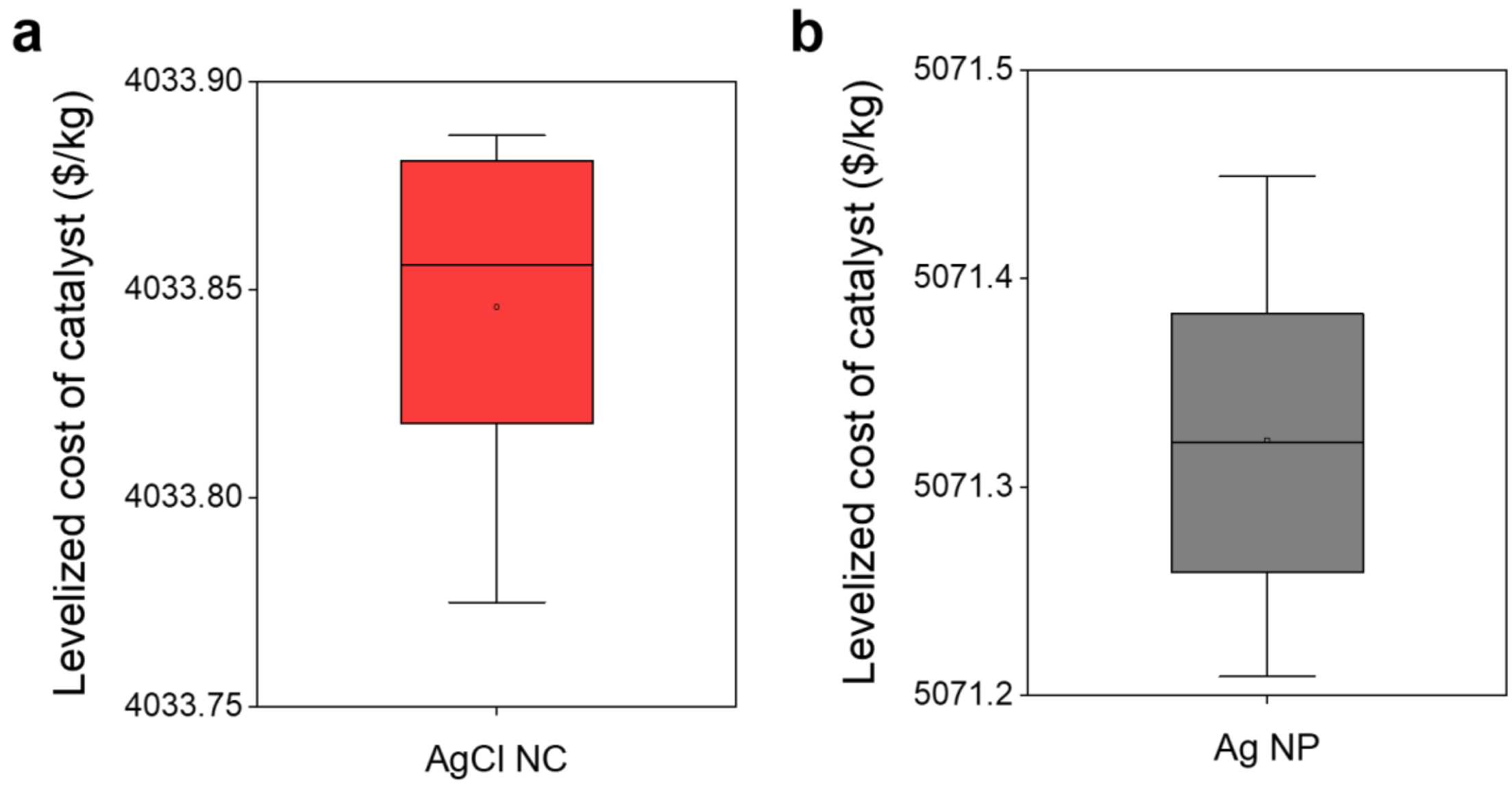

Figure S32. Effect of reaction temperature on levelized cost of catalysts. The levelized cost was recalculated for (a) AgCl NC and (b) Ag NP production processes with reaction temperatures randomly sampled between half and double their original values. The analysis confirms that temperature has minimal impact on the overall cost comparison between the two catalysts.

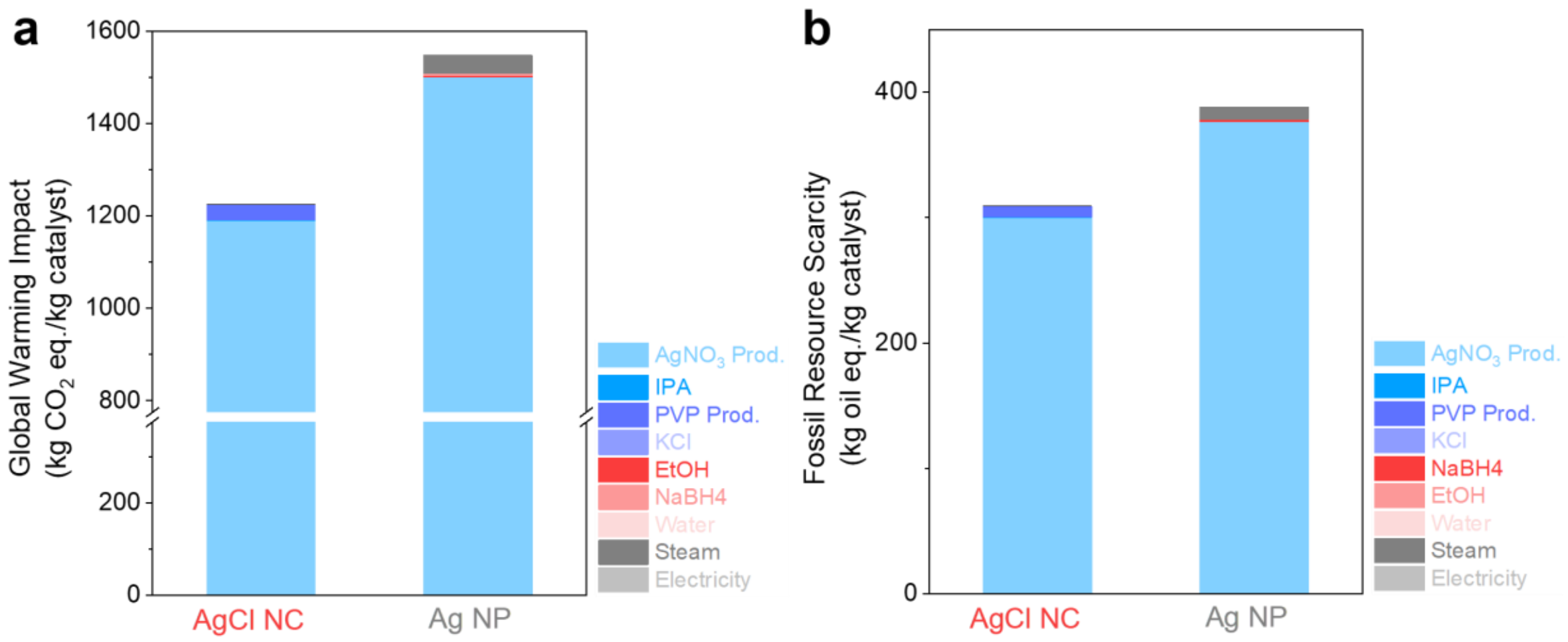

Figure S33. Life cycle assessment of the catalyst production processes. Life cycle assessments were evaluated regarding (a) global warming impact indices and (b) fossil resource scarcity. The life cycle inventories (LCIs) of all chemicals and utilities were obtained from the Ecoinvent V3.11 database, and environmental impact assessments were carried out using the ReCiPe 2016 v1.03, midpoint (H) method. The Ag NP process shows higher impacts than AgCl NC, mainly due to greater consumption of $AgNO_3$ during synthesis.

Note S5. Global sensitivity analysis.

A variance-based global sensitivity analysis (GSA) was conducted to quantify the influence of key techno-economic parameters on the levelized cost of carbon monoxide resulting from the CO_2 electroreduction process. First-order Sobol sensitivity indices were computed to determine the relative contribution of individual input parameters to the output variance, based on the formulation:

$$S_i = \frac{var[E(y|x_i)]}{var(y)}$$

where S_i represents the first-order Sobol index for the ith variable, $\mathrm{var(y)}$ denotes the total variance of the output, and $\mathrm{var[E(y|}x_i\mathrm{)]}$ quantifies the variance of the expected output when the variable or set of variables x_i is held constant.

In this work, GSA was used to evaluate the sensitivity of five primary cost-driving parameters for the catalyst production processes in Figure S34: $AgNO_3$ price, conversion rate, tax rate, interest rate, and post-treatment cost. This method was also used to evaluate the sensitivity of five parameters for CO production process as shown in Figure S35: Faradaic efficiency of CO, current density, cell voltage, electricity price, and maintenance period of the CO production process. These parameters span both electrochemical performance metrics and operational cost factors.

The application of this analysis enabled an efficient and robust assessment of parameter importance under uncertainty, with the first-order indices offering sufficient resolution to identify dominant cost contributors and guide system optimization.

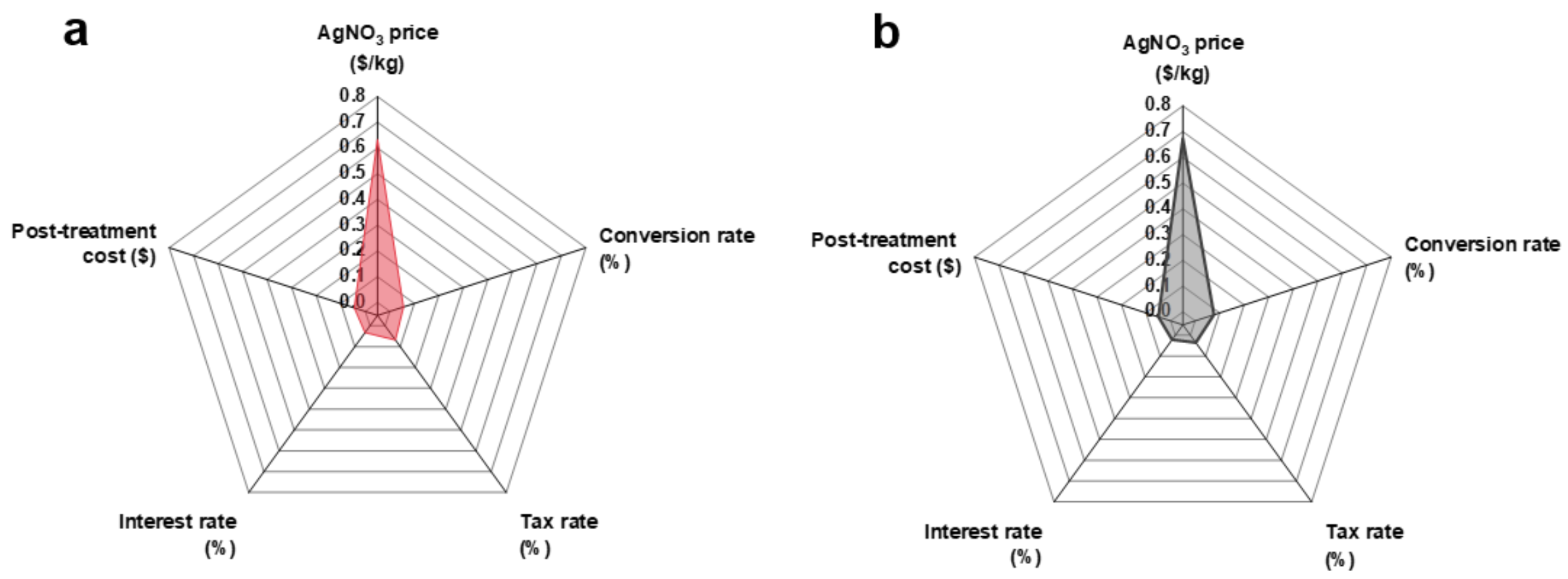

Figure S34. Global sensitivity analysis on catalysts. Global sensitivity analysis on (a) AgCl NC production cost and (b) Ag NP production cost. The sensitivity of five parameters: $AgNO_3$ price, conversion, tax rate, interest rate, and post-treatment cost, on the final catalyst production price were calculated via GSA. Due to the high material cost, the $AgNO_3$ price has the largest impact on the production costs of both catalysts.

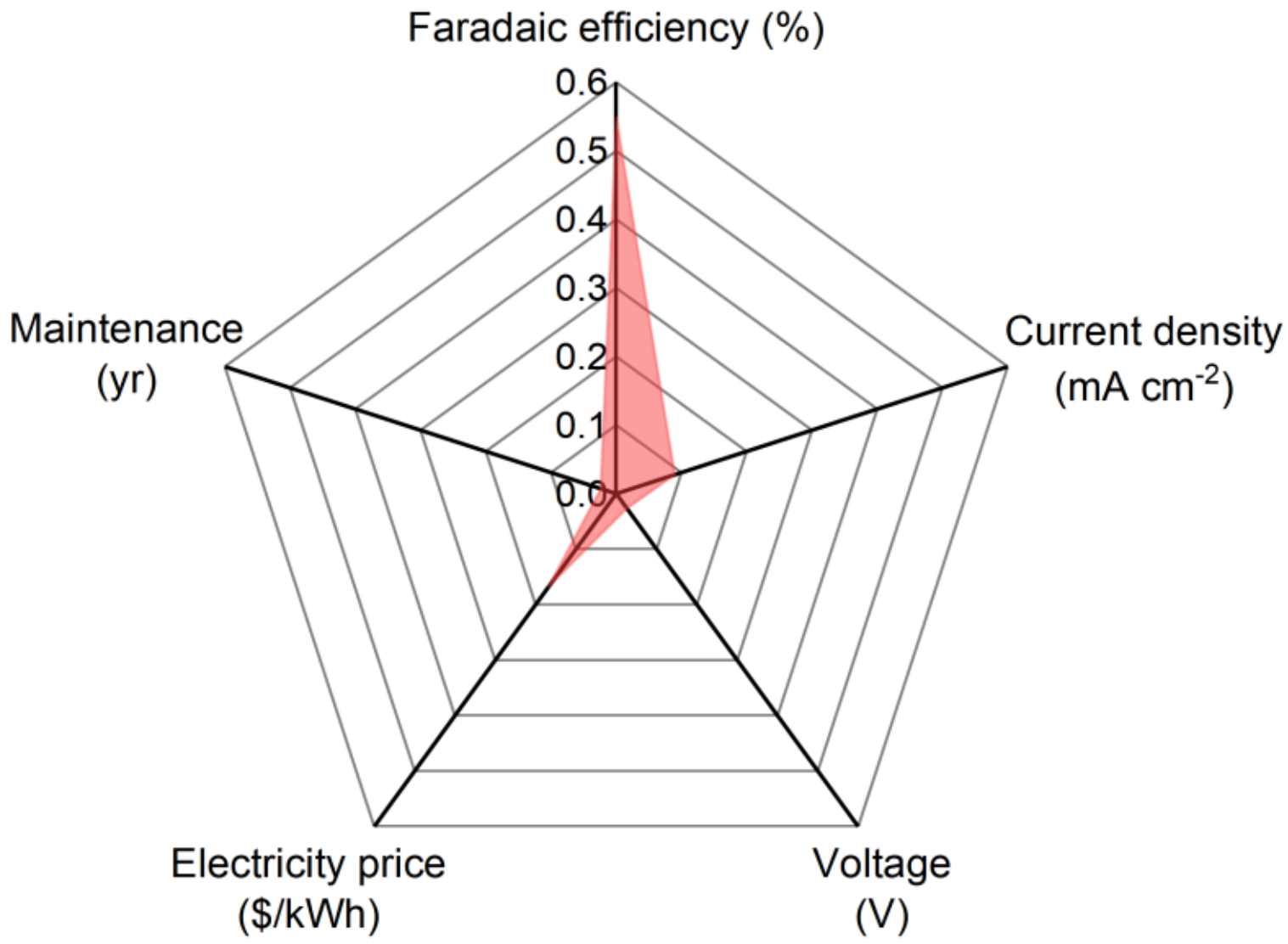

Figure S35. Global sensitivity analysis on CO production. Global sensitivity analyses of five key cost parameters: Faradaic efficiency, current density, voltage, electricity price, and maintenance period.

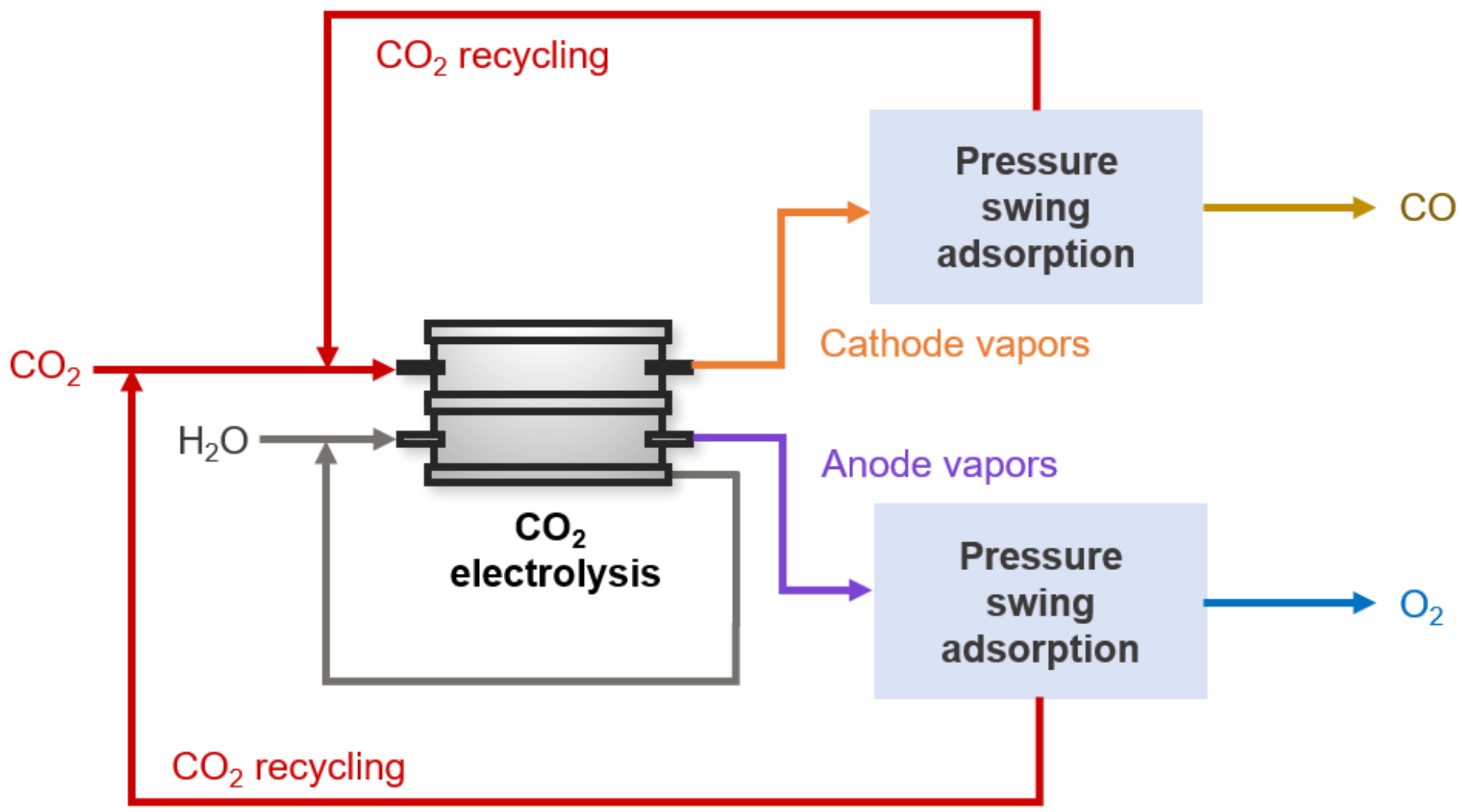

Figure S36. Block flow diagram of CO production process. Purchased CO_2 undergoes electrolysis to produce CO at the cathode. The vapor products from both cathode and anode are transferred to pressure swing adsorption processes, with unreacted CO_2 being recycled. The CO production process was assumed to produce 3.5 tons of CO per day. Further calculations were proceeded using the same method as Jiao et al. The CO_2RR performance data at 3.5 V from Figure 3a and b was used to calculate the overall costs.[3]

Note S6. Example calculation of electrolyzer cost.

The cost of the electrolyzer used in the CO production process TEA was calculated based on the reference costs shown in the work by Chae et al.[4] The total electrolyzer reference cost was 7,200 USD per m^2, with 43.85, 28.66, 15.09, 12.26, 0.14% accounting for the bipolar membrane (BPM), IrO_x catalyst, Ti support, Ag catalyst, and frame, respectively. The membrane cost was replaced with that of anion exchange membrane (AEM),[5] and the Ag catalyst cost with the levelized costs calculated from the catalyst production process TEA. The cost breakdown of the electrolyzer is shown in Figure S37.

1. IrO_x catalyst

$$7200\ \frac{USD}{m^2} \times 0.2866 = 2063.52\frac{USD}{m^2}$$

2. Ti support

$$7200\ \frac{USD}{m^2} \times 0.1509 = 1086.48\frac{USD}{m^2}$$

3. Frame

$$7200\ \frac{USD}{m^2} \times 0.0014 = 10.08\frac{USD}{m^2}$$

4. Ag catalyst

$$0.009\frac{kg}{m^2} \times 4188\frac{USD}{kg} = 37.69\frac{USD}{m^2}$$

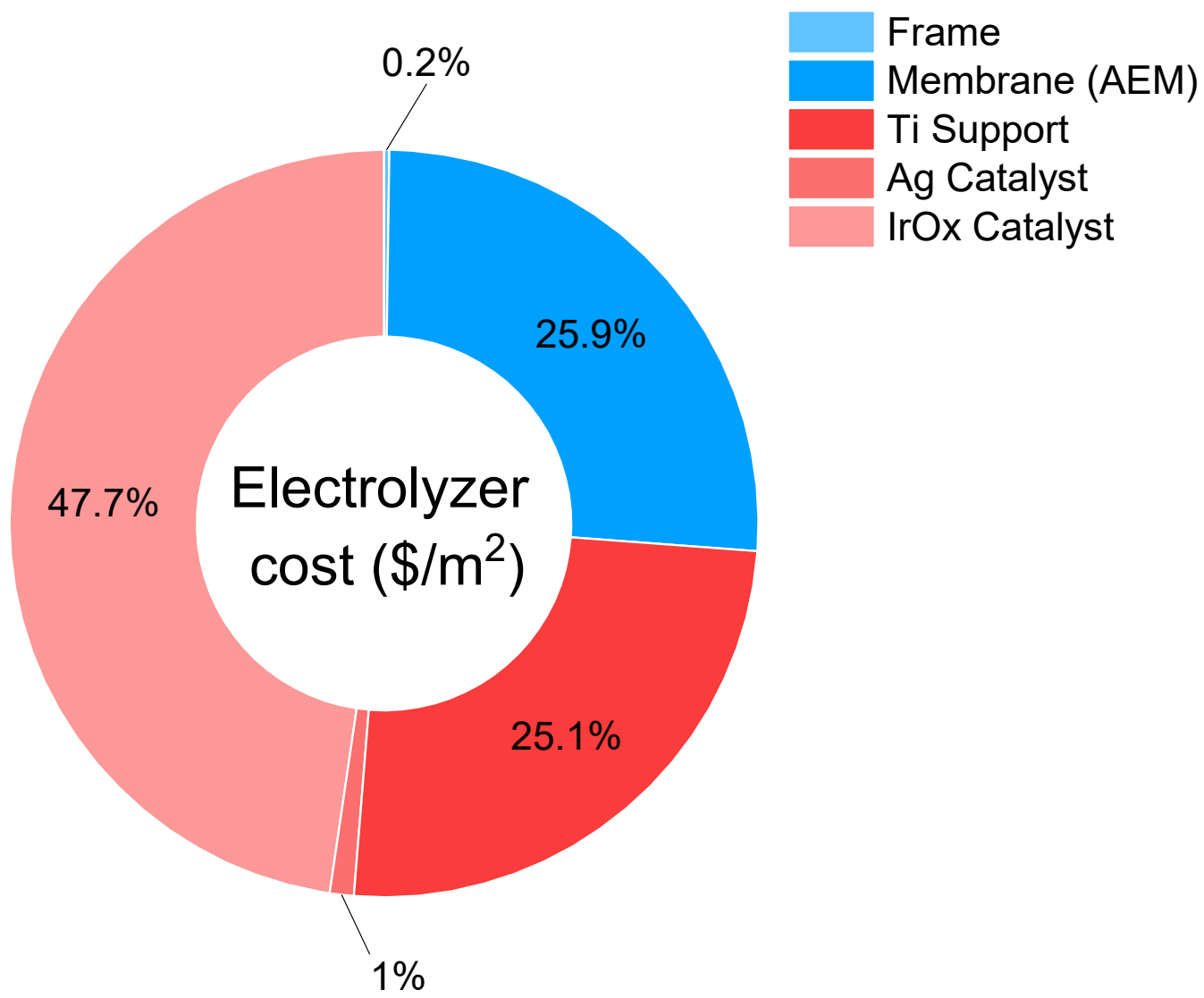

Figure S37. Electrolyzer cost breakdown. Detailed calculations are provided in Note S6.

Supporting Tables

Table S1. Literature survey of Ag-based nanoparticle synthesis methods.

No.	Catalyst	Synthesis method	Reaction time (hr)	Batch throughput (mg)	Ref.
1	AgCl NC	Synthesized by sonochemical method using $AgNO_3$ and KCl with PVP as a capping agent	0.083	2720	This work
2	Ag-TEL	Synthesized by in situ electrodeposition on HNO_3-treated carbon felt in $AgNO_3$/$KHCO_3$ solution under CO_2 bubbling, followed by l-cysteine modification	13	100	[6]
3	Ag/C	Synthesized by drop-casting silver acetate with carbon and Nafion onto carbon paper, followed by annealing at 250 °C for 2 h	4	16.9	[7]
4	Ag dendrite	Synthesized by electrodeposition of Ag in ethylenediamine electrolyte under N_2	1	3.19	[8]
5	Ag-NOLI	Synthesized by thermal decomposition of silver acetate with tetradecylphosphonic acid in trioctylamine at 130 °C under N_2	2	53.9	[9]
6	e-Ag coral	Synthesized by e-beam evaporation of Ag, followed by 12 h electrochemical oxidation in Ar-saturated KCl and 30 min reduction in CO_2-saturated $KHCO_3$	12.5	5	[10]
7	Ag@PPy	Synthesized by citrate-assisted reduction of $AgNO_3$ with $NaBH_4$ under stirring and sonication in pyrrole/$K_2S_2O_8$ solution	4	40	[11]
8	TMAH-treated Ag-NP	Synthesized by thermal decomposition of $AgNO_3$ in oleylamine at 160 °C, followed by purification, 2 h tetramethylammonium hydroxide pentahydrate (TMAH) sonication, and dispersion in hexane/isopropanol	4	215.7	[12]
9	Ag NP	Synthesized using citrate and PVP, H_2O_2, and $NaBH_4$, then stirred, filtered, and stored at 4 °C in the dark	3	0.510	[13]
10	Ag seed NP	Synthesized using cetyltrimethylammonium bromide (CTAB) and $NaBH_4$, with 1 hr dark aging	1	8.50	[14]
11	23 nm Ag-NP seeds	Synthesized using sodium citrate, $AgNO_3$, NaCl, and ascorbic acid, with boiling water injection and 1 hr heating	1	1.59	[15]
12	40-300 nm Ag NP	Synthesized via seed-mediated growth using silver–ammonia complex and ascorbic acid, with size controlled by seed amount	2	1.91	[16]
13	Ag NP	Synthesized by thermal decomposition of silver trifluoroacetate in isoamyl ether with oleic acid	2	25.8	[17]
14	Ag NP	Synthesized using $NaBH_4$ and trisodium citrate, with stepwise heating to 60 °C and 90 °C, and pH adjustment to 10.5 during reaction	1	2.16	[18]
15	Ag NC	Synthesized in ethylene glycol using PVP and HCl, with controlled injection of $AgNO_3$ and PVP solutions at 140 °C, followed by 20 hr aging	29	30.4	[19]
16	Ag NP	synthesized via solvothermal reduction of $AgNO_3$ with $NaBH_4$ under reflux at 80 °C for 40 h	40	1062	[20]

Table S2. Literature survey of CO_2RR performance using Ag-based catalysts in zero-gap configurations.

No.	Catalyst	Cell configuration	Electrode area (cm^2)	j_{CO} ($mA\ cm^{-2}$)	Voltage (V)	FE_{CO} (%)	Stability (h)	Electrolyte	Ref.
1	AgCl NC	MEA cell	5	761	3.5	60		0.5 M $KHCO_3$	This work
				210	3.0	95	200	0.1 M $KHCO_3$	
2	D-Ag/C+P-Id	MEA cell	1	1080	3.89	90		0.2 M CsOH	[21]
				375	2.89	94	20		
3	Ag NP	MEA cell	8	1004	3.4	91		0.1 M CsOH	[22]
				450	3.2	90	150	0.01 M CsOH	
4	Ag-SiO_2	MEA cell	10	806	4.0	80		0.1 M $CsHCO_3$	[23]
				600	3.75	85	20		
5	Nano Ag mesh	MEA cell	4	633	7.2	90.4		0.7 M $KHCO_3$	[24]
				180	3.6	90	350		
6	Ag crystal–triazole	MEA cell	4	547	4.0	96.1		0.1 M $KHCO_3$	[25]
				282	3.5	94	70		
7	Ag@PiperION	MEA cell	4	540	5.2	90		0.1 M Cs_2CO_3	[26]
				225	3.2	75	220		

8	Ag NP	MEA cell	5	440	4.0	90		0.1 M $KHCO_3$	[27]
				125	3.6	76	12		
9	porous Ag	MEA cell	1	368	3.6	74		0.1 M $KHCO_3$	[28]
				233	3.5	70	25		
10	e-coral Ag	MEA cell	10	312	3.5	90		1 M KOH	[10]
				70	2.75	70	30		
11	TMAH-AgNP	MEA cell	10	298	3.4	92		0.1 M $KHCO_3$	[12]
				230	3.0	92	100		
12	Ag dendrite	MEA cell	5	105	3.0	94		0.1 M $KHCO_3$	[8]
				80	3.0	80	100		
13	Ag-TEL	FTDF cell	1	3370	4.5	92		0.5 M $KHCO_3$	[6]
				1612	3.5	92	210		

*FTDT (Flow-through-induced dynamic triple-phase boundary)

* j_{CO}, voltage, and FE_{CO} values are reported for both the maximum j_{CO} condition and the separate stability test condition.

Table S3. CAPEX calculations of the catalyst production processes.

a) Capital cost calculation of the catalyst production processes.[29]

Cost factor		AgCl NC Cost ($MM)	Ag NP Cost ($MM)
Installed equipment (EQP)			
Agitator		0.0011	0.0011
Sonicator		0.017	-
Heater		0.0062	0.0060
Reactor		0.0016	0.23
Flash		0.00	0.0021
Centrifuge		0.23	0.23
Washer		0.11	0.11
Dryer		0.24	0.24
Computers and software		0.02	0.020
TOTAL BARE-MODULE INVESTMENT (TBM)		**0.69**	**0.84**
Cost of site preparation	10% × C_{TBM}	0.069	0.084
DIRECT PERMANENT INVESTMENT (DPI)		**0.76**	**0.93**
Cost of contingencies and contractor's fee	15% × C_{DPI}	0.11	0.14
TOTAL DEPRECIABLE CAPITAL (TDC)		**0.88**	**1.07**
Cost of land	2% × C_{TDC}	0.018	0.021
Cost of royalties	2% × C_{TDC}	0.018	0.021
Cost of plant startup	2% × C_{TDC}	0.018	0.021
TOTAL PERMANENT INVESTMENT (TPI)		**0.93**	**1.1**
Working capital	20% × C_{TPI}	0.19	0.23
TOTAL CAPITAL INVESTMENT (TCI)		**1.12**	**1.36**

b) Specifications for sonicator cost estimation.[30]

	Units	Value
Sonicator probe	$/probe	1250
Probe capacity	L/probe	20

Table S4. OPEX calculations of the catalyst production processes.

a) Raw material cost specifications for AgCl NC production process.[31-34]

**Material**	**Molar Mass**	**Density**	**Volumetric Flow Rate**	**Mass Flow Rate**	**Bulk Cost**	**Raw Materials Cost**
	g/mol	g/mL	L/hr	kg/hr	\$/kg (L)	\$MM/yr
$AgNO_3$	169.9	-	-	1.3	2255.2	24.5
KCl	74.6	-	-	0.6	0.35	0.002
IPA	60.1	0.785	0.36	0.28	1.2	0.48
PVP	111.1	-	-	8.3	4.5	0.16
Water	18.0	0.997	2.4	2.4	0.2	0.07
					Total	25.2

b) Raw material cost specifications for Ag NP production process.[34-36]

**Material**	**Molar Mass**	**Density**	**Volumetric Flow Rate**	**Mass Flow Rate**	**Bulk Cost**	**Raw Materials Cost**
	g/mol	g/mL	L/hr	kg/hr	\$/kg (L)	\$MM/yr
$AgNO_3$	169.9	-	-	1.7	2255.2	30.6
$NaBH_4$	37.8	-	-	0.4	160.1	0.5
EtOH	46.1	0.789	4.9	3.9	5.9	1.6
Water	18.0	0.997	7.3	7.3	0.2	0.01
					Total	32.7

c) Solubility data of byproducts in catalyst production processes.

**Species**	**Solubility (g/100 mL) at room temp.**
KNO_3	38
H_3BO_3	4.77
$NaNO_3$	91.2

d) Input parameters for OPEX calculations.[29]

Parameter	Units	Value
Electricity	$/kWh	0.10
Steam (50 psig)	$/J	3.1×10^{-9}
Cooling water	$/kg	0.02
Number of operators		10
Plant life	yr	15
Construction period	yr	2
Depreciation		MACRS-7yr
Interest rate	%	15
Tax rate	%	38.9

e) Operating costs of the catalyst production processes.[29]

Cost factor		AgCl NC Cost ($MM/yr)	Ag NP Cost ($MM/yr)
Utilities			
Material		25.2	32.7
Electricity		0.02	0.0014
Steam		0.01	0.0070
Cooling water (CW)		0.00	0.00
Total utilities		**25.2**	**32.7**
Operations (O)			
Direct wages and benefits (DW&B)	$35 per operator-hr	2.8	2.8
Direct salaries and benefits	15% × $C_{DW\&B}$	0.42	0.42
Operating supplies and services	6% × $C_{DW\&B}$	0.025	0.025
Technical assistance	$60,000/(operator/shift)-yr	0.20	0.20
Control laboratory	$65,000/(operator/shift)-yr	0.22	0.22
Total labor-related operations		**3.7**	**3.7**
Maintenance (M)			
Solid-fluid handling process	4.5% × C_{TDC}	0.039	0.048
Salaries and benefits	25% × $C_{MW\&B}$	0.0099	0.012
Materials and services	10% × $C_{MW\&B}$	0.039	0.048

Maintenance overhead	5% × $C_{MW\&B}$	0.0020	0.0024
Total maintenance		**0.091**	**0.11**
Total of M&O-SW&B		3.27	3.28
General plant overhead	7.1% × M&O-SW&B	0.23	0.23
Mechanical department services	2.4% × M&O-SW&B	0.078	0.079
Employee relations department	5.9% × M&O-SW&B	0.19	0.19
Business services	7.4% × M&O-SW&B	0.24	0.24
Total operating overhead		**0.74**	**0.75**
Property taxes and insurance	2% × C_{TDC}	**0.018**	**0.021**
Depreciation (D)			
Direct plant	8% × C_{TDC}	0.070	0.085
Total depreciation		**0.088**	**0.11**
COST OF MANUFACTURE (COM)		**29.8**	**37.4**
General expenses			
Transfer expense	3% of sales	1.6	2.4
Direct research	4.8% of sales	2.0	2.9
Allocated research	0.5% of sales	0.20	0.30
Administrative expense	2% of sales	0.82	1.19
Management incentive compensation	1.25% of sales	0.51	0.74
TOTAL GENERAL EXPENSES (GE)		**5.1**	**7.5**
TOTAL PRODUCT COST (C)		**35.0**	**44.8**
TOTAL SALES		**40.9**	**59.6**

Table S5. TEA of CO Production Process.

a) TEA baseline parameter for AgCl NC case.

Parameter	Units	Value
Product		Carbon monoxide
Membrane		AEM
Cell voltage	V	3.5
Current density	A cm^{-2}	0.407
Single-pass conversion*	%	30
Faradaic efficiency	%	72.6

* Assumption from Jiao et al.[3]

b) TEA baseline parameter for Ag NP case.

Parameter	Units	Value
Product		Carbon monoxide
Membrane		AEM
Cell voltage	V	3.5
Current density	A cm^{-2}	0.218
Single-pass conversion*	%	30
Faradaic efficiency	%	44.9

* Assumption from Jiao et al.[3]

References

1 A. S. Brunjes and M. J. P. Bogart. Vapor-Liquid Equilibrium Data for Commercially Important Systems of Organic Solvents Binary Systems n-Butanol–n-Butyl Acetate and n-Butanol–Acetone. *Ind. Eng. Chem.* **1935**, 27 (4), 396–400.

2 A. I. Johnson and W. F. Furter. Salt effect in vapor-liquid equilibrium, part II. *Can. J. Chem. Eng.* **2011**, 38 (3), 78-87.

3 H. Shin, K. U. Hansen and F. Jiao. Techno-economic assessment of low-temperature carbon dioxide electrolysis. *Nat. Sustain.* **2021**, 4 (10), 911-919.

4 S. Y. Chae, S. Y. Lee, S. G. Han, H. Kim, J. Ko, S. Park, O.-S. Joo, D. Kim, Y. Kang, U. Lee, Y. J. Hwang and B. K. Min. A perspective on practical solar to carbon monoxide production devices with economic evaluation. *Sustain. Energy Fuels* **2020**, 4 (1), 199-212.

5 B. Yang, R. Zhang, Z. Shao and C. Zhang. The economic analysis for hydrogen production cost towards electrolyzer technologies: Current and future competitiveness. *Int. J. Hydrog. Energy* **2023**, 48 (37), 13767-13779.

6 G. Wen, B. Ren, X. Wang, D. Luo, H. Dou, Y. Zheng, R. Gao, J. Gostick, A. Yu and Z. Chen. Continuous CO_2 electrolysis using a CO_2 exsolution-induced flow cell. *Nat. Energy* **2022**, 7 (10), 978-988.

7 Y. Ma, W. Niu, W. Shi, X. Huang, Y. Liu, J. Chen, L. Xue and B. Zhang. Amorphous carbon coating enhances activity of high rate CO_2 electroreduction to CO. *J. Mater. Chem. A* **2023**, 11 (23), 12114-12120.

8 A. Alihosseinzadeh, A. Unnikrishnan, K. Karan and S. Ponnurangam. Dendritic Ag Electrocatalyst with High Mass-Specific Activity for Zero-Gap Gas-Fed CO_2 Electroreduction. *ACS Appl. Energy Mater.* **2023**, 6 (3), 1533-1543.

9 D. Kim, S. Yu, F. Zheng, I. Roh, Y. Li, S. Louisia, Z. Qi, G. A. Somorjai, H. Frei, L.-W. Wang and P. Yang. Selective CO_2 electrocatalysis at the pseudocapacitive nanoparticle/ordered-ligand interlayer. *Nat. Energy* **2020**, 5 (12), 1032-1042.

10 W. H. Lee, Y.-J. Ko, Y. Choi, S. Y. Lee, C. H. Choi, Y. J. Hwang, B. K. Min, P. Strasser and H.-S. Oh. Highly selective and scalable CO_2 to CO - Electrolysis using coral-nanostructured Ag catalysts in zero-gap configuration. *Nano Energy* **2020**, 76, 105030.

11 Z. Wang, W. Wang, Q. Yang, Y. Geng, Y. Du, K. Liu, Z. Wu, J. Lai, B. Li, H. Li, G. Zheng and L. Wang. Highly Selective Acidic CO_2 Electroreduction with Large Current on Polypyrrole-Modified Ag Catalyst by Local Microenvironment Modulation. *Adv. Energy Mater.* **2025**, 15 (22), 2405419.

12 Y. J. Ko, C. Lim, J. Jin, M. G. Kim, J. Y. Lee, T. Y. Seong, K. Y. Lee, B. K. Min, J. Y. Choi, T. Noh, G. W. Hwang, W. H. Lee and H. S. Oh. Extrinsic hydrophobicity-controlled silver nanoparticles as efficient and stable catalysts for CO_2 electrolysis. *Nat. Commun.* **2024**, 15 (1), 3356.

13 T. Huang and X. H. Nancy Xu. Synthesis and Characterization of Tunable Rainbow Colored Colloidal Silver Nanoparticles Using Single-Nanoparticle Plasmonic Microscopy and Spectroscopy. *J. Mater. Chem.* **2010**, 20 (44), 9867-9876.

14 H. Hegde, C. Santhosh and R. K. Sinha. Seed mediated synthesis of highly stable CTAB capped triangular silver nanoplates for LSPR sensing. *Mater. Res. Express* **2019**, 6 (10).

15 L. Xing, Y. Xiahou, P. Zhang, W. Du and H. Xia. Size Control Synthesis of Monodisperse, Quasi-Spherical Silver Nanoparticles To Realize Surface-Enhanced Raman Scattering Uniformity and Reproducibility. *ACS Appl. Mater. Interfaces* **2019**, 11 (19), 17637-17646.

16 M. E. Villanueva, A. E. Lanterna and R. V. Vico. Hydrophobic silver nanoparticles interacting with phospholipids and stratum corneum mimic membranes in Langmuir monolayers. *J. Colloid Interface Sci.* **2019**, 543, 247-255.

17 S. Agnihotri, S. Mukherji and S. Mukherji. Size-controlled silver nanoparticles synthesized over the range 5–100 nm using the same protocol and their antibacterial efficacy. *RSC Adv.* **2014**, 4 (8), 3974-3983.

18 X. Z. Lin, X. Teng and H. Yang. Direct Synthesis of Narrowly Dispersed Silver Nanoparticles Using a Single-Source Precursor. *Langmuir* **2003**, 19 (24), 10081–10085.

19 Z. Chen, T. Balankura, K. A. Fichthorn and R. M. Rioux. Revisiting the Polyol Synthesis of Silver Nanostructures: Role of Chloride in Nanocube Formation. *ACS Nano* **2019**, 13 (2), 1849-1860.

20 I. A. Wani, S. Khatoon, A. Ganguly, J. Ahmed, A. K. Ganguli and T. Ahmad. Silver nanoparticles: Large scale solvothermal synthesis and optical properties. *Mater. Res. Bull.* **2010**, 45 (8), 1033-1038.

21 Z. Li, X. Li, R. Wang, A. Campos Mata, C. S. Gerke, S. Xiang, A. Mathur, L. Zhang, D. Z. Lin, T. Li, K. N. Jayarapu, A. Liu, L. Gupta, A. I. Frenkel, V. S. Thoi, P. M. Ajayan, S. Roy, Y. Liu and Y. Liu. Electro-activated indigos intensify ampere-level CO_2 reduction to CO on silver catalysts. *Nat. Commun.* **2025**, 16 (1), 3206.

22 B. Endrődi, E. Kecsenovity, A. Samu, T. Halmágyi, S. Rojas-Carbonell, L. Wang, Y. Yan and C. Janáky. High carbonate ion conductance of a robust PiperION membrane allows industrial current density and conversion in a zero-gap carbon dioxide electrolyzer cell. *Energy Environ. Sci.* **2020**, 13 (11), 4098-4105.

23 C. Lim, S. Kim, J. H. Song, M. H. Han, Y.-J. Ko, K.-Y. Lee, J.-Y. Choi, W. H. Lee and H.-S. Oh. Breaking the current limitation of electrochemical CO_2 reduction via a silica-hydroxide cycle. *Energy Environ. Sci.* **2024**, 17 (17), 6215-6224.

24 L. Ma, Y. Wang, M. Zhang, J. Li, L. Zhang, X. Zhu, Q. Fu and Q. Liao. Rapid and stable electrolysis of CO_2-saturated bicarbonate solution to CO using a self-supported nano Ag mesh electrode. *J. Power Sources* **2025**, 641, 236874.

25 X. Kong, J. Zhao, Z. Xu, Z. Wang, Y. Wu, Y. Shi, H. Li, C. Ma, J. Zeng and Z. Geng. Dynamic Metal-Ligand Coordination Boosts CO_2 Electroreduction. *J. Am. Chem. Soc.* **2023**, 145 (27), 14903-14911.

26 X. Du, P. Zhang, G. Zhang, H. Gao, L. Zhang, M. Zhang, T. Wang and J. Gong. Confinement of ionomer for electrocatalytic CO_2 reduction reaction via efficient mass transfer pathways. *Natl. Sci. Rev.* **2024**, 11 (2), nwad149.

27 Y. Xu, J. P. Edwards, S. Liu, R. K. Miao, J. E. Huang, C. M. Gabardo, C. P. O'Brien, J. Li, E. H. Sargent and D. Sinton. Self-Cleaning CO_2 Reduction Systems: Unsteady Electrochemical Forcing Enables Stability. *ACS Energy Lett.* **2021**, 6 (2), 809-815.

28 Q. Xu, A. Xu, S. Garg, A. B. Moss, I. Chorkendorff, T. Bligaard and B. Seger. Enriching Surface-Accessible CO_2 in the Zero-Gap Anion-Exchange-Membrane-Based CO_2 Electrolyzer. *Angew. Chem. Int. Ed.* **2023**, 62 (3), e202214383.

29 W. D. Seider, J. D. Seader and D. R. Lewin. *Product and Process Design Principles: Synthesis, Analysis and Evaluation*. 3rd edn, John Wiley & Sons, (2009).

30 Qsonica, Replacement Probes., can be found under https://www.sonicator.com/products/replacement-probe?variant=537949274128 (accessed Jul. 2024).

31 YCharts, Potassium Chloride Spot Price., can be found under https://ycharts.com/indicators/potassium_chloride_spot_price (accessed Jul. 2024).

32 ChemAnalyst, Isopropyl Alcohol (IPA) Price Trend and Forecast., can be found under https://www.chemanalyst.com/Pricing-data/isopropyl-alcohol-31 (accessed Jul. 2024).

33 Echemi, Poly(vinylpyrrolidone) Market Price & Analysis., can be found under https://www.echemi.com/productsInformation/pd20150901219-polyvinylpyrrolidone.html (accessed Jul. 2024).

34 PharmaCompass, Silver Nitrate | Price | per kg | USD | can be found under https://www.pharmacompass.com/price/silver-nitrate (accessed Jul. 2024).

35 PharmaCompass, Sodium Borohydride | Price | per kg | USD |, can be found under https://www.pharmacompass.com/price/sodium-borohydride (accessed Jul. 2024).

36 PharmaCompass, Ethanol | Price | per kg | USD | can be found under https://www.pharmacompass.com/price/ethanol (accessed Jul. 2024).
